# Perm1 enhances Nrf2-driven antioxidant defense through Keap1 oxidation during myocardial ischemia/reperfusion injury

**DOI:** 10.1172/jci.insight.204132

**Published:** 2026-05-12

**Authors:** Shin-ichi Oka, Chun-Yang Huang, Masato Matsushita, Allen Sam Titus, Yasuki Nakada, Risa Mukai, Samta Veera, Youssef Mourad, Ghassan Yehia, Peter Romanienko, Yimin Tian, Peiyong Zhai, Junichi Sadoshima

**Affiliations:** 1 Department of Cell Biology and Molecular Medicine, Rutgers New Jersey Medical School, Newark, New Jersey, USA.; 2 Division of Cardiovascular Surgery, Department of Surgery, Taipei Veterans General Hospital, Taipei, Taiwan.; 3 Department of Medicine, School of Medicine, National Yang-Ming Chiao-Tung University, Taipei, Taiwan.; 4 Genome Editing Shared Resource, Rutgers Cancer Institute, New Brunswick, New Jersey, USA.

**Keywords:** Cardiology, Cell biology, Signal transduction

## Abstract

Ischemia/reperfusion (IR) enhances oxidative stress, leading to myocardial injury. Although Perm1 promotes cytoprotective mechanisms, the underlying mechanisms are poorly understood. Cysteine oxidation of Keap1 alleviates Cul3-mediated ubiquitination/degradation of Nrf2 and promotes antioxidant transcription. Here we show that Perm1 activates Nrf2 through cysteine oxidation of Keap1 and stabilization of Nrf2. Endogenous Perm1 was downregulated during IR, whereas the rescue of Perm1 reduced IR injury. Downregulation of Perm1 exacerbated oxidative stress, whereas upregulation of Perm1 alleviated it, accompanied by downregulation and upregulation of Nrf2-regulated antioxidant genes, respectively. Perm1 promoted oxidation of cysteine residues in Keap1, possibly through thiol-disulfide exchange reactions, which decreases Keap1-Nrf2 interaction and inhibits Cul3-mediated degradation of Nrf2. We identified Cys121 and Cys746 in Perm1 as critical for Keap1 oxidation and cardioprotection. Thus, Perm1 induces cysteine oxidation of Keap1, thereby conferring myocardial resistance to IR injury by inducing Nrf2 stabilization and transcriptional activation of antioxidant genes.

## Abstract 2

Oka et al. found Perm1 acts like a shield during heart attacks, reducing harmful stress and limiting heart damage.

## Graphical abstract

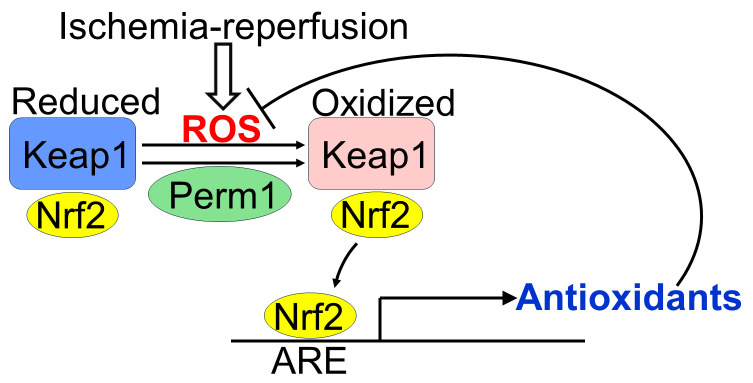

## Introduction

Recanalization of an occluded coronary artery is a standard treatment for acute myocardial infarction (1). Although coronary recanalization alleviates oxygen and nutrient starvation in the myocardium, reperfusion itself induces tissue damage, termed ischemia/reperfusion (IR) injury (2). When ischemic areas are perfused, reactive oxygen species (ROS) are produced and damage lipids, proteins, and DNA, thereby leading to mitochondrial damage and cell death. In order to minimize the myocardial injury caused by ROS, endogenous antioxidant systems, including superoxide dismutase (SOD), catalase, and NADPH-dependent antioxidants, including the glutathione (GSH) and thioredoxin 1 (Trx1) systems, are activated (3, 4).

Perm1 (PPARGC-1 and ESRR-induced regulator, muscle specific 1) is a protein expressed in skeletal and cardiac muscle that acts with peroxisome proliferator-activated receptor γ coactivator 1-α (PGC-1α) and estrogen-related receptors (ERRs) to regulate energy homeostasis genes and enhance mitochondrial biogenesis and oxidative capacity (5, 6). In skeletal muscle, Perm1 is upregulated in response to endurance exercise and increases resistance to fatigue without altering fiber-type composition (7). In cardiomyocytes, Perm1 acts as a positive regulator of ERRα, basal respiration, and ATP synthesis (8). Beyond muscle tissue, Perm1 is also expressed in adipose tissue, where it contributes to brite/beige adipocyte formation (9). Perm1 is localized in the cytosol, mitochondria, and nucleus, suggesting diverse functional roles (5, 10, 11). Reduced Perm1 expression has been reported in human cardiac tissue under pathological conditions, including heart failure (8). Perm1 possesses multiple cellular functions, many of which are cytoprotective and organ protective, across distinct subcellular compartments. However, the function of Perm1 in pathologically relevant conditions, such as IR injury, remains to be elucidated in the heart in vivo. Moreover, the molecular mechanisms through which Perm1 mediates protective functions remain poorly understood.

Nuclear factor erythroid 2-related factor 2 (Nrf2) promotes transcription of antioxidant enzymes through the antioxidant response element (ARE) (12). Kelch-like ECH-associated protein 1 (Keap1) is a component of the Cullin 3–based (Cul3-based) E3 ubiquitin ligase complex. Under basal conditions, Keap1 binds to Nrf2, thereby inducing Nrf2 ubiquitination and degradation. Under stress conditions, Keap1 releases Nrf2, which induces Nrf2 nuclear translocation and transcriptional activation. Although the availability of antioxidants in the heart is a critical determinant of IR injury, the molecular mechanisms through which Nrf2 is regulated during IR are not fully understood. Nrf2 mediates expression of antioxidant genes and pentose phosphate pathway genes, whereas it negatively regulates proinflammatory cytokines (13–15). The ability to control multiple salutary mechanisms makes Nrf2 a central factor in protecting the heart against IR injury.

Keap1 is rich in cysteines, and modification of its conserved cysteines affects Nrf2 degradation through Cul3-mediated ubiquitination. Cysteine oxidation affects the structure of Keap1 such that Nrf2 degradation is inhibited through either disassembly of the Keap1-Cul3 complex, Zn^2+^-coordination, or other unknown mechanisms (16, 17). The interaction of Keap1 and Nrf2 is also regulated by the interaction between Keap1 and other molecules with the ETGE motif, which is regulated through cysteine oxidation–independent mechanisms. Since Nrf2 is stabilized in the heart during IR, it is reasonable to speculate that the stabilization of Nrf2 during IR may be mediated through cysteine oxidation of Keap1. However, how cysteine oxidation of Keap1 is regulated during IR is poorly understood.

Using newly generated cardiomyocyte-specific *Perm1* KO (*Perm1* cKO) mice, we here show that endogenous Perm1 plays a protective role in the heart during IR injury. Endogenous Perm1 stabilizes Nrf2, thereby playing an essential role in protecting the heart against oxidative stress during myocardial IR. We further investigated the molecular mechanism through which Perm1 activates Nrf2. Our results suggest that Perm1 acts as an endogenous interacting protein to facilitate cysteine oxidation of Keap1, thereby inducing release of Nrf2 from Keap1 and stabilization of Nrf2.

## Results

### Loss of Perm1 exacerbates IR injury.

**Figure 1:**
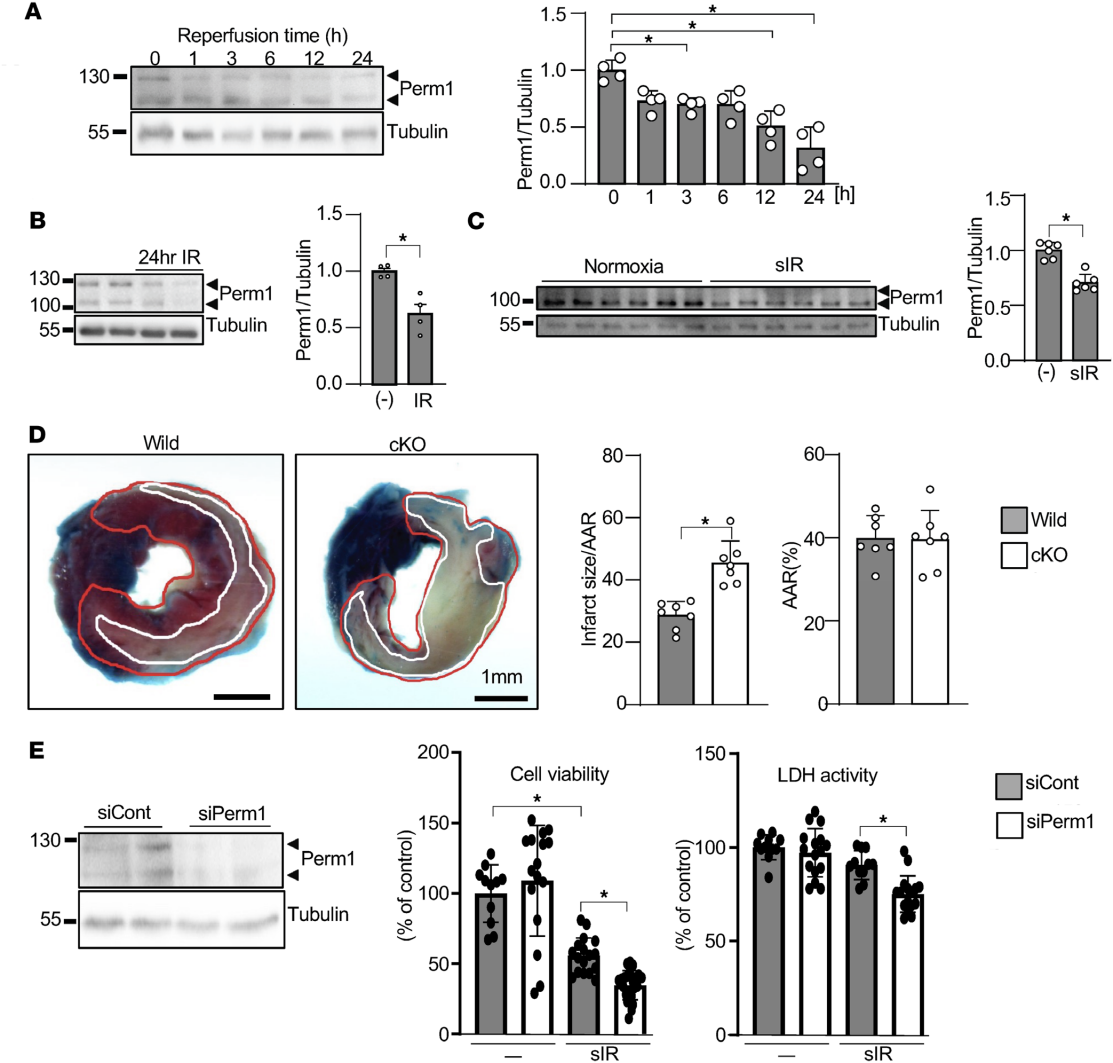
Loss of Perm1 exacerbates IR injury. (**A** and **B**) Perm1 was downregulated after IR. Mice were subjected to 30 minutes of ischemia and reperfusion as indicated. Perm1 levels were examined in heart lysates (**A**) and isolated cardiomyocytes (**B**). *n* = 4. (**C**) Perm1 was downregulated by sIR in cultured cardiomyocytes. *n* = 6. (**D**) Loss of Perm1 in cardiomyocytes exacerbated IR injury. Alcian blue and TTC staining was performed 24 hours after IR in *Perm1-*cKO mice. *n* = 7. Scale bar: 1 mm. (**E**) Knockdown of Perm1 exacerbated sIR-induced cardiomyocyte death. Downregulation of Perm1 by siRNA was consistently observed across multiple independent experiments (Figure 6A). Cultured cardiomyocytes were transfected with either siScramble or siPerm1 and then subjected to normoxia or sIR. Cell viability was evaluated with CellTiter-Blue assays (left) and LDH activity assays (right). *n* = 11–24 (cell viability) and 11–16 (LDH activity). Statistical significance was determined with 1-way ANOVA (**A** and **E**) and Student’s *t* test (**B**–**D**). *P < 0.05.

The level of Perm1 in the heart decreased time dependently between 1 and 24 hours after IR (Figure 1A). Note that Perm1 shows multiple bands, ranging from 100 to 130 kD, resulting from alternative start codons and possibly other unknown mechanisms (18), in cardiomyocytes. The bands approximately at 130 kD and 100 kD both represent Perm1 in the mouse heart, as they were not observed in *Perm1*-KO mice (Supplemental Figure 1A; supplemental material available online with this article; https://doi.org/10.1172/jci.insight.204132DS1). Both forms of Perm1 were downregulated by IR (Figure 1A and Supplemental Figure 1A). Downregulation of Perm1 24 hours after IR was also observed in cardiomyocytes isolated from the mouse heart (Figure 1B). Perm1 was also downregulated by simulated IR (sIR) (19) in neonatal rat ventricular myocytes (NRVMs) (Figure 1C), suggesting that the effect is cell autonomous. Based on the continuous downregulation of Perm1 up to 24 hours after reperfusion, and because this time point is widely used for infarct size assessment in experimental IR models, we selected the 24-hour time point for subsequent analyses throughout the study. Accordingly, WT and homozygous *Perm1-*KO mice were subjected to 30 minutes of myocardial ischemia followed by 24 hours of reperfusion. The infarct area, determined by triphenyl tetrazolium chloride (TTC) staining, corrected by the area at risk (AAR), determined by Alcian Blue Dye staining, was significantly larger in *Perm1*-KO mice than in WT mice (Supplemental Figure 1B). To investigate the role of Perm1 in cardiomyocytes, we generated *Perm1-*cKO mice by crossing *Perm1^fl/fl^* mice with *Myh6-Cre* mice. Downregulation of Perm1 expression was confirmed in heterozygous *Perm1-*cKO mice (Supplemental Figure 2A). At baseline, *Perm1*-cKO mice exhibited normal cardiac morphology and function, as assessed by echocardiography (Supplemental Figure 2B), including preserved contractile function (Supplemental Figure 2C), left ventricular (LV) chamber dimensions (Supplemental Figure 2D), and wall thickness (Supplemental Figure 2E). No evidence of cardiac hypertrophy was observed, as indicated by an unaltered LV weight/tibia length (TL) ratio (Supplemental Figure 2F). *Perm1-*cKO mice exhibited a larger infarct area 24 hours after IR than WT mice (Figure 1D). To evaluate whether the loss of Perm1 function affects cardiomyocyte death in response to IR, primary NRVMs transfected with control siRNA (siControl) or siRNA targeting *Perm1* (siPerm1) were subjected to sIR. sIR significantly decreased cell viability and the level of lactate dehydrogenase (LDH) in NRVMs transfected with either siControl or siPerm1, but the reduction was significantly greater in NRVMs transfected with siPerm1 than in those with siControl (Figure 1E). These results suggest that Perm1 protects the heart and cardiomyocytes against IR injury.

### Overexpression of Perm1 protects the heart against IR.

**Figure 2:**
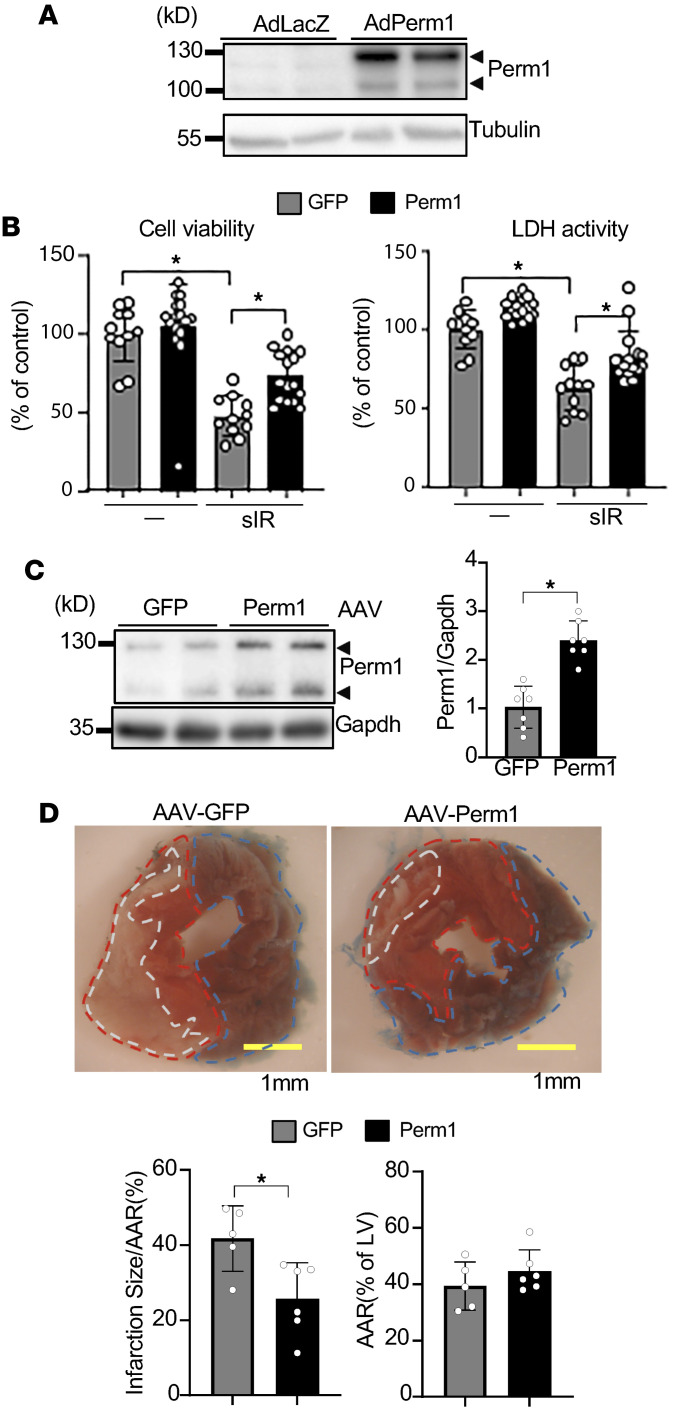
Perm1 overexpression attenuates IR injury. (**A**) Perm1 upregulation with Ad-Perm1 was verified by Western blot analysis. Similar results were consistently observed across multiple independent experiments (Figure 5, A, G, and H, and Figure 6, C, and D). (**B**) Perm1 overexpression attenuated sIR-induced cell death. Cultured cardiomyocytes were transduced with either Ad-GFP or Ad-Perm1 and subjected to sIR. Cell viability, evaluated with CellTiter-Blue assays (left), and LDH assays (right). (**C**) Perm1 upregulation with AAV-Perm1 was verified by Western blot analysis. (**D**) Perm1 overexpression attenuated IR injury. Four weeks after AAV transduction, mice were subjected to IR for 24 hours. Infarct area was assessed by TTC staining. Scale bars: 1 mm. Statistical significance was determined with Student’s *t* test (**C** and **D**) and ANOVA (**B**). *n* = 11–16 (**B**), 7 (**C**), and 5–6 (**D**). *P < 0.05.

To test whether Perm1 overexpression protects cardiomyocytes against IR, NRVMs were transduced with either Ad-LacZ or Ad-Perm1 and subjected to sIR. Perm1 upregulation with Ad-Perm1 was verified (Figure 2A). sIR significantly reduced cell viability and the LDH level in NRVMs in the presence of either LacZ or Perm1 overexpression. However, the decreases in cell viability and LDH levels were significantly smaller in NRVMs in the presence of Perm1 overexpression than in the presence of LacZ overexpression, suggesting that Perm1 confers resistance to IR injury to NRVMs in a cell-autonomous manner (Figure 2B).

To test whether gain of Perm1 function protects the heart in vivo, Perm1 was overexpressed in a cardiomyocyte-specific manner through AAV9-mediated gene transfer. Transduction of AAV9-cTNT-Perm1 induced 2.2-fold overexpression of Perm1 in the heart compared with transduction of AAV9-cTNT-GFP (Figure 2C). Although the level of Perm1 in the heart was decreased after 4 hours of IR, transduction of AAV9-cTNT-Perm1 restored the level of Perm1 in the heart to that of the heart without IR (Supplemental Figure 4A). In this condition, the infarct area/AAR was significantly decreased, suggesting that overexpression of Perm1 protects the heart against myocardial injury during IR (Figure 2D). Furthermore, the significant reduction of IR injury as a result of the rescue of IR-induced downregulation of Perm1 in cardiomyocytes suggests that IR-induced downregulation of endogenous Perm1 plays a critical role in mediating myocardial injury.

### Exacerbation of IR injury in Perm1-cKO mice was not mediated through downregulation of ERR targets.

Since the results thus far suggest that downregulation of Perm1 exacerbates IR injury in the heart, we investigated the underlying mechanisms. We first investigated the role of ERRs, since Perm1 positively regulates transcription of *Ppara, Pparb, Pparg, Esrra, Esrrb*, and *Esrrg*, thereby upregulating their targets, including genes involved in mitochondrial biogenesis and oxidative phosphorylation (8, 18, 20, 21). The mRNA and protein levels of these factors were decreased at baseline and remained decreased (about 40% of control levels) during IR in *Perm1*-cKO mice compared with in control mice (Supplemental Figure 3A). Importantly, IR injury was alleviated in *Esrrg* cKO (heterozygous) mice compared with in respective control mice (Supplemental Figure 3B). Moreover, cardiomyocyte-specific rescue of ERRα with AAV9-cTNT-ERRα exacerbated IR injury in WT mice (Supplemental Figure 3C). These results suggest that downregulation of Perm1 target genes, such as *Esrrg*, in response to IR caused by downregulation of endogenous Perm1 is an adaptive response to minimize myocardial injury and does not appear to mediate the exacerbation of IR injury in *Perm1*-cKO mice (22–24). Our results are also consistent with previous studies showing that promoting fatty acid oxidation during IR is detrimental for the heart (22–24). Taken together, it is highly unlikely that the exacerbation of myocardial injury in mice with a loss of Perm1 function is mediated through downregulation of genes involved in energy metabolism.

### Perm1 KO mouse hearts exhibit greater oxidative stress after IR than WT mouse hearts.

**Figure 3:**
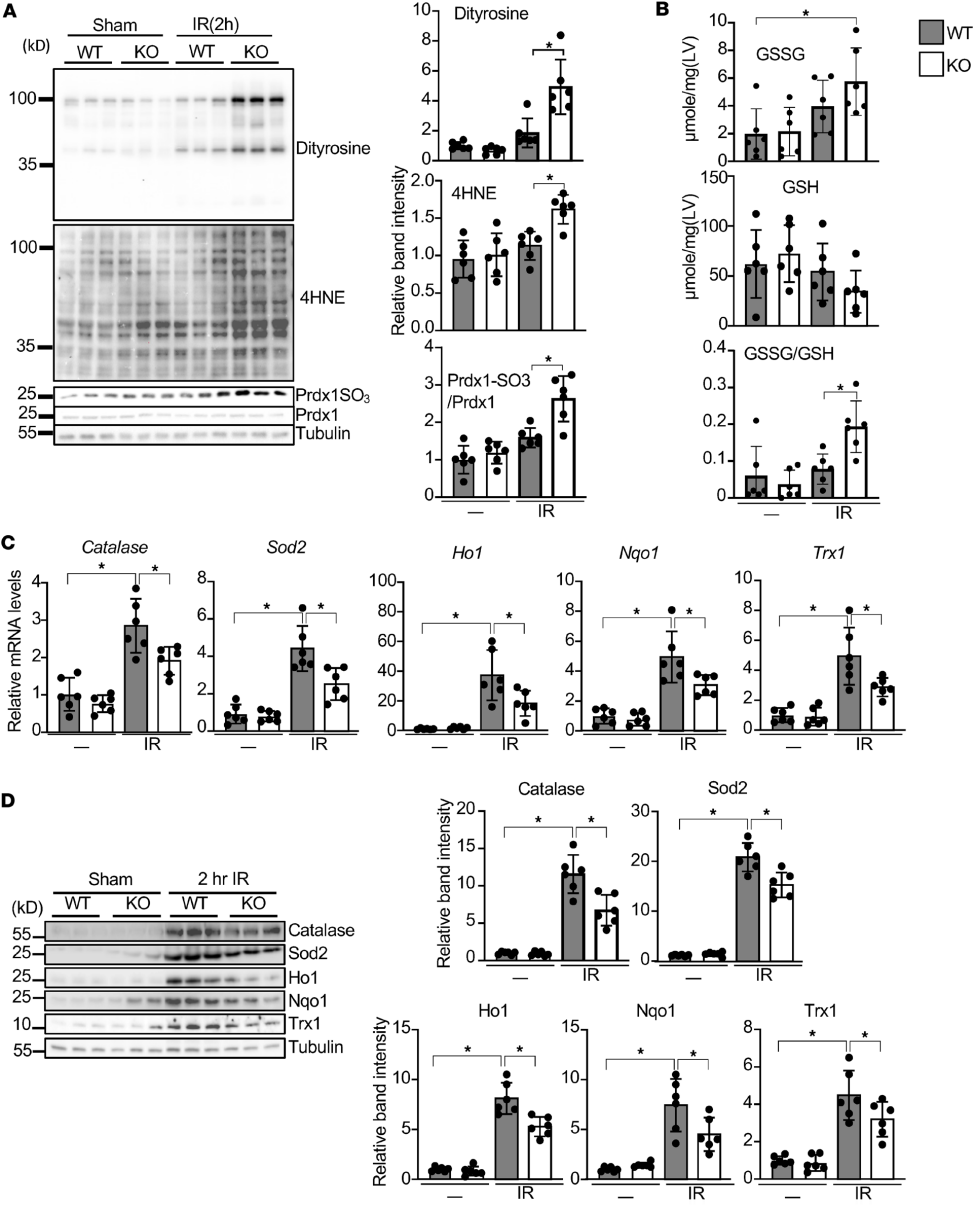
Loss of Perm1 exacerbates IR-induced oxidative stress. (**A**) IR-induced oxidative stress was promoted in *Perm1*-KO mice. Oxidative stress markers, including Dityrosine, 4HNE, and Prdx1-SO3, were examined in the heart. (**B**) The levels of GSSG and GSH and the GSSG/GSH ratio were examined. (**C** and **D**) The mRNA (**C**) and protein (**D**) levels of catalase, superoxide dismutase 2 (SOD2), heme oxygenase 1 (HO1), NADH quinone dehydrogenase 1 (NQO1), and thioredoxin 1 (Trx1) were examined. *Perm1*-KO and WT mice were subjected to 30 minutes of ischemia and reperfusion for 2 hours. Statistical significance was determined by 1-way ANOVA. *n* = 6. *P < 0.05.

Since IR injury is accompanied by increased oxidative stress (2), we next assessed oxidative stress in the *Perm1*-KO mice. The levels of oxidative stress markers, including dityrosine, 4HNE, and sulfonated Prdx1 (Prdx1-SO_3_), were significantly greater in *Perm1-*KO mice than in WT mice after IR (Figure 3A). The level of GSSG, an oxidized form of glutathione (GSH), was significantly increased in *Perm1*-KO mice after IR (Figure 3B), and the GSSG/GSH ratio was greater in *Perm1*-KO mice than in WT mice after IR (Figure 3B). Major antioxidants, namely catalase (*Cat*), *Sod2*, heme oxygenase 1 (*Ho1*), NADH quinone dehydrogenase 1 (*Nqo1*), and *Trx1*, were significantly upregulated at both the mRNA and protein levels after 2 hours of IR in WT mice, whereas their upregulation was significantly attenuated in *Perm1*-KO mice (Figure 3, C and D). These results suggest that endogenous Perm1 plays an essential role in mediating the upregulation of antioxidants in the heart during IR.

On the other hand, transduction of AAV9-cTNT-Perm1 ameliorated oxidative stress, as evidenced by attenuation of dityrosine, 4HNE and Prdx1-SO_3_, in response to 4 hours of IR (Supplemental Figure 4A). Perm1 overexpression promoted IR-induced upregulation of antioxidants, including Catalase, Sod2, Ho1, and Nqo1 (Supplemental Figure 4B). Taken together, these results indicate that Perm1 overexpression induces upregulation of antioxidants.

### Perm1 promotes Nrf2 activation in response to IR.

**Figure 4:**
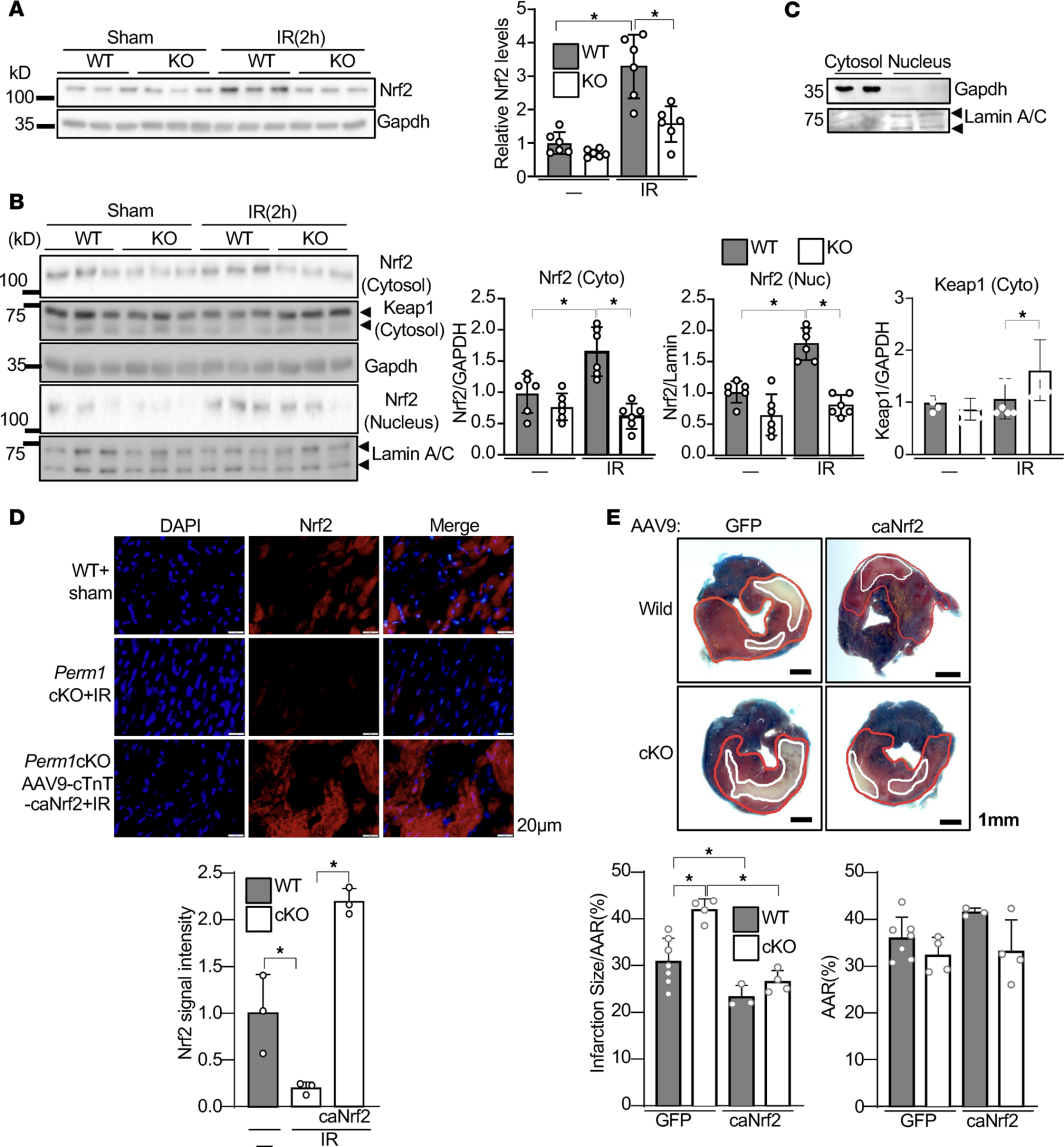
Perm1 promotes Nrf2 activation. (**A**) IR-induced Nrf2 upregulation was inhibited in *Perm1*-KO mice. *Perm1-*KO and WT mice were subjected to 30 minutes of ischemia and reperfusion for 2 hours. The levels of Nrf2 were examined in heart homogenates. (**B** and **C**) Heart homogenates were fractionated to isolate cytosolic and nuclear fractions. Loss of Perm1 inhibited IR-induced Nrf2 upregulation and nuclear translocation. The levels of indicated proteins were examined in the cytosolic and nuclear fractions after IR (**B**). GAPDH and Lamin A/C represent cytosolic and nuclear markers, respectively (**C**). (**D**) Verification of caNrf2 expression with AAV-cTNT-caNrf2. WT and *Perm1-*cKO mice were transduced with either AAV9-cTNT-LacZ or AAV9-cTNT-caNrf2. Three weeks later, IR was applied. Scale bar: 20 μm. (**E**) Constitutively active Nrf2 (caNrf2) normalizes IR injury in *Perm1-*cKO mice. Scale bar: 1 mm. Statistical significance was determined by ANOVA (**A**, **C**, **D** and **E**). *n* = 6 (**A** and **C**), 3 (**D**) and 3–6 (**E**). *P < 0.05.

Because Nrf2 drives the transcription of the antioxidant genes examined in this study (13), we tested whether endogenous Perm1 positively regulates Nrf2-dependent transcription during IR in the heart. In WT mouse hearts, Nrf2 expression was significantly increased 2 hours after IR, despite a concurrent reduction in Perm1 expression. However, this IR-induced upregulation of Nrf2 was blunted in *Perm1-*KO hearts, where Perm1 expression is persistently reduced (Figure 4A). These results suggest that Nrf2 is regulated by both Perm1-dependent and Perm1-independent mechanisms. The latter likely include oxidative stress–mediated transcriptional activation of Nrf2 via the aryl hydrocarbon receptor (25) and NF-κB (26). Thus, although alternative pathways can stimulate Nrf2 during IR, sustained loss of Perm1 limits full Nrf2 activation in the heart.

Subcellular fractionation of heart homogenates showed that the level of Nrf2 in both the nuclear and cytosolic fractions after IR was significantly greater in WT mouse hearts than in *Perm1*-KO hearts. In contrast, the level of Keap1, a protein binding to Nrf2 and inducing degradation of Nrf2 through a proteasome-dependent mechanism (13), in the cytosol was significantly greater in *Perm1*-KO hearts than in WT mouse hearts, consistent with its role in Nrf2 sequestration in the cytosol (Figure 4B). The nuclear fraction contained little GAPDH and the cytosolic fraction contained little Lamin A/C, confirming the purity of each fraction (Figure 4C).

### Nrf2 activation prevents exacerbation of IR injury in Perm1-KO mice.

To test whether Perm1 protects the heart against IR injury through Nrf2 activation, we conducted a rescue experiment. Since downregulation of endogenous Perm1 may allow the Keap1-Cul3 complex to mediate degradation of both endogenous and exogenous Nrf2, we used an Nrf2 mutant (delta N Nrf2) in which the first 79 amino acids are removed to prevent the Nrf2-Keap1 interaction and degradation of Nrf2, thus acting as a constitutively active mutant (27). AAV9-cTNT–constitutively active Nrf2 (caNrf2) or AAV9-cTNT-GFP was transduced into WT and *Perm1-*cKO mice. We confirmed that transduction with AAV9-cTNT-caNrf2 increases expression of caNrf2 in the mouse heart with immunostaining (Figure 4D). The exacerbation of IR injury in *Perm1-*cKO mice was inhibited in the presence of caNrf2 but not GFP (Figure 4E).

The IR injury observed in *Perm1*-KO mice was also rescued in the presence of ML334, a cell-permeable inhibitor of Keap1-Nrf2 interaction (28) (Supplemental Figure 5, A and B). ML334 treatment stabilized Nrf2 in *Perm1*-KO hearts (Supplemental Figure 5C). ML334 also significantly attenuated siPerm1-induced downregulation of Nrf2 in cultured NRVMs and reversed decreases in cell viability in the presence of siPerm1 (Supplemental Figure 5, D and E). These results suggest that the exacerbation of IR injury and cardiomyocyte cell death in response to downregulation of Perm1 is mediated through downregulation of Nrf2.

### Perm1 physically interacts with Keap1.

**Figure 5:**
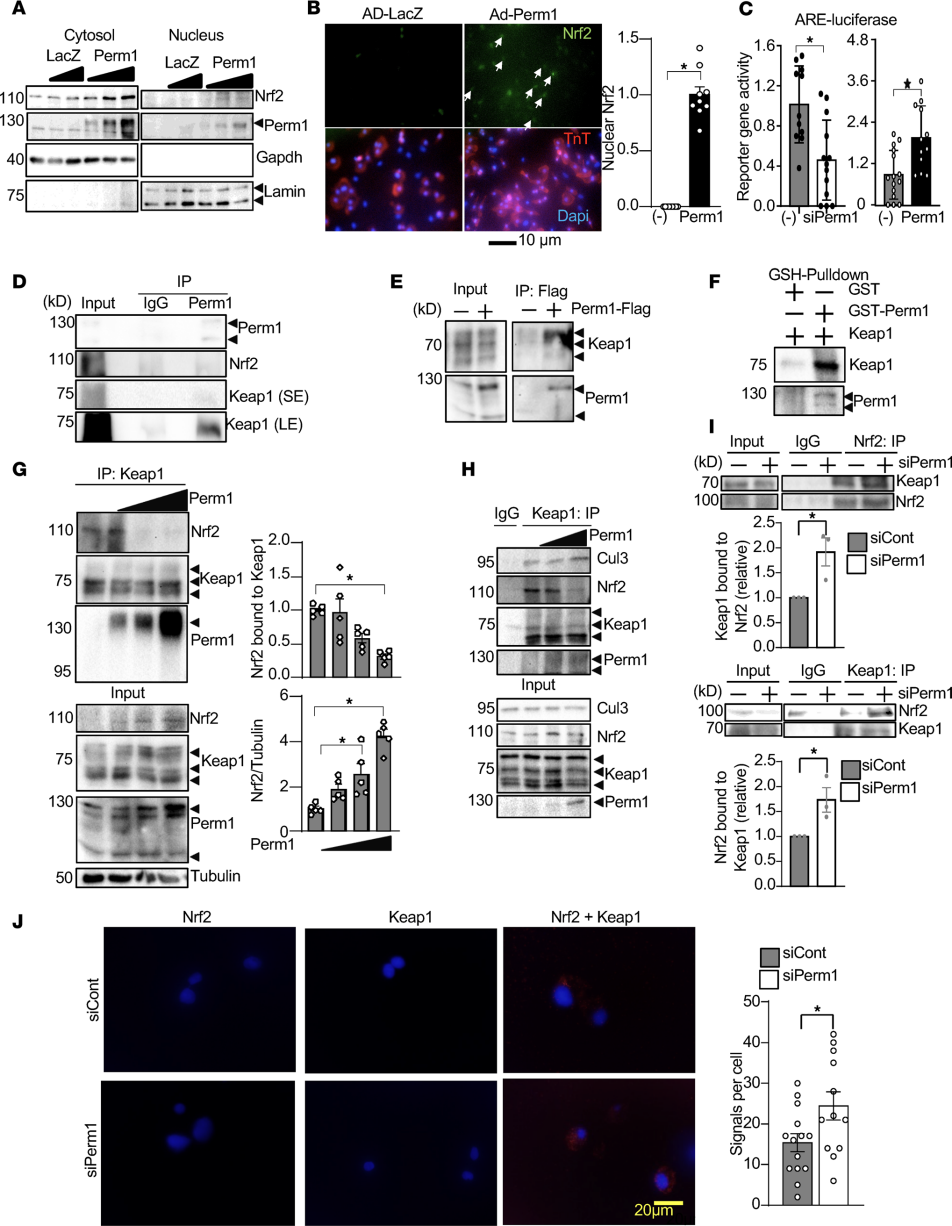
Perm1 interacts with Keap1. (**A** and **B**) Perm1 overexpression promoted nuclear translocation of Nrf2 in cultured cardiomyocytes. Cardiomyocytes were transduced with Ad-Perm1. The levels of indicated proteins were examined in the cytosolic and nuclear fractions. Nuclear translocation of Nrf2 was assessed by immunostaining. (**C**) Perm1 stimulated reporter gene activity driven by the antioxidant response element (ARE). Statistical significance was determined by Student’s *t* test (**B** and **C**). *n* = 10–15 (**C**). *P < 0.05. (**D**–**F**) Perm1 binds to Keap1. (**D**) Coimmunoprecipitation assays were performed with anti-Perm1 antibody using cultured cardiomyocytes. SE, Short exposure; LE, Long exposure. (**E**) Adenovirus vector carrying Flag-Perm1 was transduced into cardiomyocytes. Flag-Perm1 was immunoprecipitated with anti-Flag antibody. (**F**) Perm1 bound to Keap1 in vitro. GST-pulldown assays were performed with GST-Perm1 and recombinant Keap1. (**G**) Perm1 inhibited the binding of Keap1 to Nrf2. Keap1 was immunoprecipitated from cardiomyocytes with Perm1 overexpression. (**H**) Perm1 did not inhibit the binding of Keap1 to Cul3. (**I** and **J**) Loss of Perm1 enhanced the binding of Keap1 and Nrf2. Cultured cardiomyocytes transfected with either siControl or siPerm1 were subjected to sIR. After 30 minutes of hypoxia and 1 hour of reoxygenation, coimmunoprecipitation assays (**I**) and PLA assays (**J**) were performed with anti-Nrf2, anti-Keap1, and control IgG antibodies. Statistical significance was determined by ANOVA (**G**) and Student’s *t* test (**I** and **J**). *n* = 5 (**G**), 3 (**I**) and 12-14 (**J**). *P < 0.05. (**A**, **D**, **E**, **F** and **H**). Representative data are shown from 2 independent experiments. Scale bars: 10 μm (B) and 20 μm (**J**).

In order to evaluate the effect of Perm1 upon Nrf2 activation, we first examined the extent to which Perm1 upregulates nuclear Nrf2 in cultured NRVMs. Overexpression of Perm1 using Ad-Perm1 upregulated Nrf2 in both the cytosol and nucleus (Figure 5A). The increase in nuclear Nrf2 was also confirmed by immunostaining (Figure 5B). We then conducted reporter gene assays, which showed that downregulation of Perm1 significantly reduced, whereas its upregulation increased, the activity of an ARE-driven reporter gene at baseline (Figure 5C). In order to evaluate whether Perm1 binds to Nrf2 or Keap1 in cardiomyocytes, we conducted coimmunoprecipitation assays using cultured cardiomyocytes. Although Nrf2 was not significantly enriched in the anti-Perm1 antibody immunoprecipitate compared with the control IgG immunoprecipitate, Keap1 was enriched in the Perm1 immunoprecipitate but not in the control IgG immunoprecipitate (Figure 5D). Keap1 was also immunoprecipitated with anti-Flag antibody when Flag-Perm1 was expressed with an adenovirus vector (Figure 5E). These results suggest that Perm1 interacts with Keap1, but not Nrf2, in cardiomyocytes. To examine whether Perm1 directly binds to Keap1, in vitro binding assays were performed with bacterially expressed recombinant GST-fused Perm1 and Keap1. GST-pulldown assays demonstrated direct binding of Perm1 and Keap1 (Figure 5F). Keap1 interacts with Nrf2 in the cytosol and promotes ubiquitination and degradation of Nrf2 (29). Thus, we hypothesized that Perm1 might interfere with this process through direct interaction with Keap1, whereas downregulation of Perm1 may promote the Keap1-Nrf2 interaction and consequent degradation of Nrf2. To test whether Perm1 competitively inhibits the binding of Keap1 to Nrf2, coimmunoprecipitation assays were performed with anti-Keap1 antibody. The binding of Keap1 to Nrf2 was inhibited and Nrf2 was upregulated by Perm1 in a dose-dependent manner (Figure 5G). In contrast, Perm1 did not compete with the binding of Keap1 to Cul3, the E3 ligase that forms a complex with Keap1 (Figure 5H). The physical interaction between Keap1 and Nrf2 was enhanced in cardiomyocytes subjected to sIR in the presence of Perm1 knockdown (Figure 5I). Likewise, proximity ligation assays indicated that interaction between Keap1 and Nrf2 in cardiomyocytes in the presence of sIR was increased in the cytosol of NRVMs in the presence of Perm1 knockdown (Figure 5J). These results suggest that Perm1 inhibits the binding of Keap1 to Nrf2.

### Perm1 promotes Keap1 oxidation.

**Figure 6:**
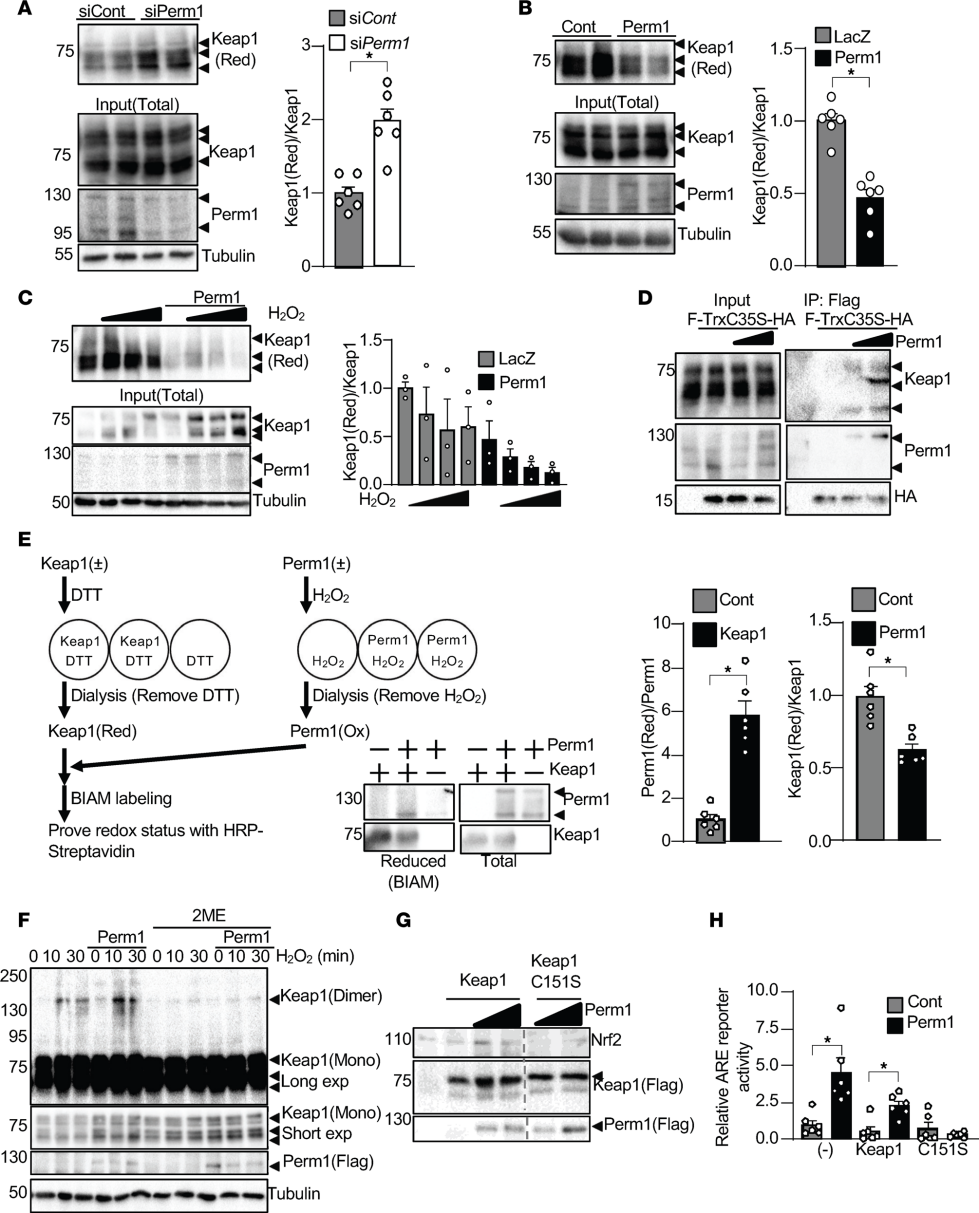
Perm1 promotes Keap1 oxidation. (**A** and **B**) Perm1 promoted Keap1 oxidation. Protein thiols in their reduced form were labeled with BIAM in cardiomyocytes with Perm1 knockdown (**A**) and overexpression (**B**). BIAM-labeled proteins were pulled down with avidin-agarose. (**C**) Perm1 promoted H_2_O_2_-induced Keap1 oxidation. Cardiomyocytes transduced with Ad-Perm1 were treated with H_2_O_2_ (0, 10, 30 and 100 μmol/L) for 30 minutes. BIAM pulldown assays were performed. (**D**) Perm1 promoted the interaction between Keap1 and a substrate trapping mutant of Trx1 (Flag-Trx1C35S-HA). Cardiomyocytes were transduced with Ad-Perm1 and Ad-Flag-Trx1C35S-HA. Flag-Trx1C35S-HA was immunoprecipitated with anti-Flag antibody. (**E**) Perm1 oxidized Keap1 in vitro. Recombinant Keap1 reduced with DTT and recombinant Perm1 oxidized with H_2_O_2_ were incubated together and subjected to BIAM binding assays. (**F**) Perm1 promoted intermolecular disulfide bond formation in Keap1. Cardiomyocytes transduced with Ad-Perm1 were treated with H_2_O_2_. SDS-PAGE under nonreducing conditions was performed. (**G**) Keap1 C151S mutant inhibited Perm1-induced Nrf2 upregulation. (**H**) Keap1 C151S mutant inhibited Perm1-induced reporter gene activity driven by ARE. Statistical significance was determined by Student’s *t* test (**A**, **B** and **E**) and ANOVA (**H**). *n* = 5 (**A** and **B**) and 6 (**E** and **H**). *P < 0.05. (**E**–**G**) Representative data are shown from 3 (**E** and **F**) and 2 (**G**) independent experiments.

Nrf2 is stabilized when it dissociates from Keap1. One mechanism that promotes the stabilization of Nrf2 is Keap1 oxidation (30). To test whether Perm1 promotes Keap1 oxidation, the redox status of Keap1 was assessed by pulldown with biotin-labeled iodoacetamide (BIAM), which irreversibly binds to reduced thiols (31–33). Knockdown of Perm1 promoted BIAM binding to Keap1 (cysteine reduction) (Figure 6A), and Perm1 overexpression reduced BIAM binding to Keap1 (cysteine oxidation) (Figure 6B). These results indicate that modulation of Perm1 expression alone is sufficient to alter the redox state of Keap1.

We also investigated the relative effect of Perm1 overexpression and H_2_O_2_ upon cysteine oxidation of Keap1 with BIAM assays. H_2_O_2_ dose-dependently induced oxidation of Keap1, consistent with a recent report (34). Notably, Perm1 overexpression resulted in greater Keap1 oxidation than the highest concentration of H_2_O_2_ tested (Figure 6C). Furthermore, Perm1 and H_2_O_2_ had additive effects upon cysteine oxidation of Keap1. These findings indicate that Perm1 and oxidative stress act as parallel inputs, rather than a single linear pathway, in regulating the redox state of Keap1. This may explain the fact that Perm1 can influence Nrf2 levels beyond just oxidative stress alone. Interestingly, the enhancement of Keap1 oxidation by Perm1 was accompanied by a corresponding increase in Keap1 abundance, suggesting a potential link between cysteine oxidation and the stabilization of Keap1. Keap1 abundance is known to be regulated by the ubiquitin-proteasome system (35) and autophagy (36). However, the mechanisms that control Keap1 stability under these conditions, and how cysteine oxidation influences these degradation pathways, remain to be determined.

Oxidized Keap1 may be reduced by Trx1 (37). In addition, Trx1 is a possible Perm1 binding protein (21). We hypothesized that Perm1 competes with the binding of Trx1 to Keap1, thereby oxidizing Keap1. To test this hypothesis, Flag-tagged Trx1C35S, a substrate trapping mutant (33), was expressed in NRVMs together with Perm1. Contrary to our hypothesis, Perm1 actually promoted the interaction between Trx1C35S and Keap1 (Figure 6D). This is consistent with the notion that Perm1 oxidizes Keap1, thereby promoting the disulfide bond formation between Keap1 and Trx1C35S.

To test whether Perm1 directly oxidizes Keap1 in vitro, a redox reaction was performed with recombinant proteins. As shown in Figure 6E, recombinant Perm1 was oxidized with H_2_O_2_ and recombinant Keap1 was reduced by DTT. After the H_2_O_2_ and DTT were completely removed by dialysis, reduced thiols were labeled with BIAM. Keap1 was oxidized by Perm1, whereas Perm1 was reduced by Keap1. Under nonreducing SDS-PAGE conditions (Supplemental Figure 6), the addition of oxidized Perm1 to reduced Keap1 induced a shift of Keap1 toward higher molecular weight species, accompanied by a reduction in lower molecular weight forms. This shift is consistent with increased intermolecular disulfide bond formation. Conversely, Perm1 exhibited a shift toward lower molecular weight species in the presence of reduced Keap1. These findings are consistent with thiol-disulfide exchange between Perm1 and Keap1, suggesting reciprocal redox interactions between the two proteins.

### Oxidation of Cys151 plays an important role in mediating Perm1-induced stabilization of Nrf2.

Cysteine residues of Keap1 are oxidized in the presence of electrophiles and oxidative stress in a stimulus-specific manner, which leads to structural alterations that induce dissociation of Cul3 and Nrf2 from Keap1 (12). Oxidation of Keap1 at Cys151 leads to intermolecular disulfide bond formation, which is highly critical for the release of Cul3 and stabilization of Nrf2 (35). Thus, we investigated whether Perm1 induces disulfide bond formation in Keap1. In NRVMs, both Perm1 and H_2_O_2_ induced molecular weight shifts of Keap1 to around 150 kDa in nonreducing SDS-PAGE. The shifted band of Keap1 disappeared in the presence of 2-mercaptoethanol (2ME), indicating that the band shift is due to disulfide bond formation (Figure 6F). Because the formation of Keap1 disulfides at around 150 kDa is Cys151 dependent (16, 38), these results suggest that Perm1 induces oxidation of Cys151. Furthermore, Perm1-induced upregulation of Nrf2 and the nuclear transcriptional activity of Nrf2 were inhibited in the presence of Keap1 (C151S) (Figure 6, G and H), suggesting that oxidation of Keap1 at Cys151 is required for Perm1-induced activation of Nrf2.

### Cys121 and Cys746 residues in Perm1 play an important role in mediating oxidation of Keap1.

**Figure 7:**
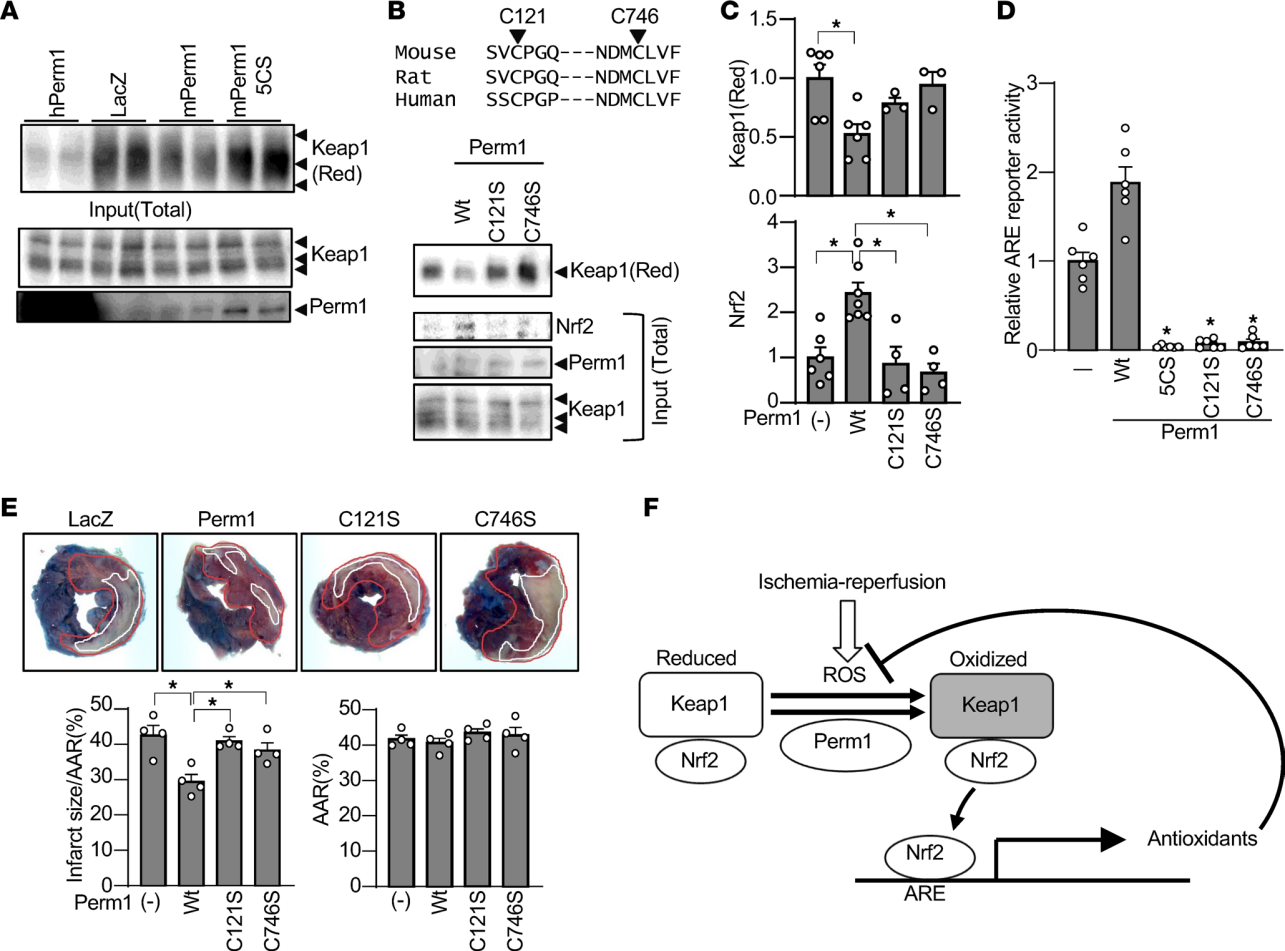
Cys121 and Cys746 residues in Perm1 mediate Keap1 oxidation. (**A**) The redox status of Keap1 was assessed using BIAM labeling in cultured cardiomyocytes transduced with adenoviral vectors encoding human Perm1 (hPerm1), mouse Perm1 (mPerm1), or a 5CS mutant of mouse Perm1, in which the five cysteine residues conserved between human and mouse Perm1 were substituted with serine. Representative data are shown from 2 independent experiments (**B** and **C**) Cys121 and Cys746 in Perm1 mediate Keap1 oxidation. The redox status of Keap1 and Nrf2 levels were examined in cardiomyocytes transduced with Ad-Perm1 and its CS mutants. (**D**) Cysteine mutants of Perm1 failed to stimulate ARE-mediated reporter gene activity. (**E**) Cys121 and Cys746 mediate Perm1-induced cardioprotection. Ad-LacZ, Ad-Perm1, Ad-Perm1(C121S), or Ad-Perm1(C746S) was injected into the heart, using a 30-gauge needle into the LV free wall (1 × 10^9^ particles/30 μL), as described recently (48). Three days later, mice were subjected to IR, and the infarct size was evaluated. Statistical significance was determined by ANOVA (**C**–**E**). *n* = 4–7 (**C**), 6 (**D**) and 4 (**E**). *P < 0.05. (**F**) Schematic representation of hypothetical model. Perm1 promotes Keap1 oxidation, thereby inducing expression of antioxidant enzymes via Nrf2.

Since cysteine residues of Perm1 are reduced when cysteines in Keap1 are oxidized, we investigated which cysteines in Perm1 are involved in this reaction. Sequence alignment revealed 5 conserved cysteine residues shared between human and mouse Perm1. To test whether these mediate Keap1 oxidation, we compared WT human Perm1 (hPerm1) with a mutant mouse Perm1 (mPerm1CS) in which all 5 conserved cysteines were mutated to serine. As shown in Figure 7A, hPerm1 overexpression induced Keap1 oxidation, whereas the mPerm1CS mutant did not. These findings indicate that Perm1 in both humans and mice harbors redox-active cysteine residues essential for Keap1 oxidation. Among the 5 cysteines, we focused on Cys121 and Cys746 because Cys121 is adjacent to proline, a sequence often associated with the high cysteine reactivity, while Cys746 is flanked by hydrophobic residues, which facilitates oxidation. Notably, Cys746 is also conserved in zebrafish and Drosophila. As shown in Figure 7, B and C, Keap1 oxidation by Perm1 was abolished in the C121S and C746S mutants. Similarly, Perm1-induced activation of the Nrf2 reporter gene was lost in these mutants, as well as in mPerm1CS (Figure 7D). These results suggest that Cys121 and Cys746 are required for Keap1 oxidation. To determine whether these residues mediate Perm1’s protective effects against IR injury, we overexpressed Perm1 variants in mouse hearts using adenoviral vectors. Perm1 protected the heart from IR injury, as shown by TTC staining, but this protection was abolished in the presence of Perm1 C121S and C746S mutants (Figure 7E). Collectively, these findings indicate that Perm1 promotes Keap1 oxidation via Cys121 and Cys746, thereby contributing to cardioprotection against IR injury.

## Discussion

We demonstrate that endogenous Perm1 plays an essential role in protecting the heart against IR injury. Perm1 promotes cysteine oxidation of Keap1 and disrupts the Keap1-Nrf2 interaction, thereby protecting cardiomyocytes from oxidative stress through Nrf2-mediated upregulation of antioxidant genes (Figure 7F). During IR, Perm1 expression is downregulated, which suppresses the upregulation of genes involved in oxidative phosphorylation, likely as a compensatory response to limit myocardial injury. However, reduced Perm1 levels also result in insufficient Nrf2 activation and inadequate induction of antioxidant genes, including *Sod2* and *Cat* (13), ultimately exacerbating myocardial damage. Mechanistically, Perm1 activates Nrf2 by inducing Keap1 oxidation, which facilitates Nrf2 dissociation from Keap1 and enhances Nrf2 stabilization.

### Perm1 as an endogenous regulator of Keap1.

This study suggests that Perm1 represents an endogenous regulator of Keap1 that promotes Keap1 oxidation, thereby potentiating oxidative stress–induced Keap1 oxidation and Nrf2 activation. Nrf2 activation during IR is attenuated in *Perm1-*cKO mice compared with WT mice. Modulation of Perm1 expression was sufficient to alter the redox status of Keap1. Consistently, there was a trend toward reduced cytosolic and nuclear Nrf2 levels in *Perm1*-KO mice under basal conditions (Figure 4B), and knockdown of Perm1 significantly decreased basal Nrf2 activity, as assessed by reporter assays (Figure 5C). These findings suggest that baseline Nrf2 activity is, at least in part, supported by endogenous Perm1. Importantly, Perm1 and H_2_O_2_ exert additive effects on Keap1 oxidation (Figure 6C), indicating that Perm1 is not the sole regulator of this pathway and that Perm1 and oxidative stress act as parallel inputs for Keap1 oxidation (Figure 7F). Perm1 may function to enhance the antioxidant defense by amplifying Nrf2 activation in tissues with high metabolic demand.

### Perm1 promotes Keap1 oxidation.

Our results show that Perm1 physically interacts with Keap1 and prevents the interaction between Nrf2 and Keap1 without affecting Keap1-Cul3 interaction. On the other hand, downregulation of Perm1 promoted association between Keap1 and Nrf2. Thus, it is likely that physical interaction between Perm1 and Keap1 inhibits the Keap1-Nrf2 interaction, thereby stabilizing Nrf2. Keap1 has highly reactive cysteine residues, whose oxidation leads to intra- or intermolecular disulfide bond formation, disruption of zinc coordination, and possibly other forms of modification, globally affecting the protein structure and leading to stabilization of Nrf2 (12). We showed that overexpression of Perm1 potently induced cysteine oxidation of Keap1. Perm1 increased inter- or intramolecular disulfide bond formation of Keap1 at Cys151, as evidenced by a characteristic 2ME-sensitive band shift of Keap1. We also found that Perm1-induced upregulation of Nrf2 was inhibited in cultured cardiomyocytes in the presence of Keap1 (C151S). These results suggest that Perm1 induces oxidation of Keap1, including at Cys151, which in turn mediates stabilization of Nrf2. It should be noted that, however, Keap1 is a cysteine-rich protein and Perm1 likely oxidizes multiple cysteine residues in Keap1, with Cys151 being an important regulatory site but not the sole target. Thus, it is currently unknown whether Cys151 is directly oxidized by Perm1, such as through intermolecular disulfide bond formation. Disulfide bond formation at Cys151 may take place secondary to global conformational changes caused by oxidation of multiple cysteine residues. In fact, oxidation Keap1 at Cys151 is commonly observed in response to electrophiles (34). Future studies involving comprehensive cysteine mapping by mass spectrometry will be required to identify the specific cysteine residues targeted by Perm1 and to determine their individual contributions to Keap1 regulation and Nrf2 activation.

### Critical cysteine residues in Perm1.

We show that Perm1 has highly reactive cysteine residues. This property causes Perm1 to be oxidized by stress and, in turn, reduced again by another protein with reactive cysteines. Both human and mouse Perm1 oxidize Keap1, and Cys746 in Perm1 is evolutionarily conserved not only in mammals but also in Drosophila and zebrafish. Our results suggest that Cys121, Cys746, or other cysteines of Perm1 are involved in Perm1-induced oxidation of Keap1. The molecular mechanism by which these cysteines mediate Keap1 oxidation remains to be elucidated. One possible mechanism is a thiol-disulfide exchange reaction (3, 39). We recently showed that a protein with reactive cysteines, namely Trx1, is reduced by Atg7, which also possesses highly reactive cysteines, when they interact with one another (32). Thus, Cys121 and Cys746 of Perm1 may participate in either intramolecular disulfide bond formation within Perm1 or in mixed disulfide formation with Keap1 or other redox-sensitive proteins. Consistent with this idea, Perm1 C121S and C746S mutants significantly attenuated Nrf2 activation, suggesting that these residues are required for its redox function (Figure 7D). These mutants may retain binding to Keap1 but lack the ability to promote its oxidation, thereby stabilizing Keap1 in a reduced state and exerting a dominant-negative effect. Although intermolecular disulfide bond formation between Perm1 and Keap1 is a plausible mechanism, direct evidence remains lacking. In principle, such interactions could be detected by mass spectrometry. However, standard proteomic workflows, including enzymatic digestion of Perm1, did not readily yield peptides of suitable sizes for reliable detection, limiting our ability to directly identify such crosslinked species at present. Further studies, including optimized mass spectrometry approaches and the use of cysteine-trapping mutants of Perm1 and Keap1, will be required to directly demonstrate intermolecular disulfide bond formation, similar to the Trx1 trapping mutant (33).

### Perm1 acts as a unique activator of Nrf2.

The role of cysteine oxidation of Keap1 in Nrf2 activation has been investigated in cancer cells, using electrophiles as inducers of cysteine oxidation (12, 40). These small molecules induce cysteine oxidation of Keap1 and protect Nrf2 from Cul3-mediated ubiquitination, but they fail to release Nrf2 from Keap1 (41). In contrast, an endogenous protein p62 interacts with the Kelch domain of Keap1 to induce the release of Nrf2, this occurs without cysteine oxidation (42). H_2_O_2_ also induces oxidation (intramolecular disulfide formation) of Keap1 (34). Importantly, Perm1 induced Keap1 oxidation in vitro more potently than H_2_O_2_, and its effect was additive to the effect of H_2_O_2_ (Figure 6C). Unlike electrophiles, Perm1 promotes both Keap1 oxidation and Nrf2 release from Keap1. Thus, we speculate that Perm1 promotes dissociation of Nrf2 from Keap1 through cysteine oxidation via a mechanism distinct from those mediated by electrophiles, H_2_O_2_, or p62.

### Potential additional mechanisms of Nrf2 regulation by Perm1.

The activity of Nrf2 is also regulated by nuclear translocation through posttranslational modification of Nrf2, including phosphorylation (43). Perm1 controls the activities of signaling molecules involved in Nrf2 phosphorylation, such as p38-MAPK (7) and CaMKII (44), through unknown mechanisms. Perm1 interacts with PGC-1 (20), which in turn may promote its function as a coactivator for Nrf2 (45). Thus, these mechanisms may also contribute to the positive regulation of Nrf2 by Perm1. Further investigation is required to clarify these issues.

### Redox cycle of Perm1.

Although a previous work raised the possibility that Keap1 undergoes thiol disulfide exchange reactions with other proteins (32), the identity of Keap1’s partner is unknown. Inhibition of Trx reductase 1 (TrxR1) promotes Keap1 oxidation (37). Here we show that the Trx1-trapping mutant binds to not only Keap1 but also Perm1, supporting a role for Trx1 in the redox regulation of both proteins (Figure 6D). Using unbiased mass spectrometry–based screening of Perm1-bound proteins in cardiomyocytes, Trx1 has been identified as a Perm1 binding protein (21). Trx1 is classically known to reduce proteins containing oxidized cysteine residues (3, 33, 46); however, it can also promote the oxidation of proteins with reduced thiols, such as Atg7, through thiol-disulfide exchange reactions (32). These results suggest that, like Trx1, once oxidized during IR, Perm1 may be reduced by redox-sensitive, cysteine-containing proteins such as Keap1.

### Integration of metabolic and redox functions of Perm1.

Previous studies have shown that Perm1 promotes transcription of genes involved in fatty acid oxidation and mitochondrial oxidative phosphorylation through its interaction with PGC-1α, PPARα, and ERRα in the nucleus (18, 20, 21). Consistent with this, Perm1 protein was downregulated in the heart during IR, accompanied by downregulation of *Ppars* and *Esrrs* (Supplemental Figure 3A). In addition to its role in transcriptional regulation, Perm1 also contributes to mitochondrial organization and function through its association with the MICOS-MIB complex, supporting energy metabolism in a transcription-independent manner (11). Since oxidative phosphorylation and fatty acid oxidation generally promote the production of ROS (23), downregulation of Perm1 and its downstream targets during IR could be adaptive. In fact, our results show that the loss of ERR function was protective, whereas a gain of ERR function was detrimental during IR injury in the heart (Supplemental Figure 3, B and C). Notably, the enhancement of IR injury in *Perm1*-cKO mice was fully rescued when Nrf2 was upregulated through AAV-mediated gene transfer or in the presence of ML334, a small molecule that stabilizes Nrf2 by promoting its release from Keap1 (28), indicating that the impairment of Nrf2 upregulation during IR plays a critical role in mediating the exacerbation of IR injury in these mice. The consistent rescue observed with both genetic and pharmacological approaches strongly supports the conclusion that impaired Nrf2 activation is a key mechanism underlying the exacerbated IR injury in these mice. These results suggest that the salutary mechanism mediated by the Perm1-induced Nrf2-dependent antioxidant pathway predominates over other potential adverse metabolic effects, namely the stimulation of mitochondrial oxidative phosphorylation, during myocardial IR. Thus, our finding is distinct from previous studies in which the protective effect of Perm1 was attributed almost exclusively to Perm1-mediated upregulation of ERRα, PPARα, and PGC-1α (18).

### Limitations of this study.

Several limitations should be acknowledged. First, although our findings demonstrate a protective role of Perm1 in murine models of IR, the relevance of these findings to humans remains to be determined. Second, because Perm1 rescue was achieved using AAV9-mediated gene transfer prior to ischemia, the possibility that Perm1 may exert only pre-emptive effects cannot be excluded. Since clinical interventions are typically applied at the time of reperfusion, it remains to be determined whether restoring Perm1 expression during reperfusion can mitigate IR injury. Development of small molecules that mimic Perm1 function may facilitate the translation of these findings. Third, while our data support a role for Perm1 in regulating the Keap1/Nrf2 pathway through redox-dependent mechanisms, we cannot exclude the possibility that Perm1 may also influence Nrf2 signaling through Keap1-independent or nuclear mechanisms. Similar to its reported role in PPAR/ERR activation, Perm1 may also modulate Nrf2-dependent transcriptional programs in the nucleus. Furthermore, the specificity of Perm1-mediated cysteine oxidation remains unclear. It is not known whether Perm1 selectively targets Keap1 or more broadly modulates cysteine oxidation of multiple proteins, nor whether additional substrates contribute to its biological effects. Finally, although we confirmed key findings using *Perm1*-cKO mice, the majority of mechanistic analyses were performed in systemic knockout settings. More comprehensive characterization of tissue-specific roles of Perm1 will be required in future studies.

In conclusion, endogenous Perm1 plays a salutary role in the heart during IR by mediating upregulation of antioxidants through stimulation of Nrf2. Perm1 oxidizes Keap1 via evolutionarily conserved cysteine residues, including Cys746. Although the actions of Perm1 could be mediated primarily through PGC-1α and ERRα, our results suggest that the salutary effect of Perm1 during IR is mediated through the Keap1-Nrf2 pathway. The identification of Perm1 as an endogenous regulator of Keap1 oxidation provides a conceptual advance and suggests a potentially more physiologically relevant strategy for modulating the Keap1/Nrf2 pathway. Further investigation regarding the molecular mechanism through which Perm1 interaction with Keap1 interferes with Keap1-Nrf2 interaction may lead to the development of therapeutic strategies to stabilize Nrf2 and activate antioxidant mechanisms for cell protection.

## Methods

### Sex as a biological variable.

Both male and female mice were included in this study. Since potential sex differences in this model were unknown, using both sexes enabled assessment of possible sex-dependent effects. This approach also helped reduce overall animal use while ensuring representative biological data. All experiments were conducted with randomization, allocation concealment, and in a blinded manner.

### Generation of Perm1 homozygous KO mice.

*Perm1* systemic homozygous KO mice (C57BL/6 background) were generated with a CRISPR/Cas9 system in the Genome Editing Shared Resource of Rutgers Cancer Institute of New Jersey (20).

### Generation of cardiac-specific Perm1-KO mice.

*Perm1*-cKO mice were generated using CRISPR-Cas9 by insertion the loxP sites flanking exon 2 of the *Perm1* gene. Cardiomyocyte-specific deletion was achieved by crossing these mice with *Myh6-Cre* mice.

### IR model and assessment.

Myocardial IR was induced by transient ligation of the left anterior descending coronary artery (LAD) followed by reperfusion (47). ML334 was administered i.p. 24 hours prior to IR. Infarct size was assessed by TTC staining.

### Primary cultures of NRVMs.

The method used to prepare primary cultures of NRVMs has been described (23).

### Immunoblot analyses.

Heart homogenates were prepared in RIPA lysis buffer containing 50 mmol/L Tris (pH 7.5), 150 mmol/L NaCl, 1% IGEPAL CA-630 (Sigma Aldrich), 0.1% SDS, 0.5% deoxycholic acid, 10 mmol/L Na_4_P_2_O_7_, 5 mmol/L EDTA, 1:100 diluted Protease Inhibitor Cocktail (Sigma Aldrich, P8340-5ML), and 1:100 diluted Phosphatase Inhibitor Cocktail (Sigma Aldrich, P0044-5ML). Protein amounts were measured by BCA quantification and 20-30 mg were subjected to 10%–15% SDS-PAGE. Proteins were transferred to a 0.2 mm PVDF membrane and immunoblots were probed with the primary antibodies.

### Adenovirus vectors.

The method used to generate adenovirus vectors has been described (8). The Perm1 sequence was cloned from mouse *Perm1* cDNA. *Perm1* cDNA was cloned into the pDC316 shuttle vector plasmid to generate pDC316-Perm1. A C-terminal Flag tag was inserted into pDC316-Perm1 to generate pDC316-Perm1-Flag. After sequencing, HEK293 cells were used for adenovirus packaging and amplification. Adenovirus harboring beta-galactosidase (Ad-LacZ) was used as a control.

### siRNA for Perm1.

siRNAs were transfected using Lipofectamine RNAiMax (Invitrogen).

### Ischemia and reperfusion in cell cultures.

In order to mimic the conditions of in vivo myocardial IR in cultured NRVMs in vitro, sIR was performed using a hypoxic chamber, as described previously (19).

### BIAM pull down.

Free thiol groups in proteins were labeled using BIAM, which irreversibly reacts with reduced cysteine residues. Biotinylated proteins were then captured using avidin-based pull-down assays and subjected to immunoblot analysis.

### Generation of recombinant Perm1.

The BL21 *E.coli* strain was transformed with pCold-GST-Perm1, a bacterial expression plasmid vector. The *E.coli* was grown in 3 mL LB medium overnight at 37°C and then transferred to 250 mL LB medium. After overnight culture with 1 μmol/L isopropyl β-D-1-thiogalactopyranoside (IPTG) at 15°C, the *E.coli* was lysed in lysis buffer (1% Triton X-100, 1 mmol/L dithiothreitol [DTT], PBS) with sonication. The lysate was incubated with 0.5 mL GSH-sepharose 4B (GE Healthcare) for 1 hour at 4°C. The sepharose was washed 3 times with 1 mL of lysis buffer. The recombinant GST-Perm1 was cleaved with a precision protease.

### Statistics.

All values in graphs are expressed as the mean ± SEM. Normality was tested with the Shapiro-Wilk normality test. If the data exhibited a normal distribution, pairwise testing was performed with a 2-tailed Student’s *t* test, and multiple-group comparisons were performed by 1-way ANOVA, followed by Tukey post hoc test. A *P* value of less than 0.05 was considered statistically significant. A priori power calculations were performed based on data from published studies (8, 46, 47) and pilot experiments. The effect size in this study was 1–5 with an α = 0.05 and power = 0.80. Microsoft Excel 2016 was used for 2-tailed Student’s *t* tests, and GraphPad Prism 9 was used for 1-way ANOVA and Tukey post hoc tests. Dot plots were used. All quantified Western blot and cell viability data are shown as values relative to control.

### Study approval.

All animal procedures were reviewed and approved by the IACUC of Rutgers Biomedical and Health Sciences, Newark, New Jersey, USA.

### Data availability.

Values for all data points in graphs are reported in the Supporting Data Values file.

## Author Contributions

SO and CYH designed the study, performed most of the experiments, analyzed data, and interpreted results. MM maintained animal lines and performed TTC staining. AST performed in vitro redox reactions. YN performed PLA assays. RM oversaw the international sample and data management process. SV and YM performed Western blot analyses. GY and PR generated genetic animal models. YT generated adenovirus vectors. PZ performed animal surgery. JS oversaw the entire study and maintains experimental resources and environment. All authors reviewed and approved the manuscript for publication.

## Conflict of interest

The authors have declared that no conflict of interest exists.

## Funding support

This work is the result of NIH funding, in whole or in part, and is subject to the NIH Public Access Policy. Through acceptance of this federal funding, the NIH has been given a right to make the work publicly available in PubMed Central.

- This work was supported in part by the Taiwan Association of Cardiovascular Surgery Research (1070720001 to CYH), the New Jersey Health Foundation (PC-56-16 to SO), the American Heart Association (19TPA34850170 to SO and 25TPA1481361 to JS), U.S. Public Health Service grants (HL091469, HL112330, HL144626, and HL179656 to JS).

## Supplementary Material

Supplemental data

Unedited blot and gel images

Supporting data values

## Version 1 05/12/2026

In-Press Preview

## Version 2 07/22/2026

Electronic publication
