# Supplementary material for: Perm1 enhances Nrf2-driven antioxidant defense through Keap1 oxidation during myocardial ischemia/reperfusion injury
Source: JCI Insight. 2026 May 12;11(14):e204132. doi: 10.1172/jci.insight.204132 (PMC13461155; doi:10.1172/jci.insight.204132)
Supplement: Supplementary file 1 [file jciinsight-11-204132-s359.pdf]

Supplemental Material

Perm1 enhances Nrf2-driven antioxidant defense through Keap1 oxidation during myocardial ischemia/reperfusion injury

Running title: ***Perm1 enhances antioxidant defense***

Shin-ichi Oka[1], Chun-Yang Huang[1,2,3], Masato Matsushita[1], Allen Sam Titus[1], Yasuki Nakada[1], Risa Mukai[1], Samta Veera[1], Youssef Mourad[1], Ghassan Yehia[4], Peter Romanienko[4], Yimin Tian[1], Peiyong Zhai[1], and Junichi Sadoshima[1,5]

Affiliation:

[1]Department of Cell Biology and Molecular Medicine, Rutgers New Jersey Medical School, Newark, NJ 07103.

[2]Division of Cardiovascular Surgery, Department of Surgery, Taipei Veterans General Hospital, Taipei, Taiwan.

[3]Department of Medicine, School of Medicine, National Yang-Ming Chiao-Tung University, Taipei, Taiwan.

[4]Genome Editing Shared Resource, Rutgers Cancer Institute, New Brunswick, NJ 08901

SO and CYH contributed equally to this study.

[5]Correspondence:
Junichi Sadoshima, MD PhD
Department of Cell Biology and Molecular Medicine,
Rutgers New Jersey Medical School
185 South Orange Ave., MSB G609
Newark, NJ 07103
Tel: (973)972-8619
Fax: (973)972-7489
Email: sadoshju@njms.rutgers.edu

Key words: Perm1, ischemia/reperfusion, Nrf2, antioxidants, heart

Supplemental Methods

Generation of *Perm1* homozygous knockout (KO) mice

The systemic *Perm1* KO mouse was generated by co-injecting two CRISPR gRNAs (MilliporeSigma) with Cas9 protein (MilliporeSigma) into C57BL/6J zygotes to generate a deletion in the coding sequence (Online Figure 1). The gRNAs targeted the sequences 5'-TGATGAGTGTGGCCTCTTGCAGG-3' and 5'-GAAGCTGCCATGGTGGATACAGG-3' (PAM underlined). Founder mice were genotyped to detect the approximately 1.3 kb deletion. Primers Perm1A 5'-ATCCTGGATTTGTGAAGACCCTGC-3' and Perm1D 5'-ACGGCTTCCTGCTGCTGTCCTG-3' were used to amplify the alleles. Wild-type alleles in the founders were 1716 bp, whereas deletion alleles were approximately 384 bp, depending on the precise deletion. Founder 80, which was used for subsequent experiments, had a deletion of 1346 bp, which encompassed almost 2/3 of the coding sequence of exon 2.

Generation of cardiac-specific *Perm1* knockout (KO) mice

Perm1 conditional KO mice were generated using CRISPR-Cas9. To insert the loxP sites, Cas9:sgRNA ribonucleoprotein complexes (RNPs) were microinjected into C57BL/6J embryos along with ssODNs that contained homology arms flanking loxP sequences. The 5' loxP sgRNA spacer sequence was TTTCTGTGGTTAGCTGCCGG (Millipore-Sigma) and the 5' loxP oligo donor (IDT DNA) sequence was 5'-ATCAGTTCTCTATGACTGTCATTCCTAGCCTCCCCAGCTACCACCACCGA**ATAACTTCGTATAATGTATGCTATACGAAGTTAT**GCAGCTAACCACAGAAAAGAATCTATAGTGTCCAGCAGGTGACCAGCCTTC -3' (loxP in bold). For the 3' loxP, the sgRNA spacer sequence was GCCAGGCCCTTAAAAGCTAG and the 3' loxP donor oligo sequence was 5'-GTTTGGGCCTTGTCACTGACCACTGTCCTAGATGCCATCAGGGACTTTCCTCT**ATAACTTCGTATAGCATACATTATACGAAGTTAT**TCGCTTTTAAGGGCCTGGCACTAGAGGCCCACTCACACTCAGGCCAT -3' (loxP in bold). Six founders were determined to have both loxP sites correctly inserted, three were bred for transmission, and two of the bred founders transmitted the loxP sites to their progeny. *Myh6*-Cre-mediated

recombination of the loxP sites results in removal of exon 2, which contains most of the coding sequence, in a cardiomyocyte-specific manner. PCR primers Perm1J 5'-ACTCCTGAGTAGGCTCTAGCCAG -3' and Perm1K 5'-GTTTGGATGGCGTTCCCTCACATC -3' were used to analyze the 5' loxP insertion and primers Perm1L 5'- CAGCGGAACATCTTAGCTTAACAG -3' and Perm1M 5'-AGTTGAGTCAGAACAACTAGGGAG -3' were used to analyze the 3' loxP insertion. Determination of incorporation of both loxP sites on the same allele was carried out by amplifying founder genomic DNA with primers Perm1J and Perm1M and using Sanger sequencing to confirm the loxP sequences on the same allele.

IR in mice

Mice were housed in a temperature-controlled environment with 12-hour light/dark cycles, where they received food and water ad libitum. ML334 (0.01 mg/100 μL) dissolved in PBS was intraperitoneally injected 24 hours before IR at 5 μL per gram of body weight. Mice were anesthetized by intraperitoneal injection of pentobarbital sodium (65 mg/kg) and intubated. A rodent ventilator (TYPE 845, MiniVent, Harvard Apparatus Inc) was used during the surgical procedure. The mice were kept warm using a heat pad. Rectal temperature was maintained at 37°C. After sterilization, a horizontal incision was created in the left chest, and the wound was advanced to the fourth intercostal space. Ischemia was achieved by ligating the anterior descending branch of the left coronary artery (LAD) using an 8-0 prolene suture with silicon tubing (1 mm outer diameter) placed on top of the coronary artery, 2 mm below the border between the left atrium and left ventricle (LV). Ischemia was confirmed by ECG change (ST elevation). After occlusion for 30 minutes, the silicon tubing was removed to achieve reperfusion, and the wound was closed. The infarction area was evaluated by TTC staining 24 hours postoperatively, as described below. A control group of mice matched for age and gender were subjected to the same surgical procedure without the LAD ligation and designated as sham-operated mice.

Assessment of myocardial infarction

Twenty-four hours postoperatively, the mice were anesthetized and intubated. The chest was opened, along with the middle of the sternum. The original stitch used to create ischemia was retightened. KCl was injected to arrest the heartbeat at the diastolic phase. The ascending aorta was cannulated with saline perfusion to wash out blood. To demarcate the area at risk (AAR) of ischemia, Alcian blue dye (0.5%) was perfused into the ascending aorta and coronary arteries. After the heart was harvested, the LV was sliced into six pieces with 1 mm thickness. The six slices were incubated with a 1% fresh triphenyltetrazolium chloride (TTC) solution at 37°C for 10-15 minutes. The slices were transferred to a 10% formalin solution for fixation. The infarct area (white), the AAR (brick-red), and total LV area were measured on both sides of the section using ImageJ software (NIH, USA). For infarct size and AAR, the percentages for each slice were multiplied by the slice weight and combined. AAR/LV and infarct area/AAR are shown as percentages.

Primary cultures of neonatal rat ventricular myocytes

Primary cultures of NRVMs were prepared from 1 day-old Hsd:WI rats (Envigo). A cardiomyocyte-rich fraction was obtained by centrifugation through a discontinuous Percoll gradient. Cells were cultured in complete medium for 1-2 days. The complete medium contained Dulbecco's modified Eagle's medium (DMEM)/F-12 supplemented with 5% horse serum, 4 μg/mL transferrin, 0.7 ng/mL sodium selenite (Life Technologies, Inc.), 2 g/L bovine serum albumin (fraction V), 3 mmol/L pyruvic acid, 15 mmol/L Hepes, 100 μmol/L ascorbic acid, 100 μg/L ampicillin, 5 μg/mL linoleic acid, and 100 μmol/L 5-bromo-2'-deoxyuridine (Sigma).

Antibodies

The following primary antibodies were used for immunoblots: Anti-Perm1 (Sigma Aldrich, HPA031711), Anti-Lamin A/C (Cell Signaling Technology, 4777S), Anti-Dityrosine (StressMarq, SMC0521D), Anti-Nrf2 (Novus, MBP1-32822, Abcam ab62352), Anti-Keap1 (Proteintech, I0503-2-AP), Anti-Catalase (Abcam, ab1877), Anti-SOD2 (Cell Signaling Technology, 13141S), Anti-HO1 (Proteintech, 27782-1-AP), Anti-NQO1 (Invitrogen, MA1-16672), Anti-Thioredoxin (Cell Signaling Technology, 2929S), Anti-

Prdx1 (Abcam, ab41906), Anti-Prdx1-SO3H (Abcam, ab16830), Anti-4HNE (Abcam, ab46545), Anti-Cul3 (Cell Signaling Technology, 10450S), Anti-GAPDH (Cell Signaling Technology, 2118C), and Anti-Tubulin (Cell Signaling Technology, 3873S). Secondary antibodies used were Anti-Rabbit IgG, HRP-linked (Cell Signaling Technology, 7074S) and Anti-Mouse IgG, HRP-linked (Cell Signaling Technology, 7076S). In all immunoblot images shown in figures, individual lanes correspond to individual mice. Molecular weights (MW) associated with the bands are shown. The signal intensities of the Western blots were quantified using ImageJ software and then normalized with corresponding loading controls (Tubulin). Phosphorylated protein was normalized with the total amount of the protein without modification.

siRNA for Perm1

Small interfering RNA (siRNA) for rat Perm1 (1027417) and control siRNA (1022076) were obtained from Qiagen. Four and a half μL of Lipofectamine RNAiMax was diluted with 125 μL Opti-MEM. Two μL of 20 μmol/L siRNA was diluted with 15 μL Opti-MEM. Diluted Lipofectamine RNAiMax and siRNA were then mixed and incubated at room temperature for 20 minutes. The mixture was added to NRVMs plated on a 3.5 cm dish with 2 mL DMEM/F21 medium. The cells were used 1-2 days after transfection.

Cell viability and LDH activity assay

Cell viability was measured using the CellTiter-Blue® Cell Viability Assay (Promega), and release of LDH activity from the cytoplasm of intact cells was measured using the CytoTox 96® Non-Radioactive Cytotoxicity Assay (Promega) according to the manufacturer's protocol, as described previously (1).

Ischemia and reperfusion in cell cultures

The culture medium was replaced with an ischemic buffer containing 137 mmol/L NaCl, 0.49 mmol/L $MgCl_2$, 0.9 mmol/L $CaCl_2$, 4 mmol/L HEPES, 10 mmol/L deoxyglucose, 0.75 mmol/L sodium dithionate, 12 mmol/L KCl, and 20 mmol/L lactate, pH=6.5, which induces nutrient deprivation and acidosis (2). Cells were then incubated in a small humidified hypoxic chamber containing 5% CO_2 and 95% N_2 and placed in an incubator

at 37°C. After 30 minutes of ischemia, the cells were removed from the hypoxia chamber. The ischemia buffer was then replaced with the original medium. After 1-2 hours, the cells were harvested for experiments. In some experiments, cultured NRVMs were subjected to sIR in the presence of either ML334 (Sigma Aldrich, 50 μmol/L) or vehicle. Cells were subjected to 16 hours of pretreatment before sIR.

Immunoprecipitation

After hearts were harvested, the tissue was homogenized in lysis buffer containing 150 mmol/L NaCl, 50 mmol/L Tris HCl (pH 7.4), 1% Triton X-100, and 1 mmol/L EDTA. Primary antibodies, including anti-Perm1, Keap1, Nrf2 or control IgG antibody, were used for immunoprecipitation. Protein A/G-Agarose beads (Santa Cruz, sc2003) were added for the pulldown. Flag-Perm1 was immunoprecipitated from cultured NRVMs transduced with Flag-Perm1 adenovirus vector using anti-Flag-agarose beads (Sigma A5496). The beads were washed with lysis buffer three times.

GST-pulldown assays

Recombinant GST-Perm1 proteins were incubated with or without recombinant Flag-Keap1 in lysis buffer containing 50 mmol/L Tris-HCl pH 7.4, 150 mmol/L NaCl, and 0.1% Triton-X 100 with rotation for 2 hours at 4℃, followed by pull down with anti-Flag agarose. After washing seven times with lysis buffer, proteins were eluted with 2× SDS sample buffer.

Biotin-Iodoacetamide (BIAM) pull down

NRVMs were lysed with lysis buffer [50 mmol/L Tris pH 7, 150 mmol/L NaCl, 0.5% NP-40 Substitute, Protease Inhibitor Cocktail (Sigma)] containing 200 μM BIAM. BIAM stock solution (20 mM) was freshly prepared in DMSO immediately prior to use and added to the lysis buffer to achieve a final concentration of 200 μM. The lysates were clarified by centrifugation at 12,000 × g for 10 minutes at 4°C. The supernatants were incubated with Avidin resin (Promega, V201A) for 2 hours at 4°C with gentle rotation. The resin was then washed three times with 1 mL of lysis buffer without BIAM to remove non-specifically bound proteins. The samples were eluted using 2× SDS

sample buffer (120 mmol/L Tris-HCl pH 6.8, 20% Glycerol, 4% SDS, 0.02% Bromophenol Blue, 10% β-mercaptoethanol) and subjected to immunoblot analyses.

In vitro redox reaction

Recombinant Perm1 protein was oxidized by incubation in reaction buffer containing 100 μmol/L hydrogen peroxide (H_2O_2) at room temperature for 1 hour. Residual H_2O_2 was subsequently removed by centrifugation using a centrifugal filter unit (Amicon Ultra-0.5, Millipore, UFC500396). Recombinant His-tagged Keap1 protein (BPS Bioscience, 101132) was reduced in a reaction buffer composed of 100 mmol/L Tris-HCl pH 6.8 and 150 mmol/L NaCl, supplemented with 1 mmol/L dithiothreitol (DTT), for 1 hour. The reduced Keap1 was then incubated with Ni-NTA resin (Thermo Scientific, #88221) and washed twice with 1 mL of the reaction buffer to remove residual DTT. Oxidized Perm1 was incubated with immobilized Keap1 for 1 hour. Free thiol groups were subsequently labeled by adding 1 mmol/L biotinylated iodoacetamide (BIAM) and incubating for 1 hour at room temperature. The labeling reaction was terminated by adding sample buffer containing 320 mmol/L β-mercaptoethanol. Samples were then analyzed by SDS-PAGE followed by Western blotting. BIAM-labeled proteins were detected using horseradish peroxidase (HRP)-conjugated streptavidin (Thermo Scientific, 21130).

Luciferase reporter system

Luciferase reporter assays were conducted with the ARE Reporter Kit designed to evaluate the activity of the Nrf2 antioxidant pathway (BPS, Bioscience, 60514). NRVMs were plated on 96-well dishes. One μL of an ARE luciferase plasmid premixed with a Renilla luciferase vector plasmid (60 ng DNA/μL), which served as an internal control, was transfected using 0.35 μL Lipofectamine 2000 (Invitrogen, 11668-019) in Opti-MEM medium for 24 hours at 37°C. Myocytes were then subjected to sIR as described above. After harvest with the Dual-Luciferase Assay System (BPS, Bioscience, 60683), reporter activity in the samples was measured with a luminometer. The data were normalized with the Renilla luciferase value for each sample.

Tissue subcellular protein fractionation

Subcellular fractionation of mouse heart homogenates was conducted as described previously (1), using the Subcellular Protein Fractionation Kit (Thermo Scientific, 87790). After the heart tissue was harvested, ice-cold CEB (Cytoplasmic extract buffer) containing protease inhibitors was added, followed by homogenization. The sample was centrifuged at 500 x g for 5 minutes and the supernatant was transferred to a new tube as the cytoplasmic extract. Ice-cold MEB (Membranous extract buffer) was then added to the pellet and the sample was centrifuged at 3000 x g for 5 minutes. The supernatant was transferred to a new tube as the membranous extract. The pellet was then mixed with ice-cold NEB (Nucleus extract buffer) and the sample was centrifuged at 5000 x g for 5 minutes. The supernatant was transferred to a new tube as the nuclear extract. The cytoplasmic and nuclear extracts were then used for further experiments in this study.

GSH and GSSG measurements

GSH and GSSG were measured using the EnzyChrom™ GSH/GSSG Assay Kit (EGTT-100, Bioassay Systems, Hayward, CA) according to the manufacturer's protocol (3).

AAV9 injection

AAV9-*cTNT-Perm1* (mouse cDNA) was generated at VectorBuilder (Chicago, IL). We used AAV9-*cTNT-GFP* as control. Purified AAV9 vectors (3 x 10^{11} particles) were injected into the extrajugular vein (4). After 4 weeks, Perm1 overexpression in the heart was confirmed by Western blots.

Adenovirus injection

Adenoviral vectors encoding wild-type Perm1, C121S or C746S Perm1 mutants, or β-galactosidase were injected intramuscularly using a syringe with a 30-gauge needle into the LV free wall (1 x 10^9 optical particle unit/30 μL). IR surgery was performed 3 days after injection.

Proximity ligation assay (PLA)

PLA technology allows the detection of interactions between endogenous proteins, based on protein proximity. PLA was carried out with Duolink® In Situ Detection Reagents (Sigma Aldrich). In brief, myocytes were washed, blocked, and then incubated with anti-Keap1 and anti-Nrf2 primary antibodies for 2 hours at room temperature. After washing, myocytes were incubated with Duolink PLA MINUS and PLUS probes for 1 hour at 37°C. A Duolink In Situ Detection Kit was used for ligation and amplification. DAPI was used to stain the nucleus. The images were captured using a fluorescent microscope, and red spots indicate the interaction between Keap1 and Nrf2.

Quantitative RT-PCR

Total RNA was extracted from mouse hearts using TRIzol (Invitrogen). Total RNA was converted to cDNA using PrimeScript RT Master Mix (Takara). The following oligonucleotide primers were used in this study:

Cat, sense (5’- TACCCCAACAGCTTCAGCGCAC-3’) and antisense (5’-CAGGCGTTTCCTCTCCTCCTC-3’); *Sod2*, sense (5’-GTGTGAATTGCTCTTGATTGAACATTTTC-3’) and antisense (5’-CTAACTGCAGTAGAACAGGATTACAG-3’); *Ho1*, sense (5’-CCTCACAGATGGCGTCACTT-3’) and antisense (5’- GCTGATCTGGGGTTTCCCTC-3’); *Nqo1*, sense (5’- AGCCAATCAGCGTTCGGTAT-3’) and antisense (5’-GCCTCCTTCATGGCGTAGTT-3’); *Trx1*, sense (5’-ATCGAGAGCAAGGAAGCTTTTCAGG-3’) and antisense (5’-TGCAGCAACATCCTGGCAGTCAT-3’); *Erra*, sense (5’-GCTGAAAGCTCTGGCCCTTGCC-3’) and antisense (5’-CGGAGGAGTGGCAGCGTAAGCA-3’); *Errb*, sense (5’-AAGAGTTTATGATCCTCAAGGCCCTG-3’) and antisense (5’-TCCGCGGCTCCTCGTGGC-3’); *Errg*, sense (5’-CAAGAGCATGAAGCTAGAGAAGGAAG-3’) and antisense (5’-GGCCTCATGTAACACATCCTGAAG-3’); *Ppara*, sense (5’-CTGGTCACGGAGCATGCGCA-3’) and antisense (5’-TCTGTGCAAATCCCTGCTCTCCT-3’); *Pparb*, sense (5’-

TTGCTGCACCCCCTGCTCCA-3’) and antisense (5’-GGTCCTCTGAACAGTCCGTGG-3’); *Pparg*, sense (5’-AGCTACTGCATGTGATCAAGAAGAC-3’) and antisense (5’-CCCTCAAAATAATAGTGCAATCAATAGAAGG-3’); *B-actin*, sense (5’-AAGACCTCTATGCCAACACAGTGC-3’) and antisense (5’-CACTTGCGGTGCACGATGGAG-3’); Control *RPS15*, sense (5’-TTCGCAAGTTCACCTACC-3’) and antisense (5’- CGGGCCGGCCATGCTTTA -3’). Data were normalized with the RT-PCR result for *Gapdh*. Quantitative RT-PCR was performed on the CFX 96 RealTime PCR Detection System (Bio-Rad). The Ct value determined using CFX Manager Software (version 2.0, Bio-Rad) for all samples was normalized to *Gapdh* and the relative fold change was computed by the comparative Ct (ΔΔCt) method.

Supplementary References

1. Nah J, Zhai P, Huang CY, Fernandez AF, Mareedu S, Levine B, et al. Upregulation of Rubicon promotes autosis during myocardial ischemia/reperfusion injury. *J Clin Invest.* 2020;130(6):2978-91.

2. Si J, Wang N, Wang H, Xie J, Yang J, Yi H, et al. HIF-1alpha signaling activation by post-ischemia treatment with astragaloside IV attenuates myocardial ischemia-reperfusion injury. *PLoS One.* 2014;9(9):e107832.

3. Aasum E, Belke DD, Severson DL, Riemersma RA, Cooper M, Andreassen M, et al. Cardiac function and metabolism in Type 2 diabetic mice after treatment with BM 17.0744, a novel PPAR-alpha activator. *Am J Physiol Heart Circ Physiol.* 2002;283(3):H949-57.

4. Shirakabe A, Zhai P, Ikeda Y, Saito T, Maejima Y, Hsu CP, et al. Drp1-Dependent Mitochondrial Autophagy Plays a Protective Role Against Pressure Overload-Induced Mitochondrial Dysfunction and Heart Failure. *Circulation.* 2016;133(13):1249-63.

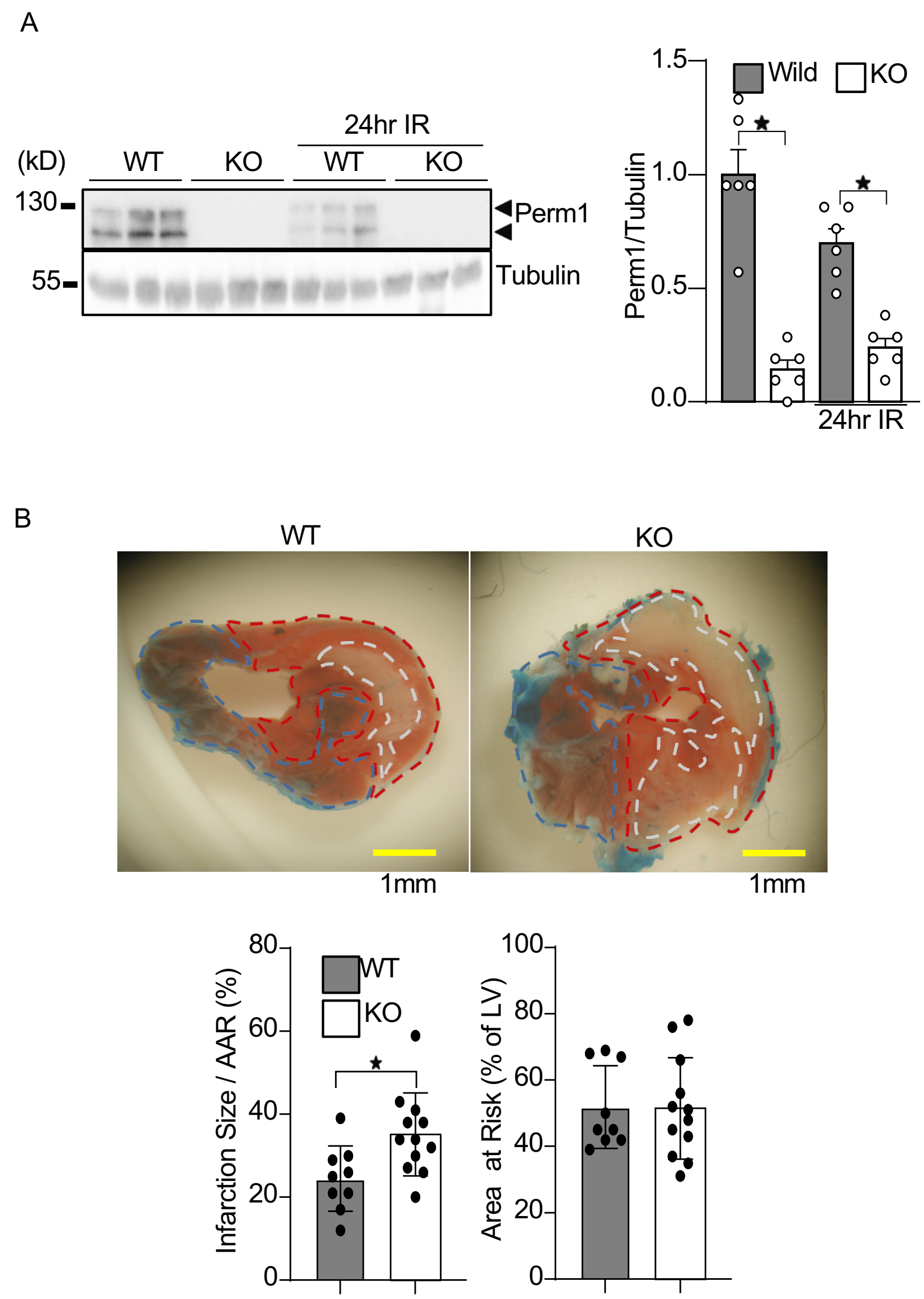

Figure S1. (A) Loss of Perm1 in *Perm1* KO mice was verified with Western blot analyses. (B) Loss of Perm1 exacerbated IR injury. Alcian blue and triphenyltetrazolium chloride (TTC) staining was performed 24 hours after IR in *Perm1*KO mice. N=9-12.

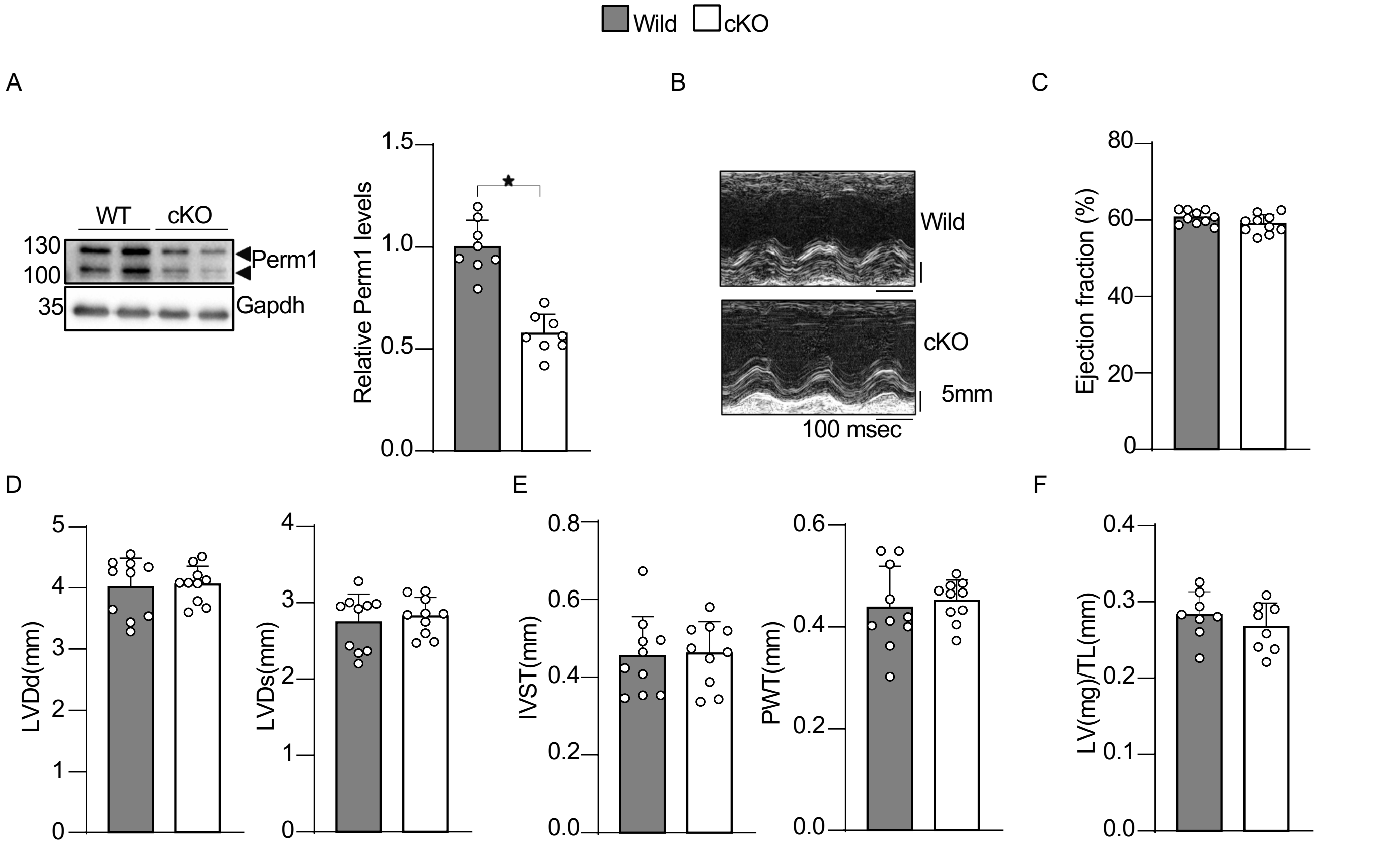

Figure S2. Normal baseline cardiac phenotype in cardiac-specific Perm1 heterozygous knockout (cKO) mice. (A) Relative Perm1 protein levels in heart tissue confirm reduced expression in cardiac-specific Perm1 heterozygous cKO mice. (B–E) Echocardiographic evaluation reveals no significant differences in cardiac structure or function between cKO and wild-type (WT) mice under baseline conditions, including (B) representative M-mode images, (C) ejection fraction, (D) left ventricular (LV) chamber dimensions, and (E) LV wall thickness. (F) The LV/tibia length (TL) ratio indicates no evidence of cardiac hypertrophy in cKO mice compared to WT controls. Statistical significance was determined by Student's t-test. N=8(A), and 10 (C-F). *$p < 0.05$.

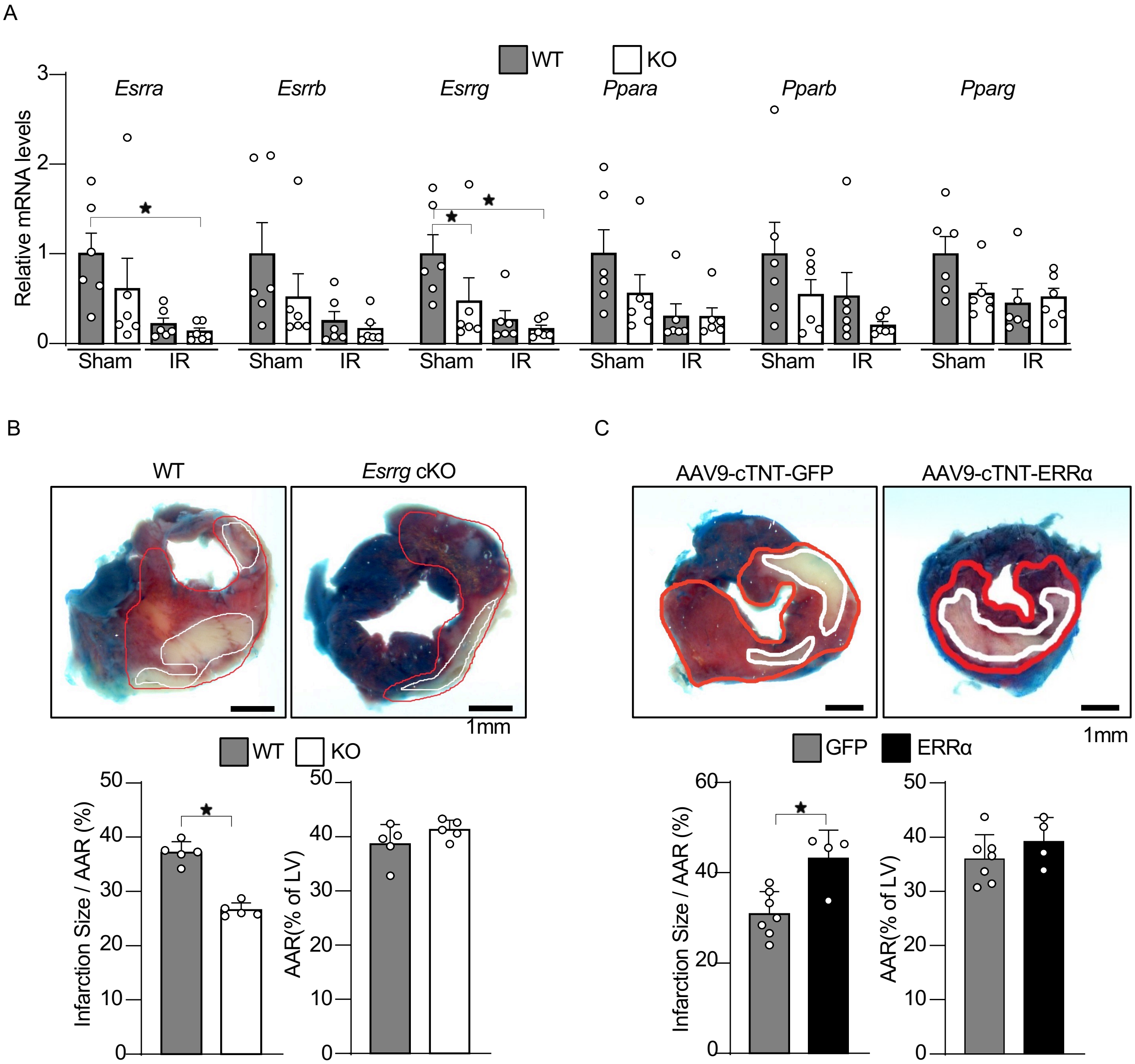

Figure S3. Downregulation of ERR protects the heart against IR. (A) Perm1 maintained the mRNA levels of *Esrra*, *Esrrb*, *Esrrg*, *Ppara*, *Pparb*, and *Pparg*. The indicated mRNA levels were examined in *Perm1* KO mice with IR. (B) Loss of *Esrrg* protects against IR injury. *Esrrg* hetero cKO mice were subjected IR. (C) ERRα overexpression exacerbated IR injury. The hearts transduced with AAV9-cTNT-ERRα or AAV9-cTNT-GFP (control) were subjected to IR. (B-C) The MI size was evaluated with TTC staining. Statistical significance was determined by ANOVA (A) and Student's t-test (B and C). N=6(A), 5(B) and 4-7(C). *$p < 0.05$.

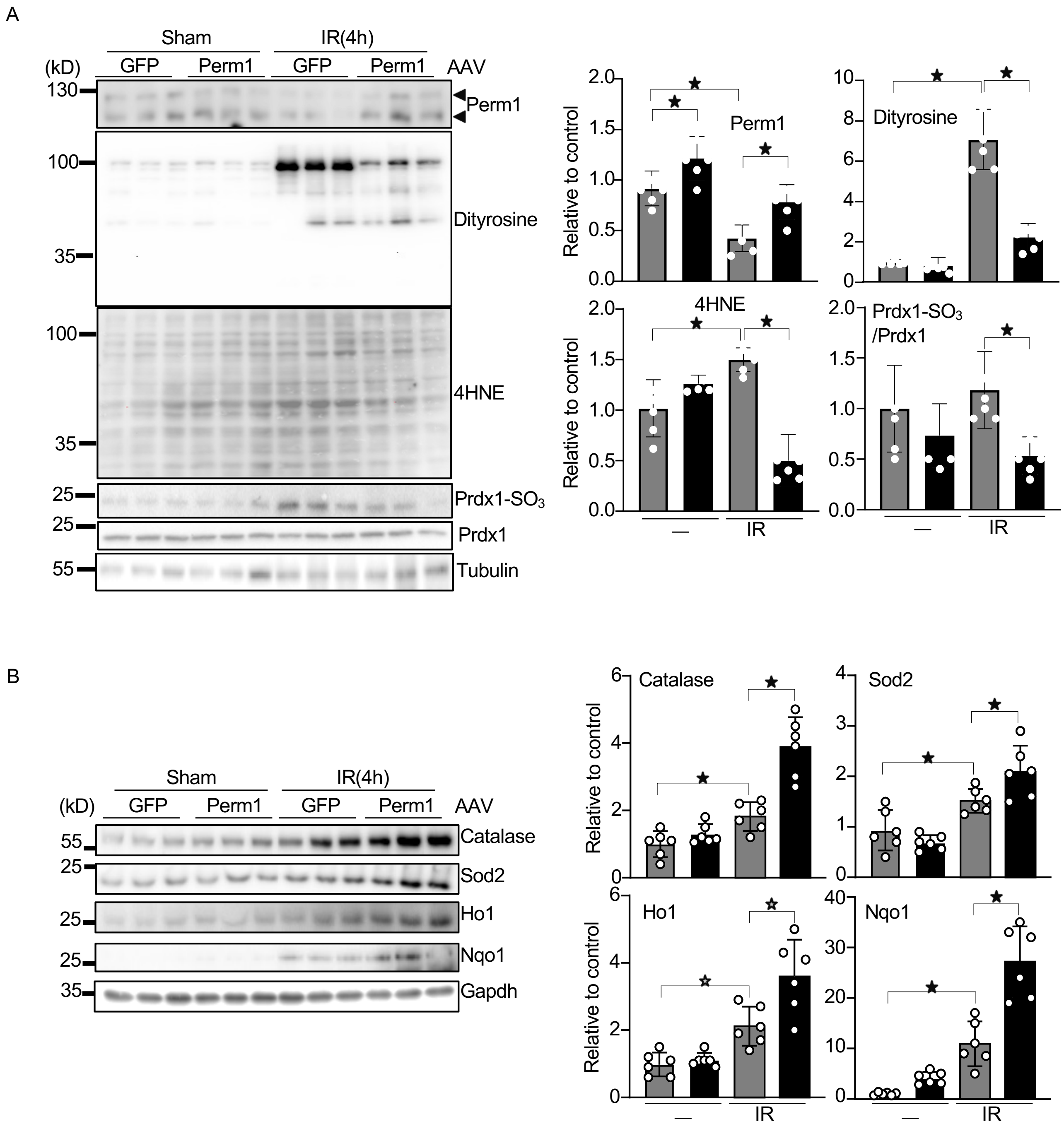

Figure S4 (A) Perm1 attenuates IR-induced oxidative stress. Indicated oxidative stress markers were examined. (B) Perm1 enhanced induction of antioxidant enzymes in response to IR. Statistical significance was determined with ANOVA. N= 6(E-F). *p < 0.05.

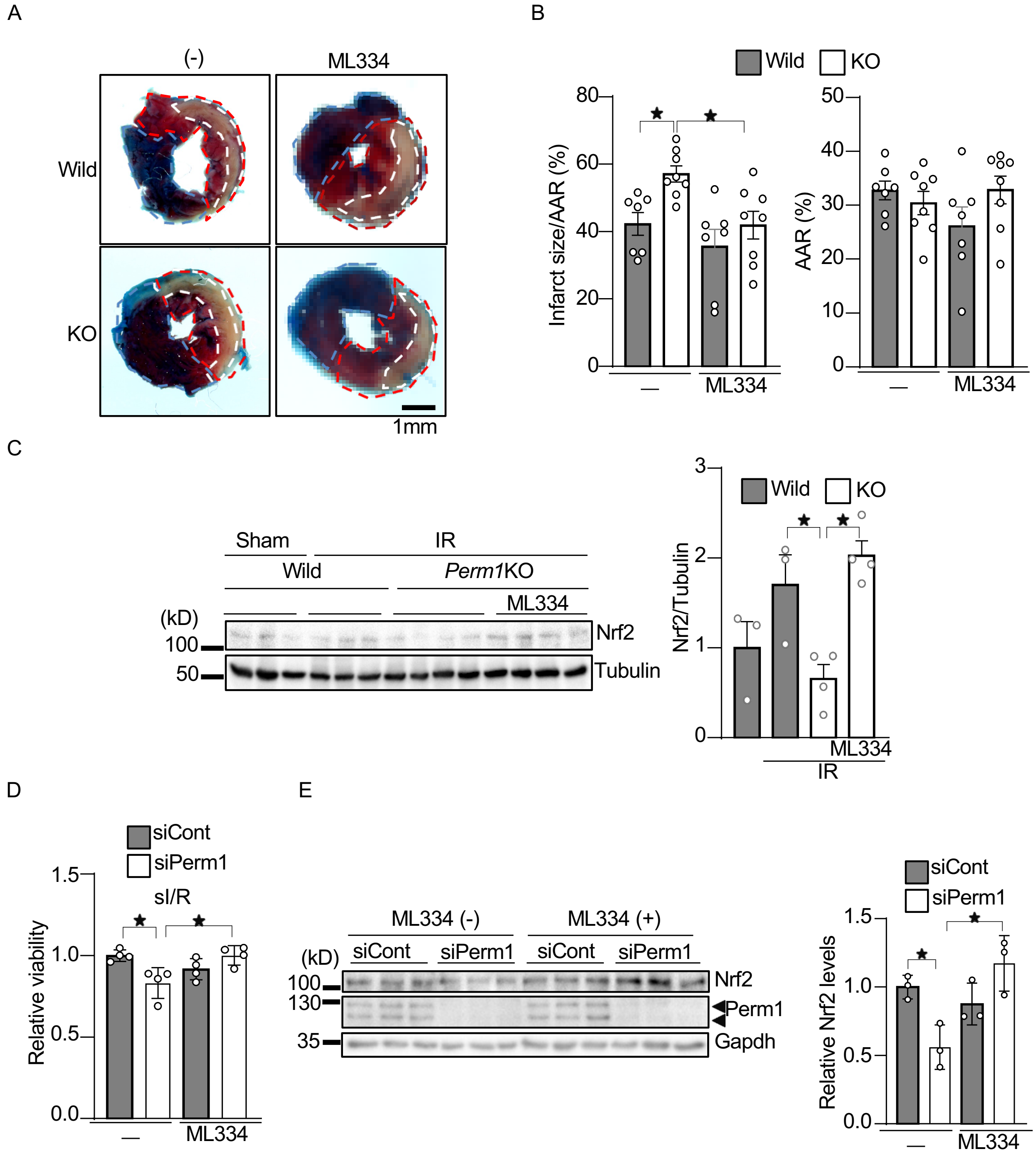

Figure S5. Releasing Nrf2 from Keap1 with ML334 normalizes IR injury in the presence of Perm1 downregulation. (A-B) ML334 attenuates IR injury in Perm1 KO mice. Mice were treated with ML334 and then subjected to IR. AB: ML334 normalized IR injury in *Perm1* KO mice. (C) ML334 normalized Nrf2 in *Perm1* KO mice. (D-E) ML334 attenuates cell death in cardiomyocytes with Perm1 knockdwon. Cultured cardiomyocytes transfected with either siControl or siPerm1 were subjected to sIR in the presence or absence of ML334. Cardiomyocyte viability was evaluated with CellTiter Blue assays (D). The cellular levels of Nrf2 and Perm1 were evaluated (E). Statistical significance was determined with ANOVA (A-E). N=7-8 (A), 3-4 (B), 4(D) and 3(E). *p<0.05.

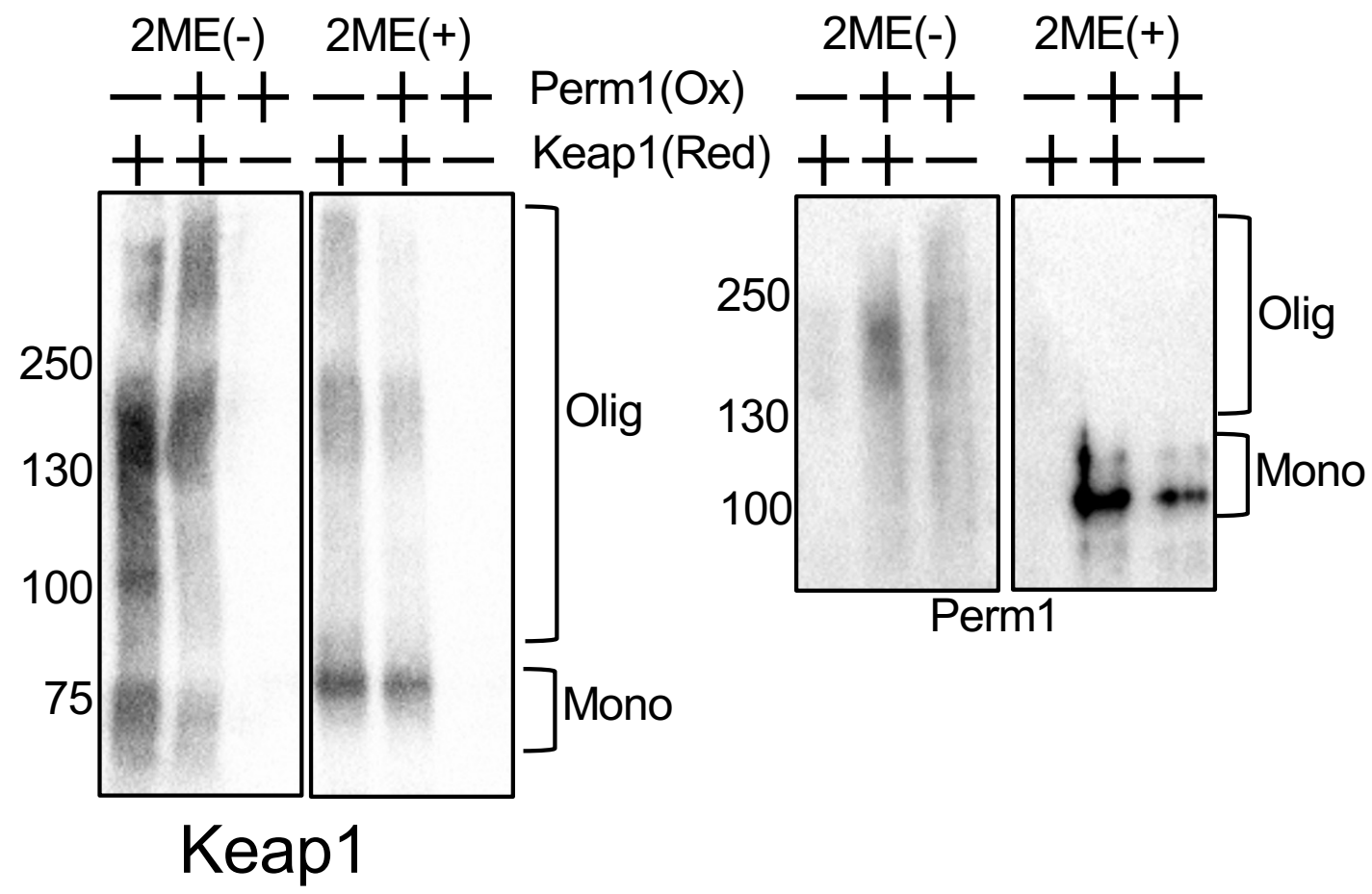

Figure S6. Thiol-disulfide exchange reaction between Perm1 and Keap1. Recombinant Keap1 reduced with DTT and recombinant Perm1 oxidized with H_2O_2 were incubated together and analyzed by Western blot under non-reducing SDS-PAGE conditions. Representative data are shown from 2 independent experiments.
