# Supplementary material for: Perm1 enhances Nrf2-driven antioxidant defense through Keap1 oxidation during myocardial ischemia/reperfusion injury
Source: JCI Insight. 2026 May 12;11(14):e204132. doi: 10.1172/jci.insight.204132 (PMC13461155; doi:10.1172/jci.insight.204132)
Supplement: Supplementary file 2 [file jciinsight-11-204132-s360.pdf]

Unedited blot and gel images

Fig. 1A

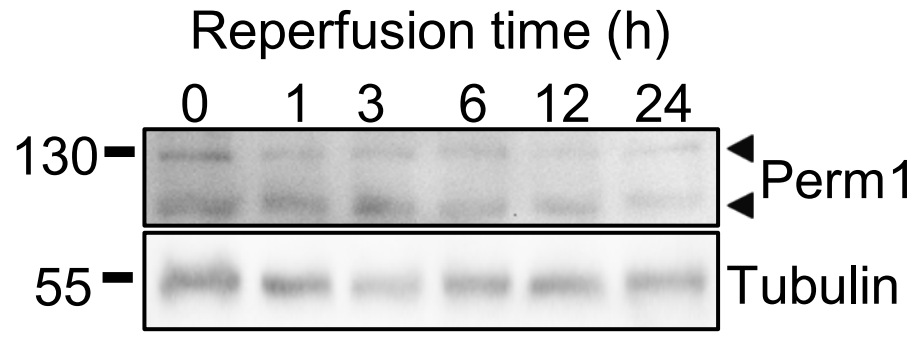

Full blot

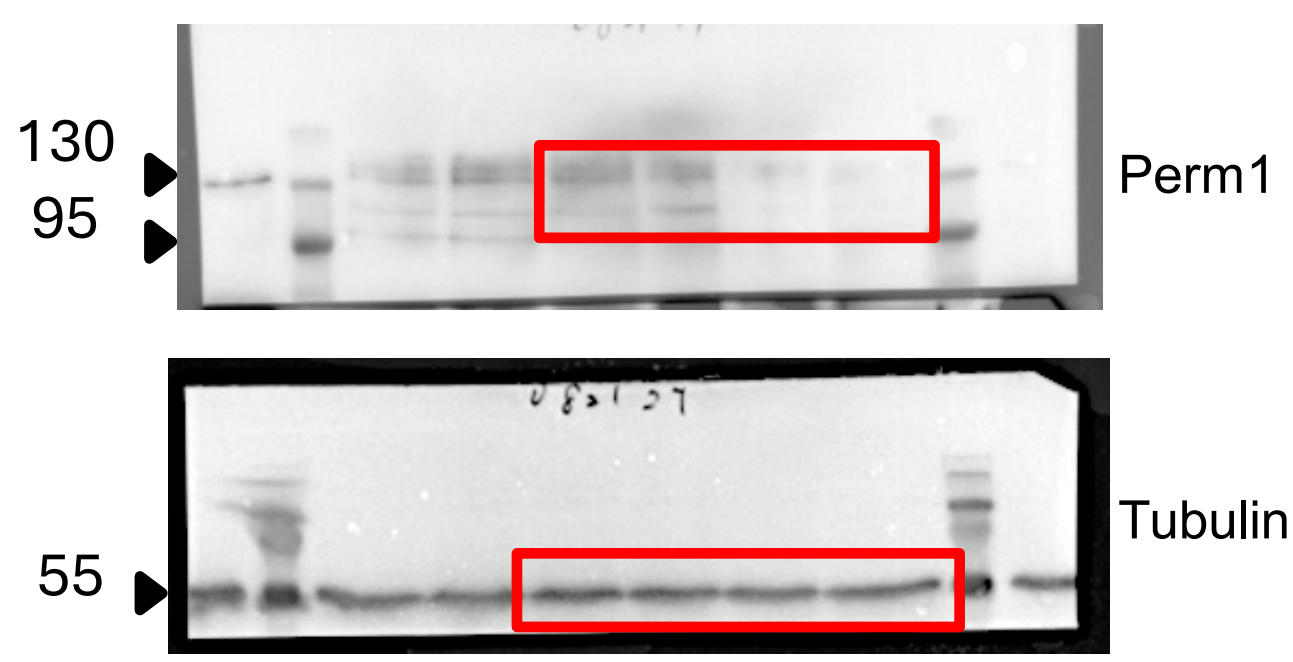

Fig. 1B

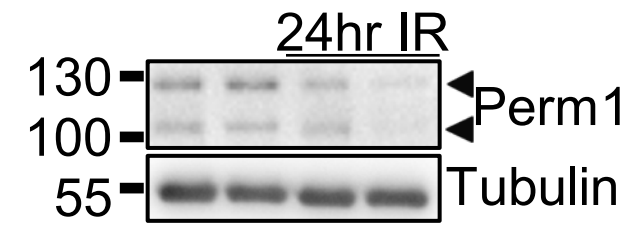

Full blot

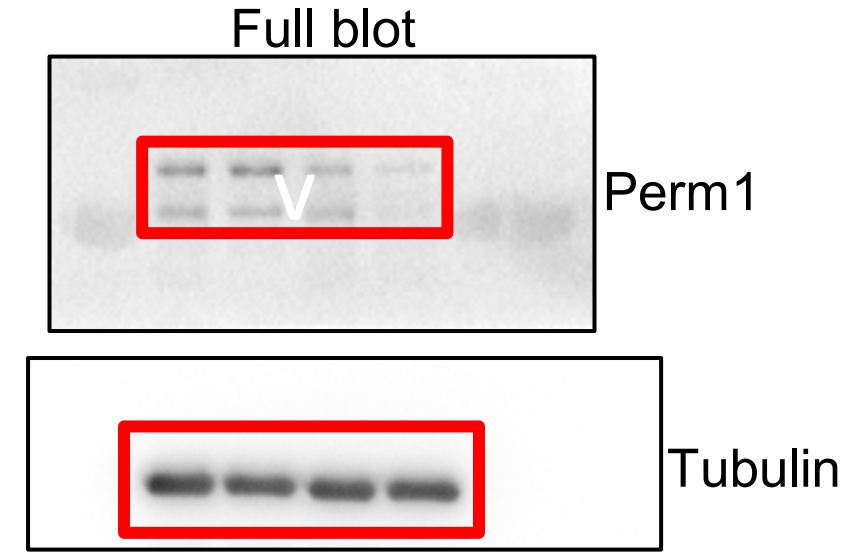

Fig. 1C

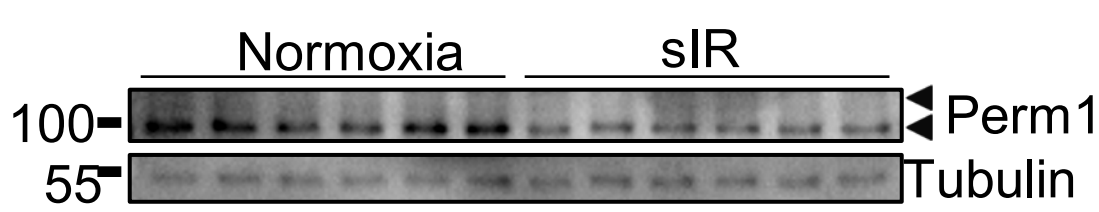

Full blot

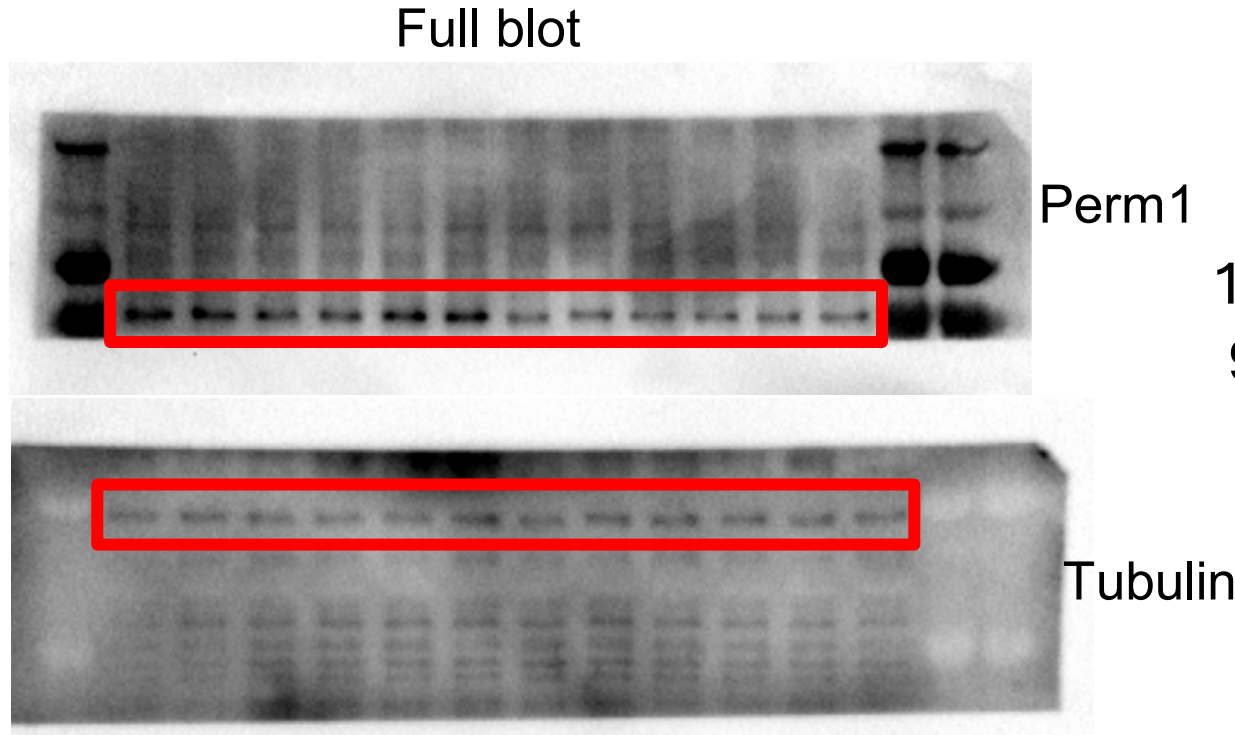

Fig. 1E

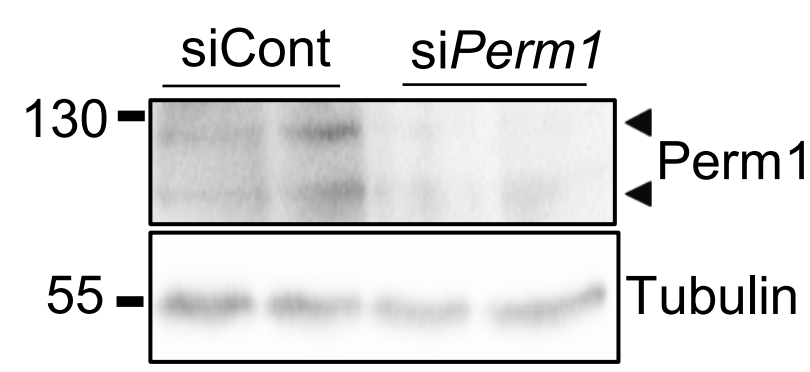

Full blot

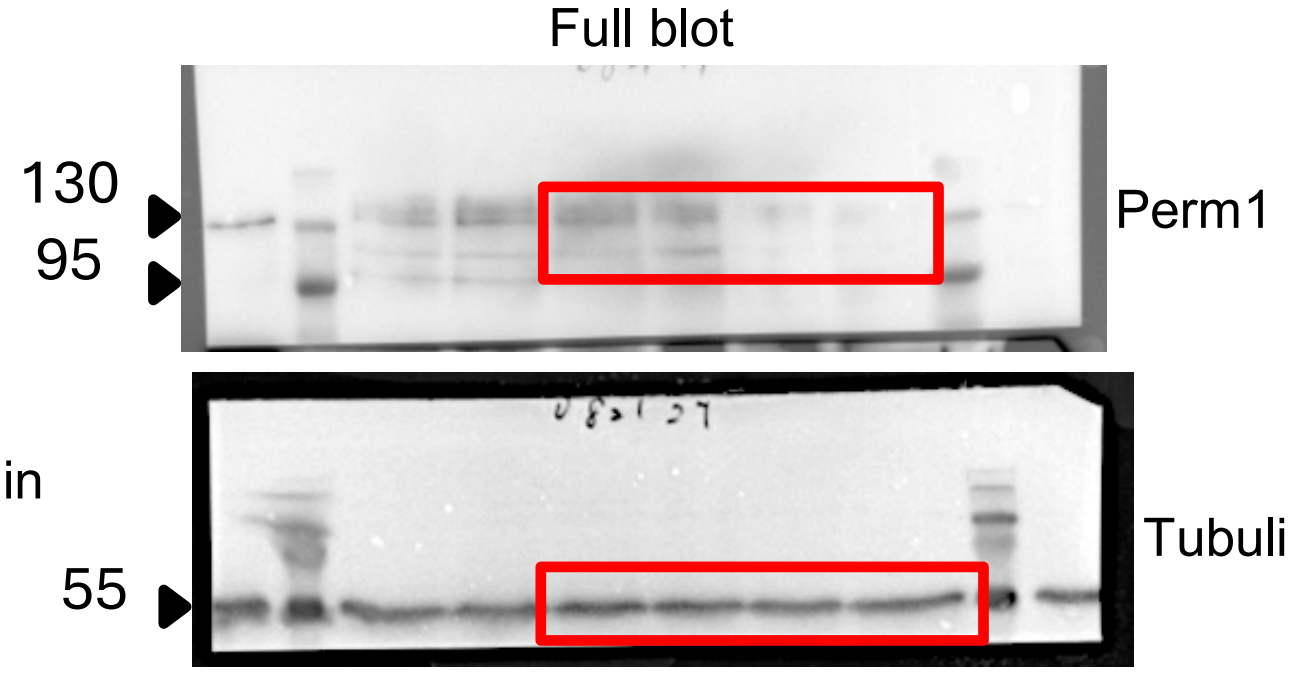

Fig. 2A

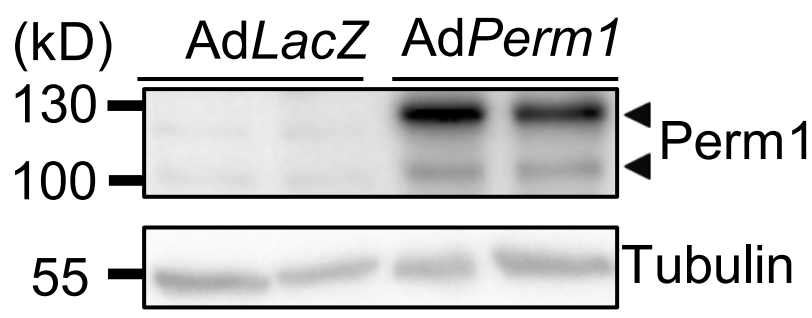

Full blot

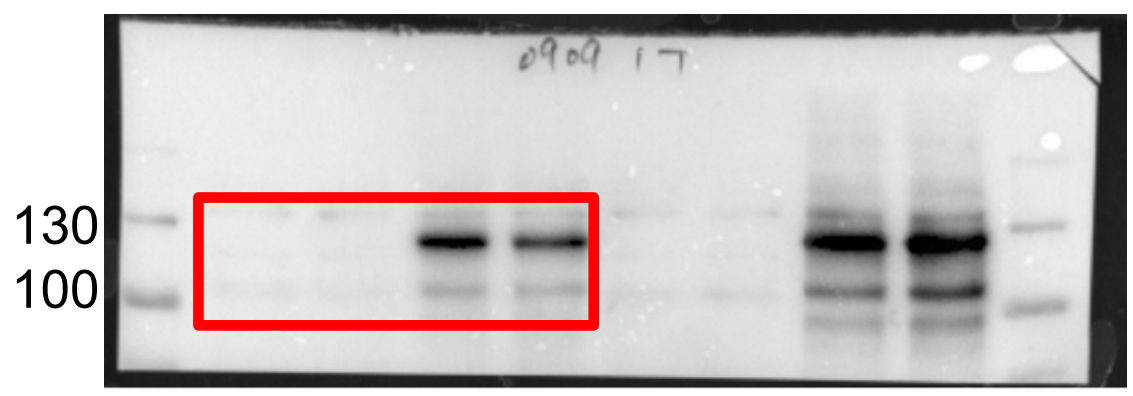

Perm1

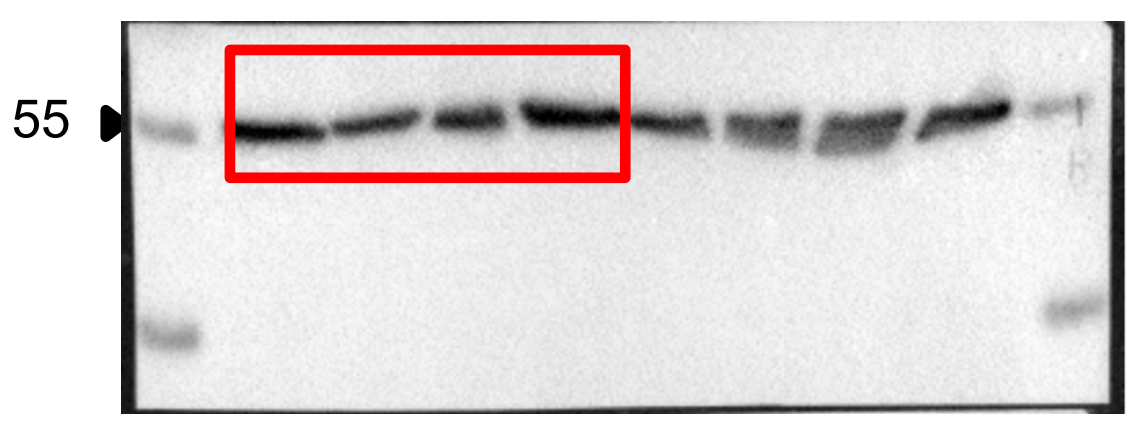

Tubulin

Fig. 2C

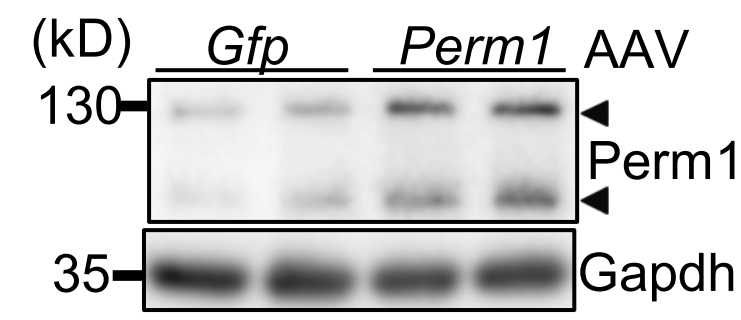

Full blot

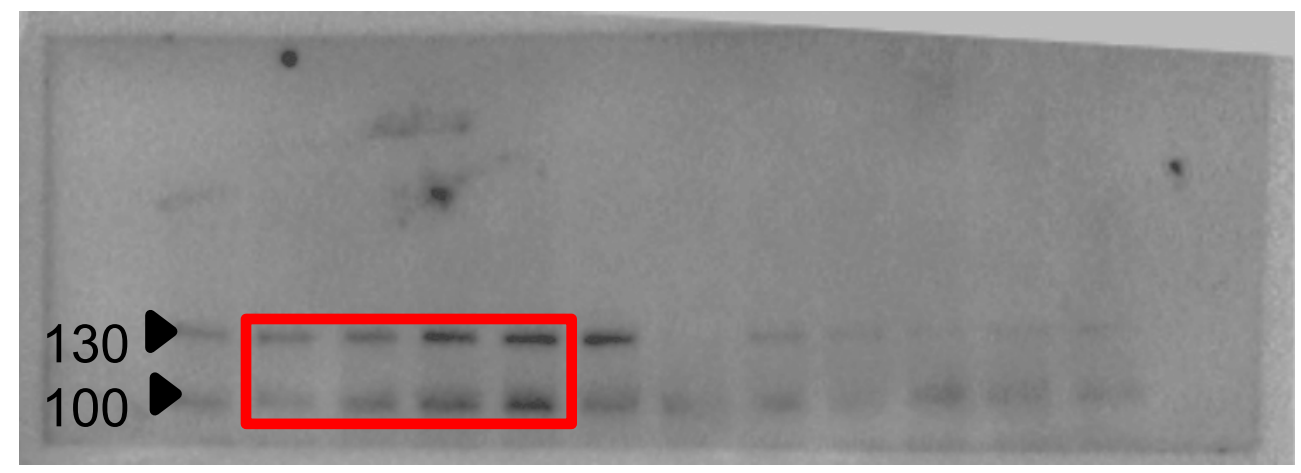

Perm1

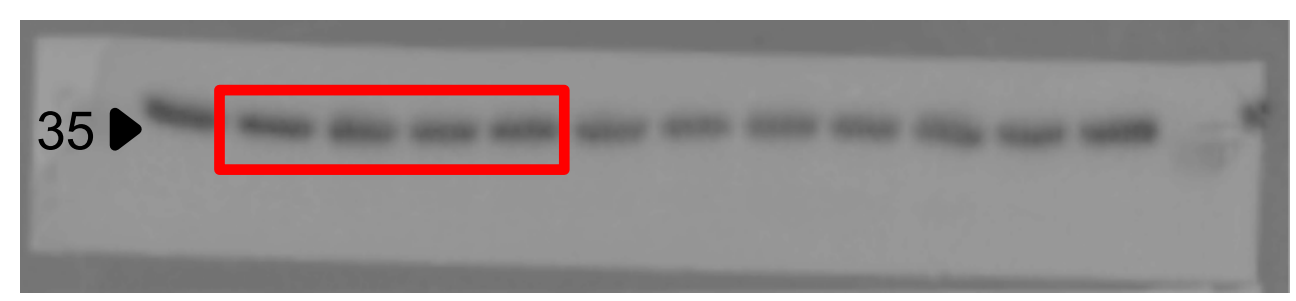

Gapdh

Fig. 3A

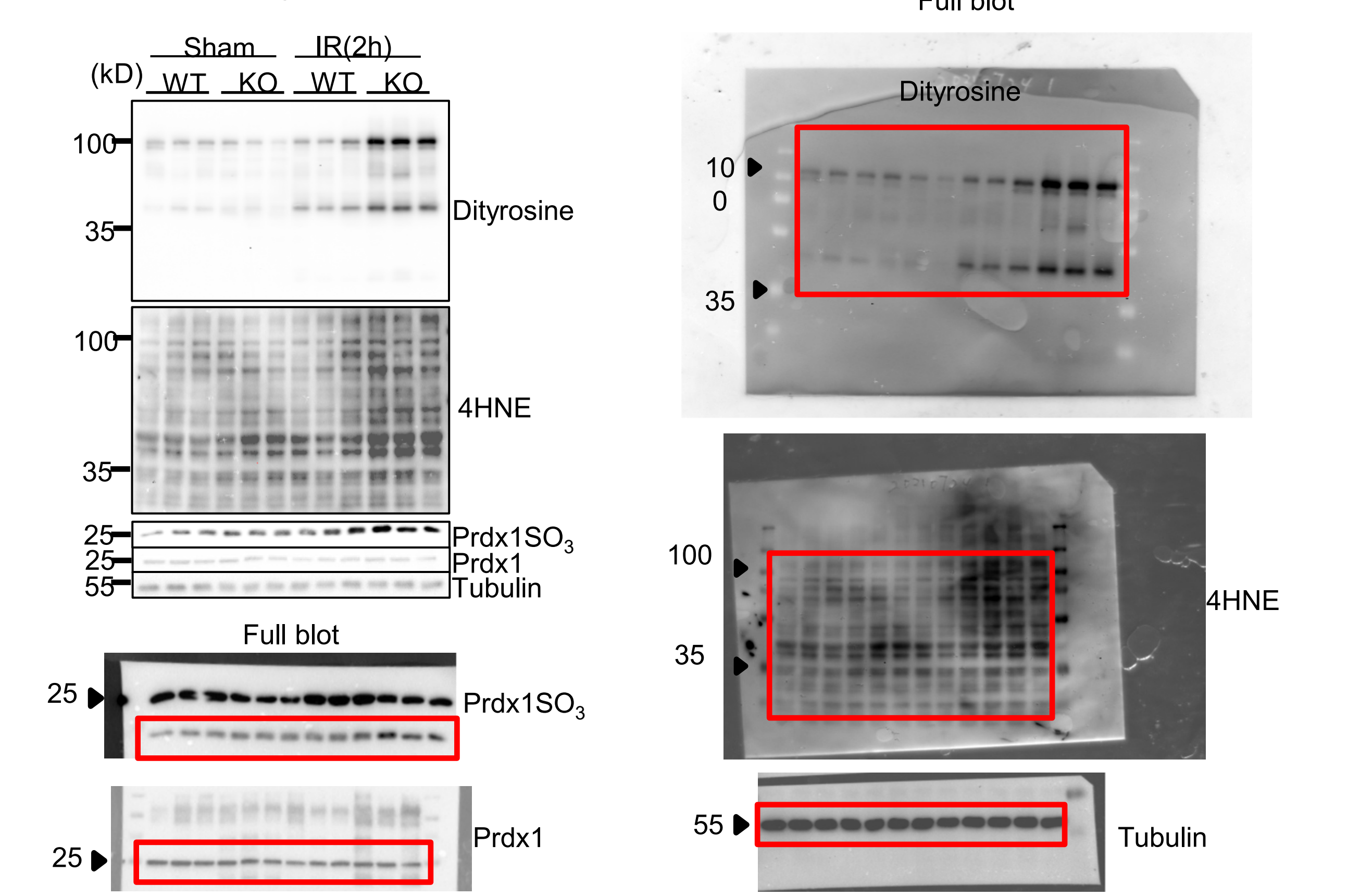
Full blot
Sham
IR(2h)
(kD)
WT
KO
WT
KO
100
35
Dityrosine
100
35
4HNE
25
25
55
$Prdx1SO_3$
Prdx1
Tubulin
Full blot
25
$Prdx1SO_3$
25
Prdx1
Dityrosine
10
0
35
100
35
4HNE
55
Tubulin

Fig. 3D

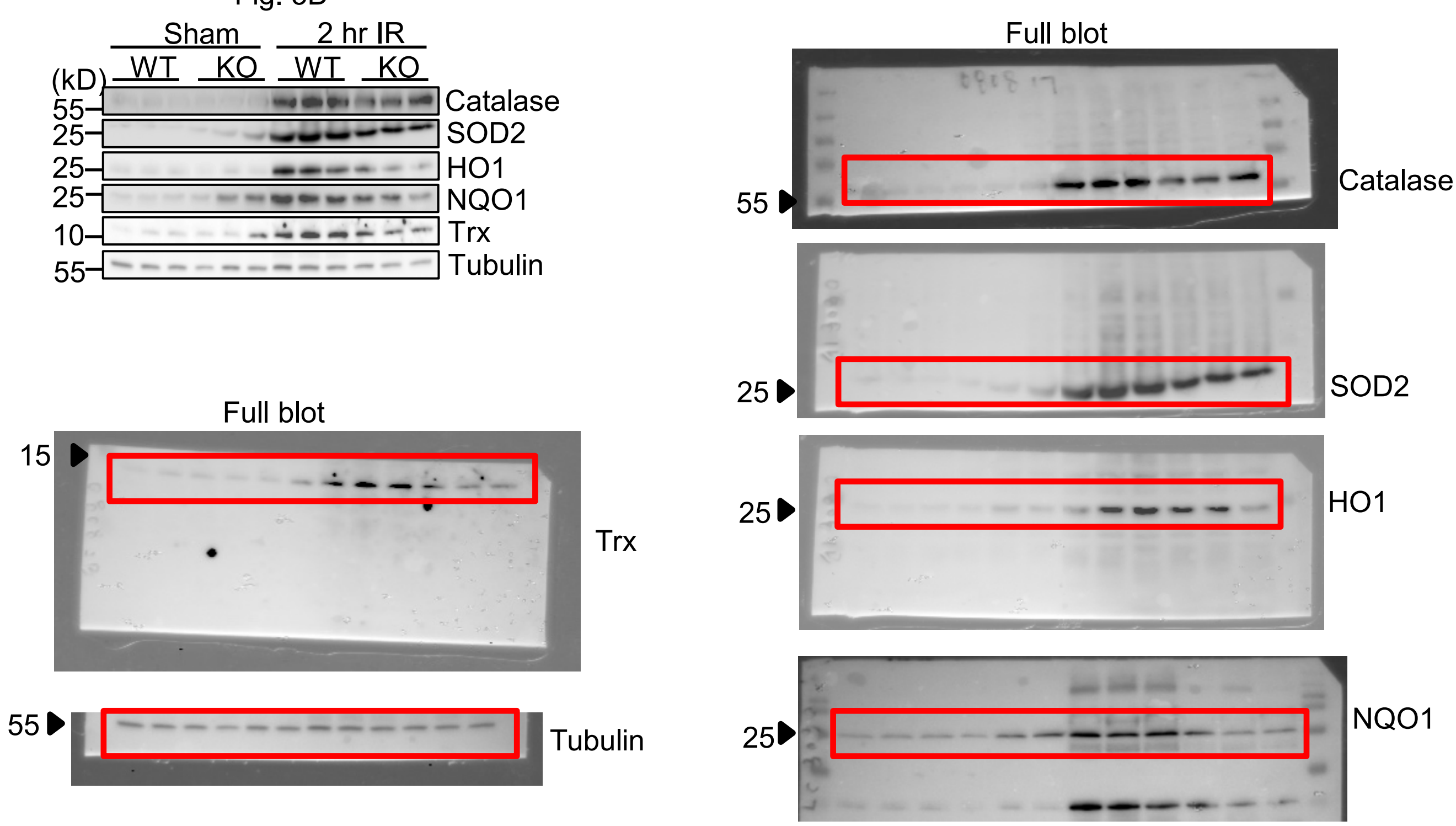
Sham
2 hr IR
WT
KO
WT
KO
(kD)
55
25
25
25
10
55
Catalase
SOD2
HO1
NQO1
Trx
Tubulin
Full blot
15
Trx
55
Tubulin
Full blot
55
Catalase
25
SOD2
25
HO1
25
NQO1

Fig. 4A

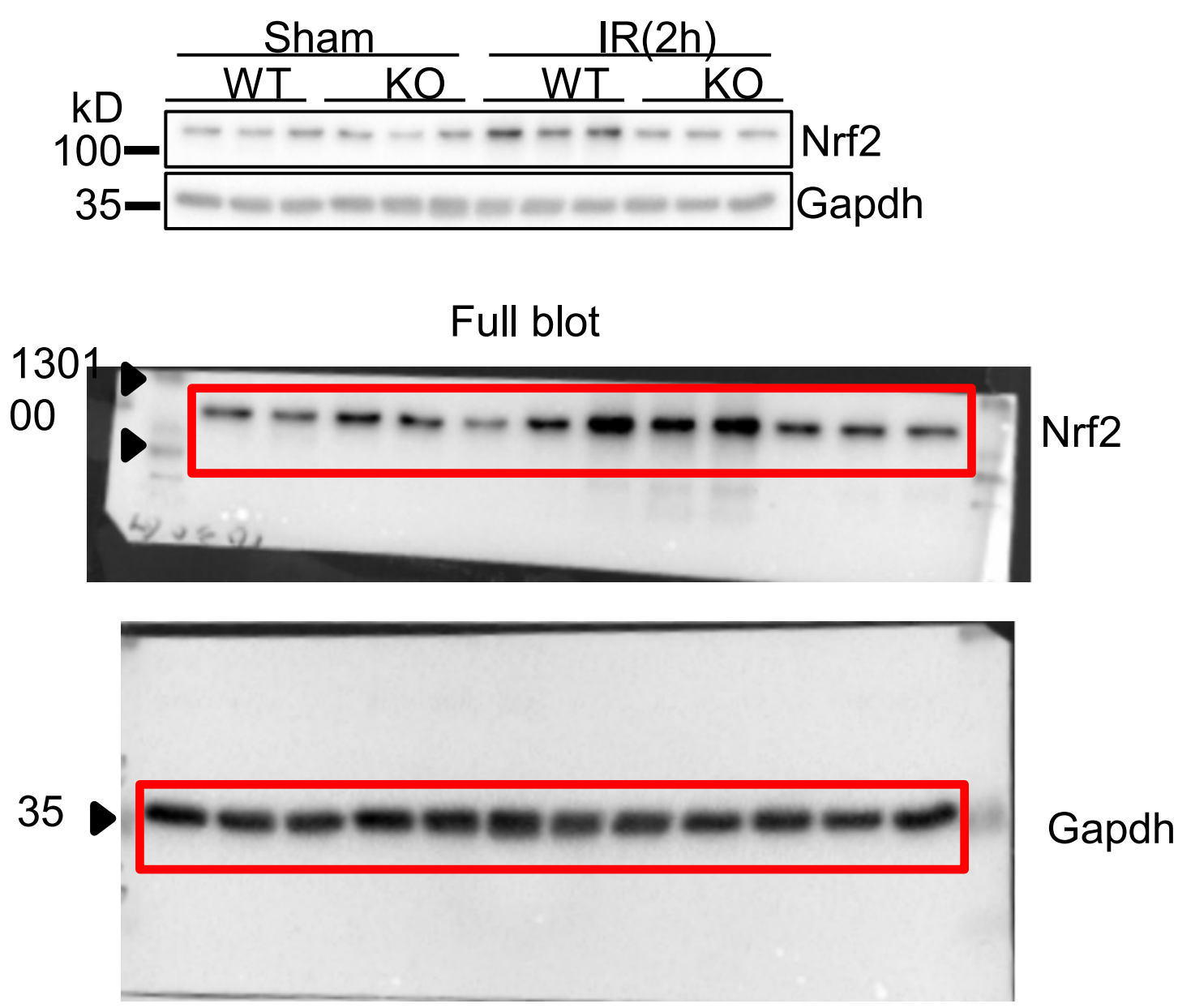

Fig. 4C

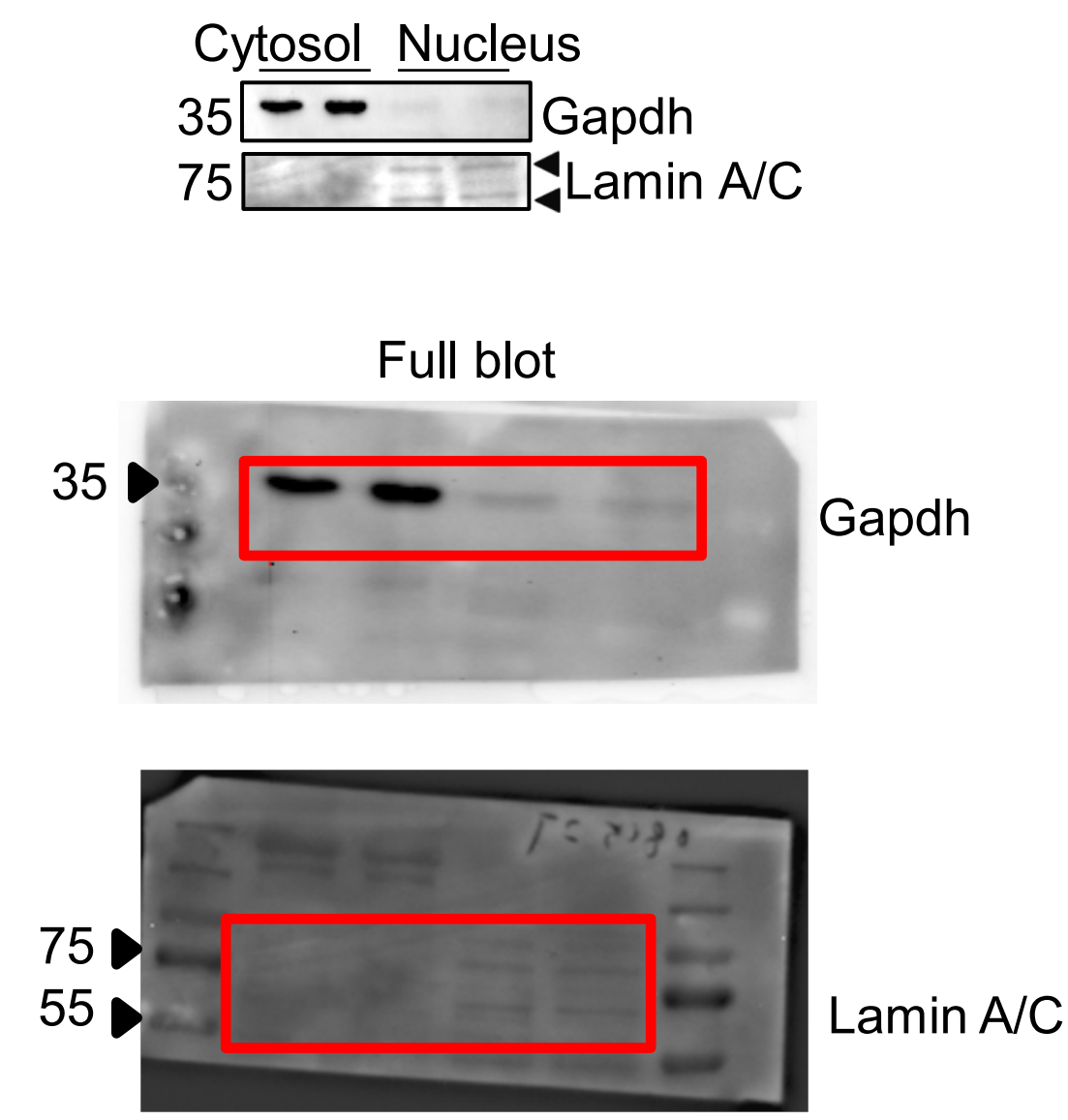

Fig. 4B

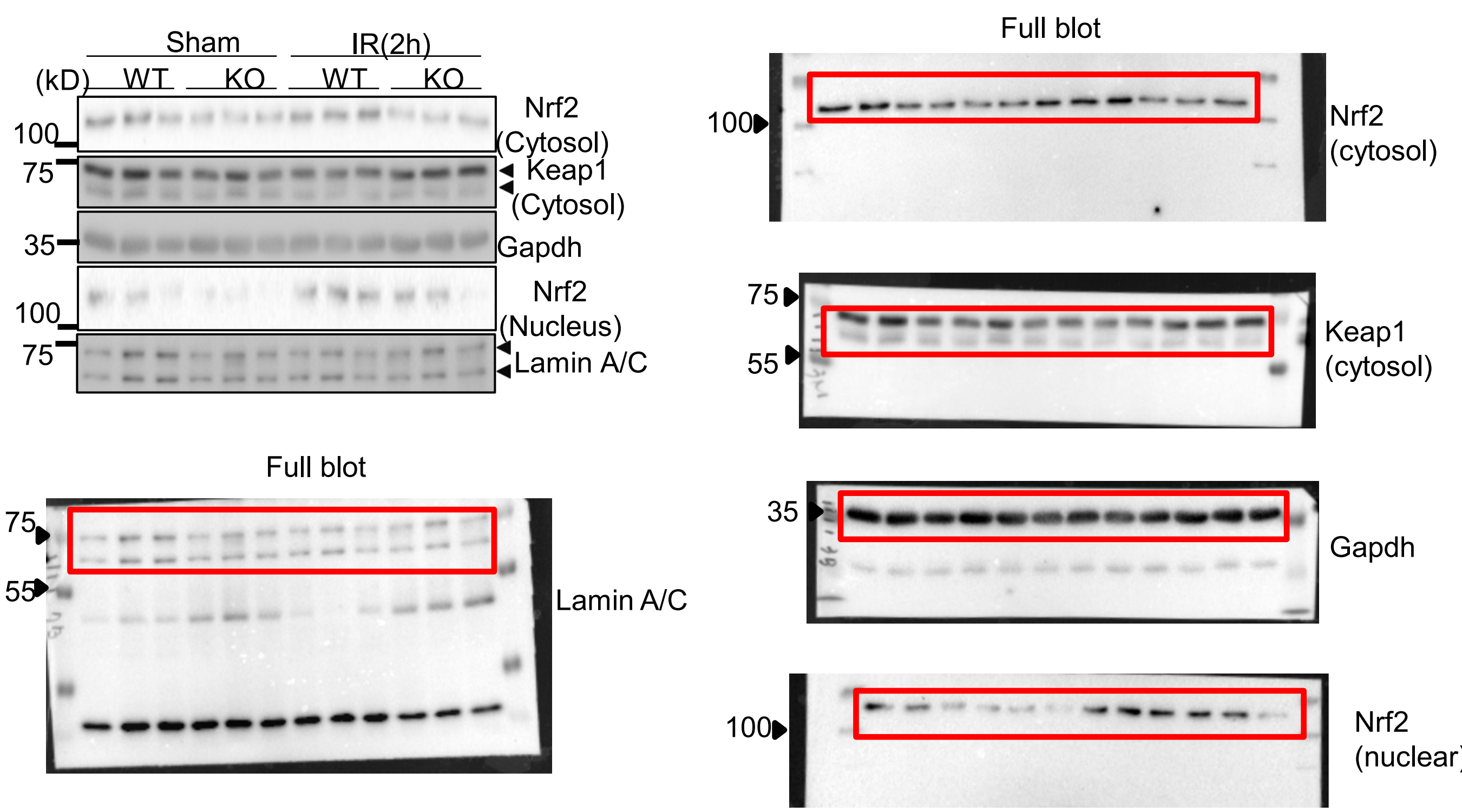

Fig. 5A

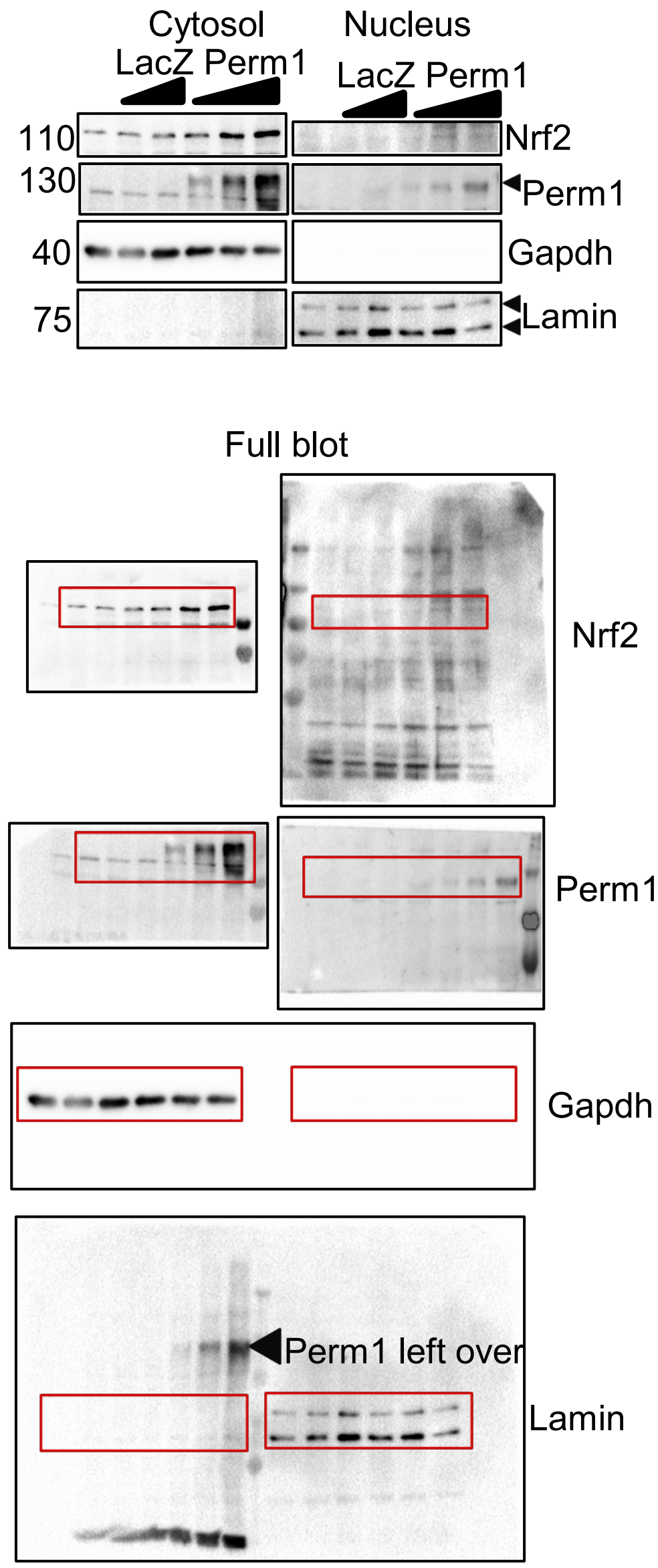
Cytosol
Nucleus
LacZ Perm1
LacZ Perm1
110
130
40
75
Nrf2
Perm1
Gapdh
Lamin
Full blot
Nrf2
Perm1
Gapdh
Perm1 left over
Lamin

Fig. 5D

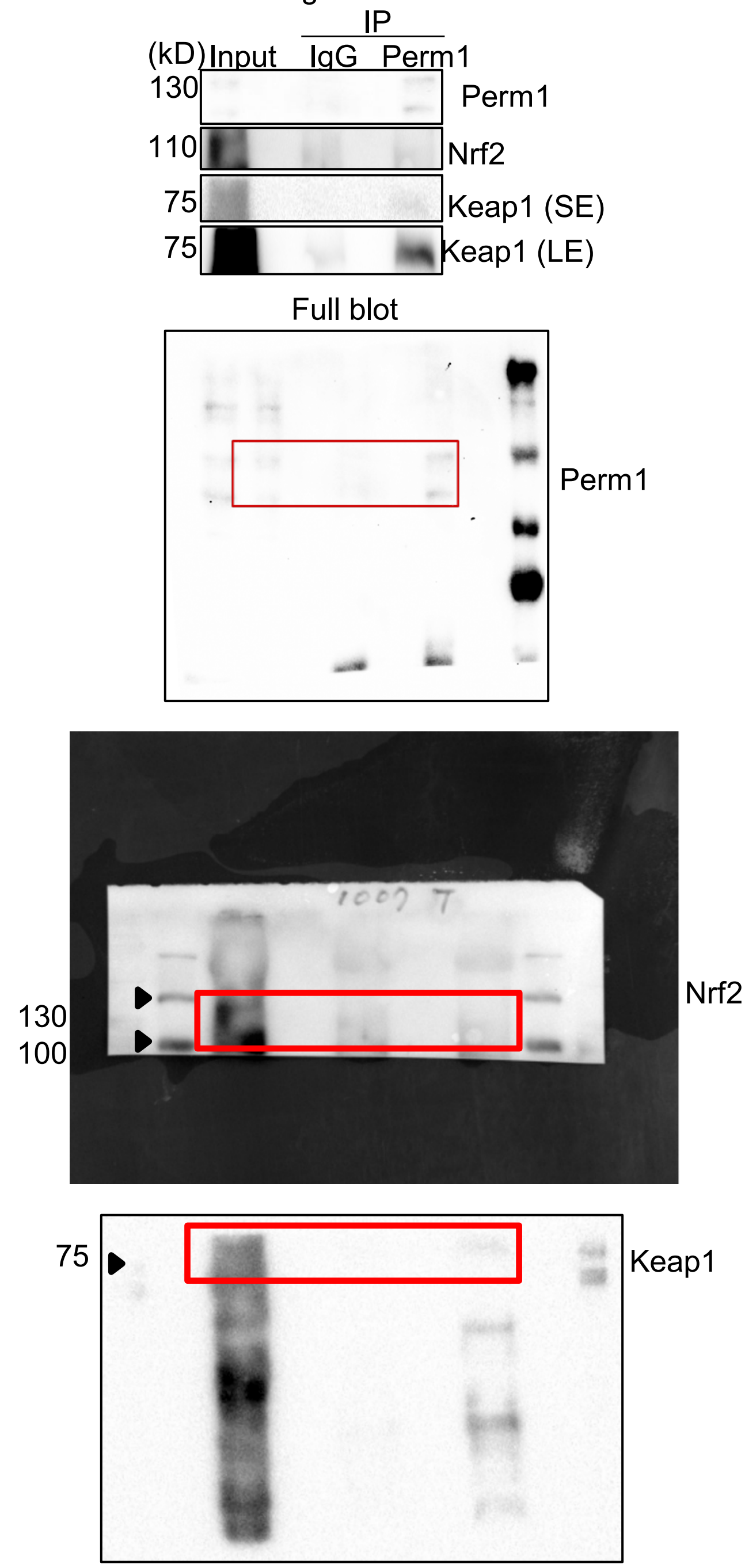
IP
(kD) Input IgG Perm1
130
110
75
75
Perm1
Nrf2
Keap1 (SE)
Keap1 (LE)
Full blot
Perm1
130
100
Nrf2
75
Keap1

Fig. 5E

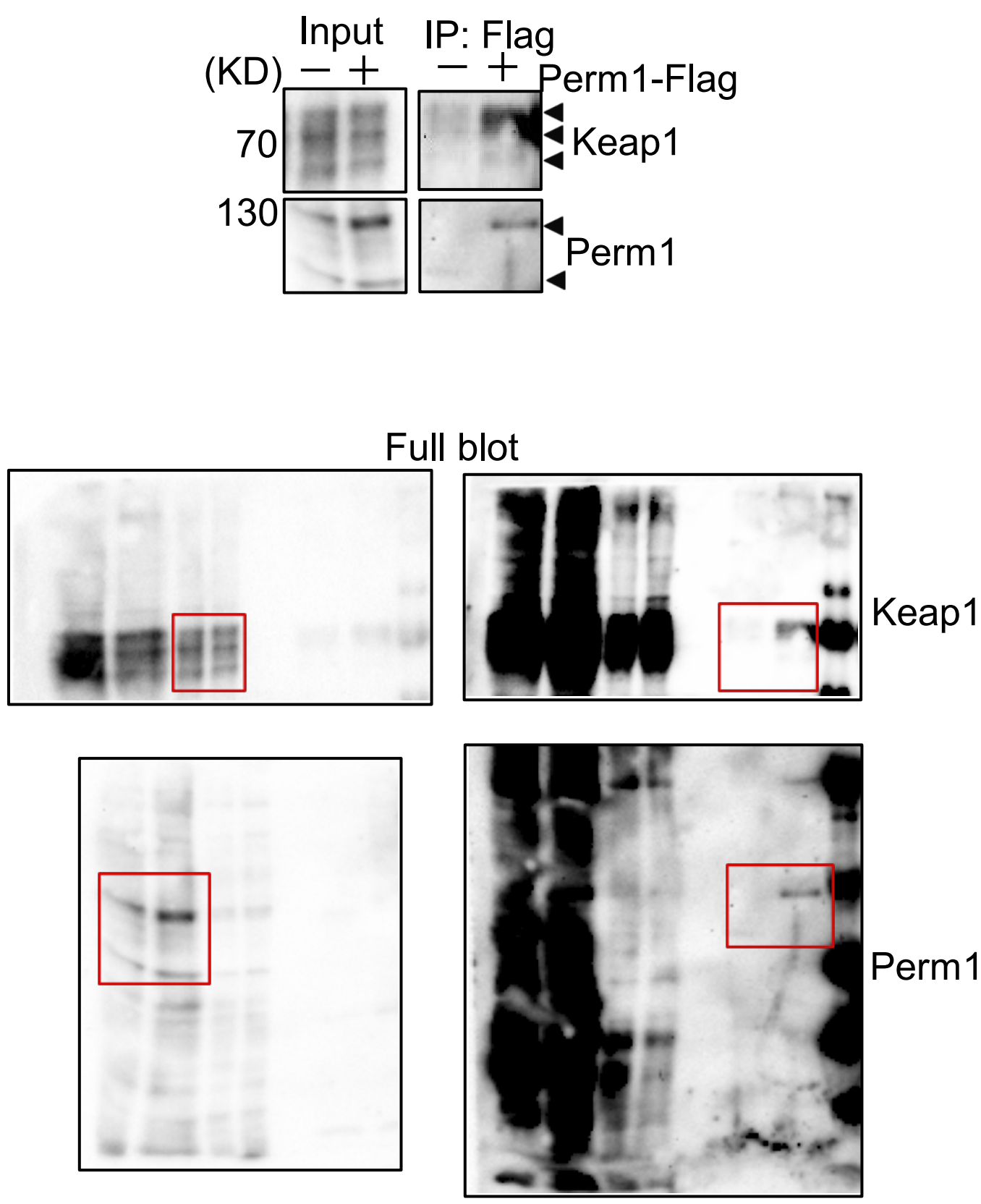
Input
IP: Flag
(KD)
Perm1-Flag
70
Keap1
130
Perm1
Full blot
Keap1
Perm1

Fig. 5F

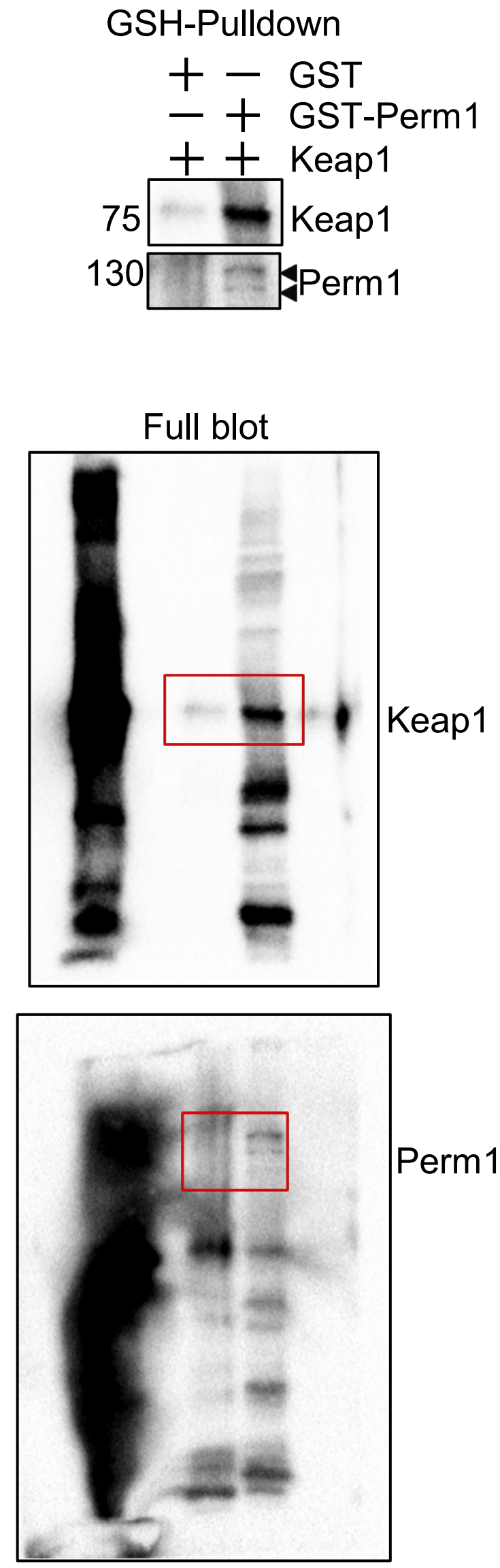
GSH-Pulldown
GST
GST-Perm1
Keap1
75
Keap1
130
Perm1
Full blot
Keap1
Perm1

Fig. 5G

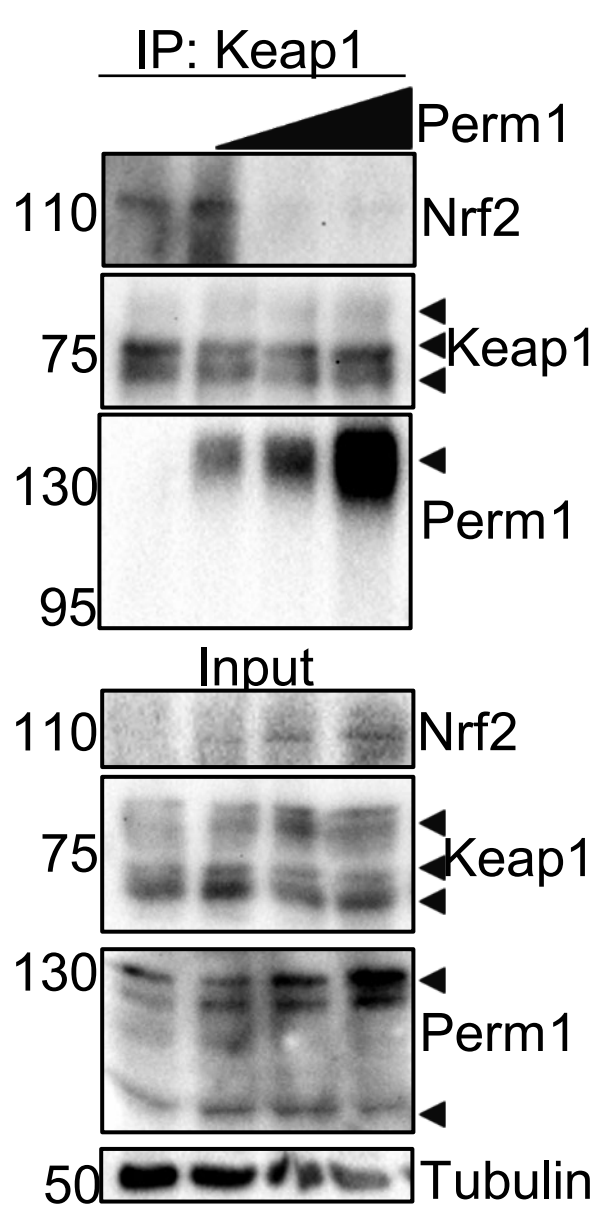

Full blot

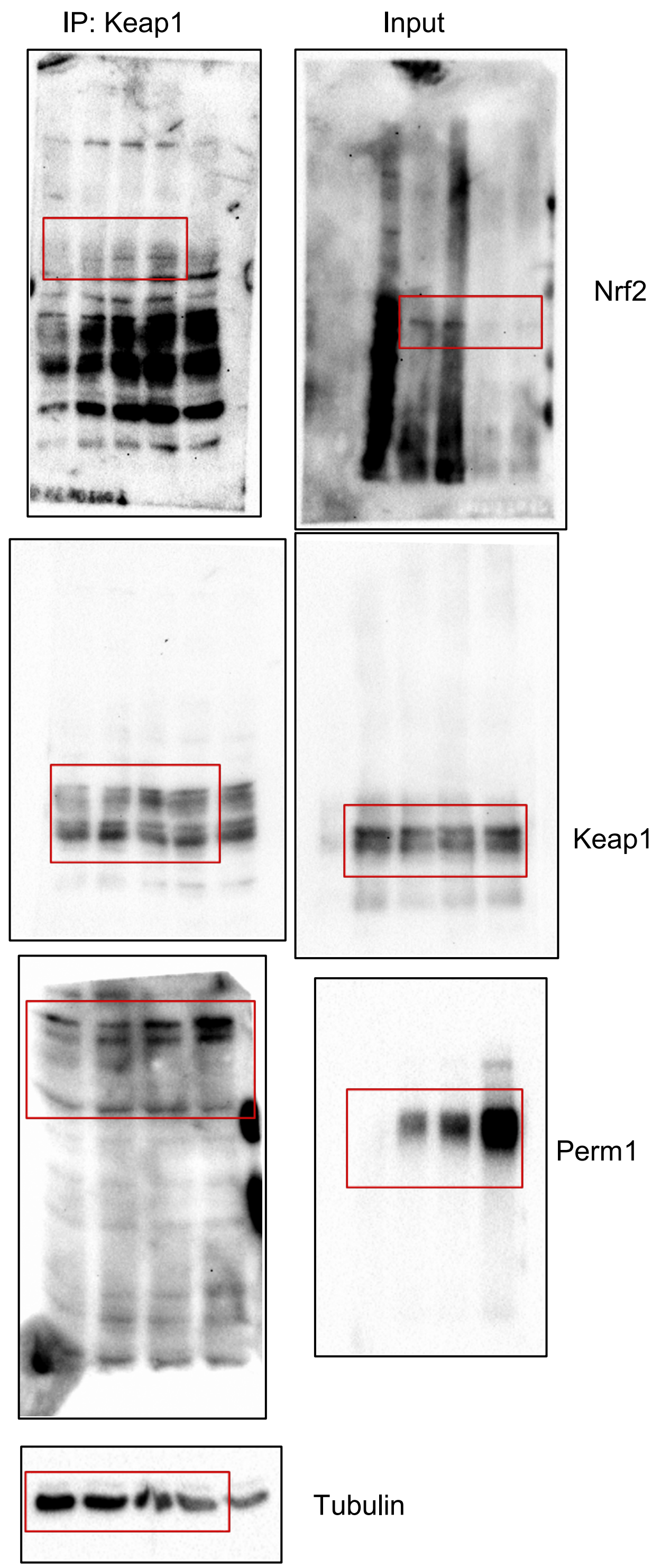

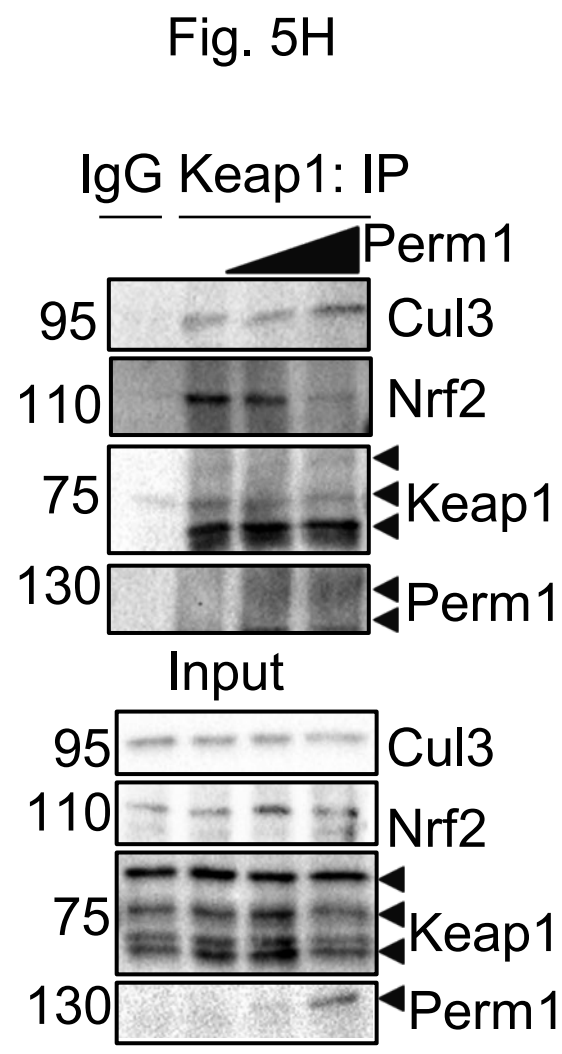
Fig. 5H
IgG Keap1: IP
Perm1
95
Cul3
110
Nrf2
75
Keap1
130
Perm1
Input
95
Cul3
110
Nrf2
75
Keap1
130
Perm1

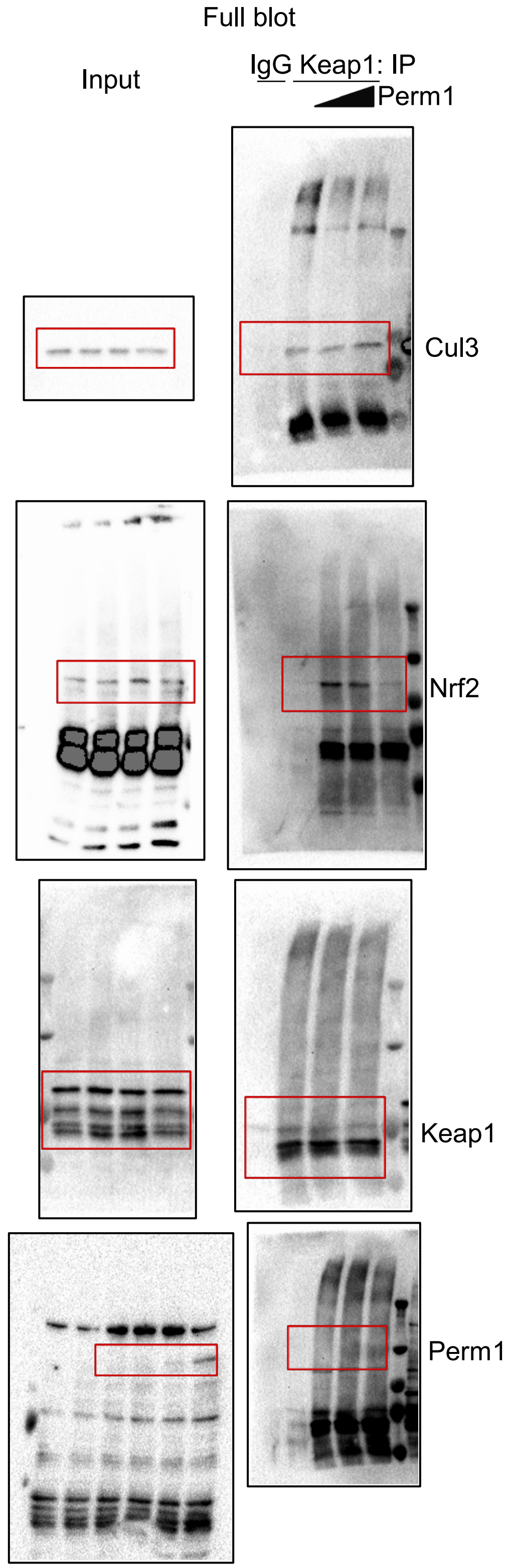
Full blot
Input
IgG Keap1: IP
Perm1
Cul3
Nrf2
Keap1
Perm1

Figure 5I

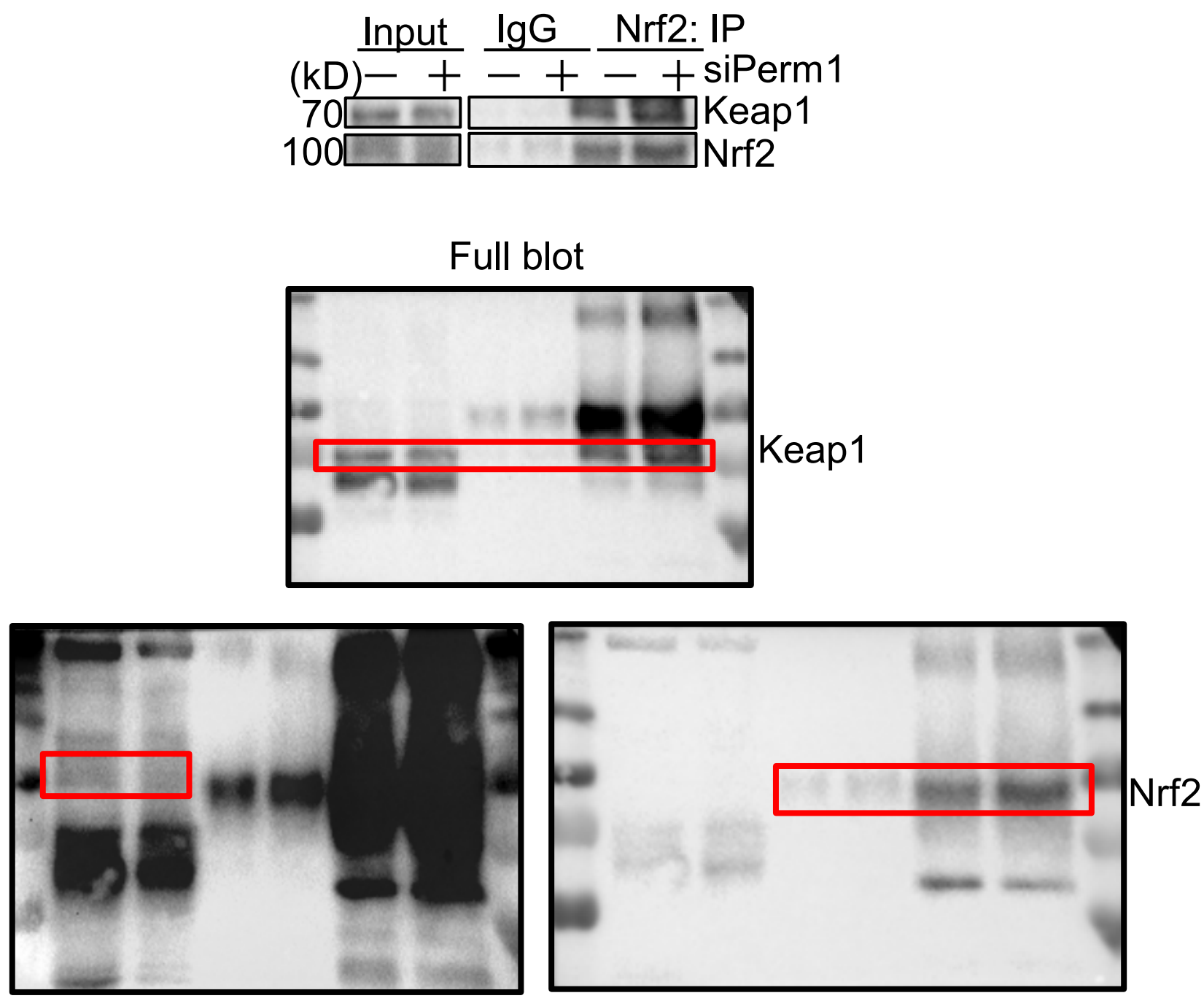
Input
IgG
Nrf2: IP
(kD)
— + — + — +
siPerm1
70
100
Keap1
Nrf2
Full blot
Keap1
Nrf2

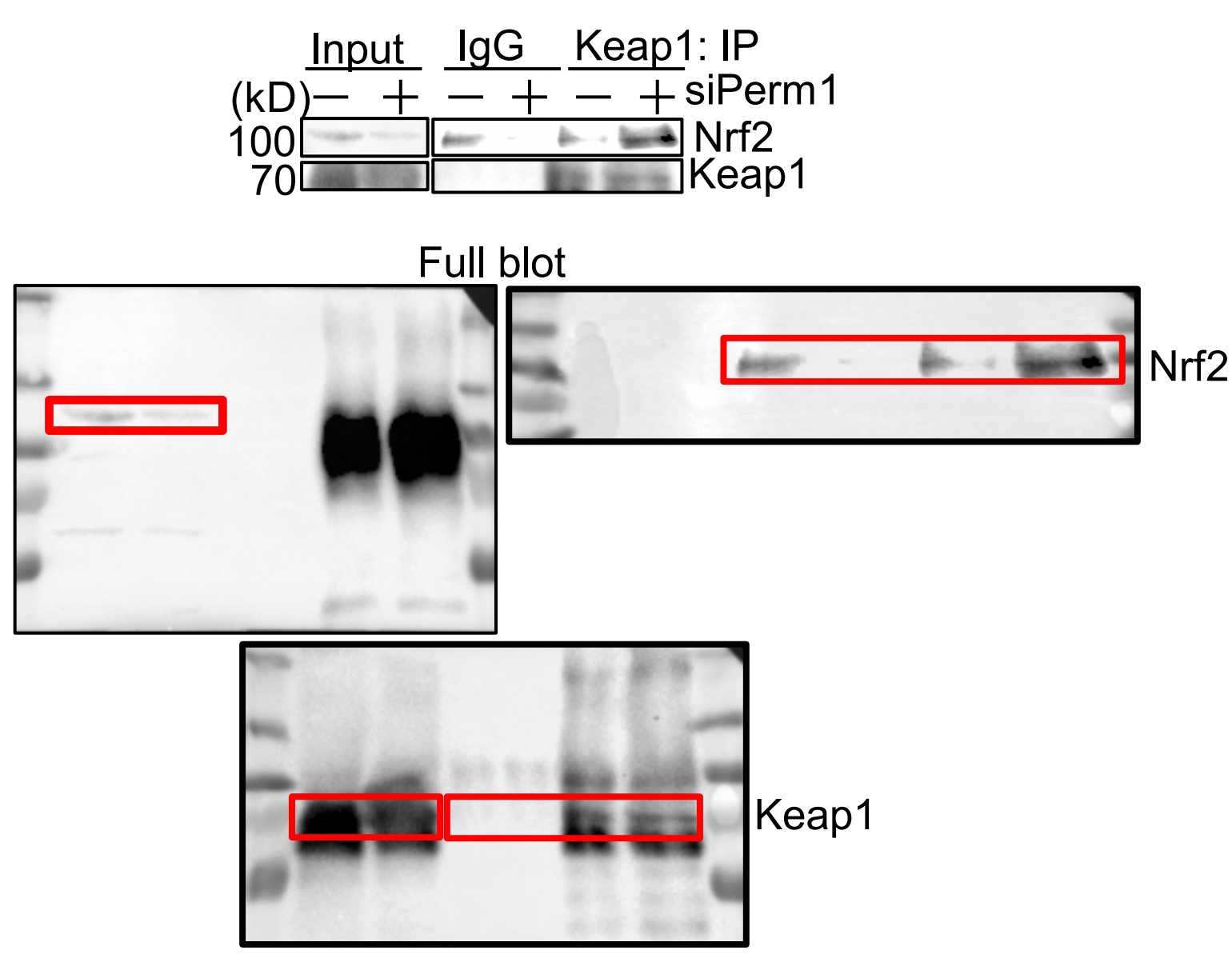
Input
IgG
Keap1: IP
(kD)
— + — + — +
siPerm1
100
70
Nrf2
Keap1
Full blot
Nrf2
Keap1

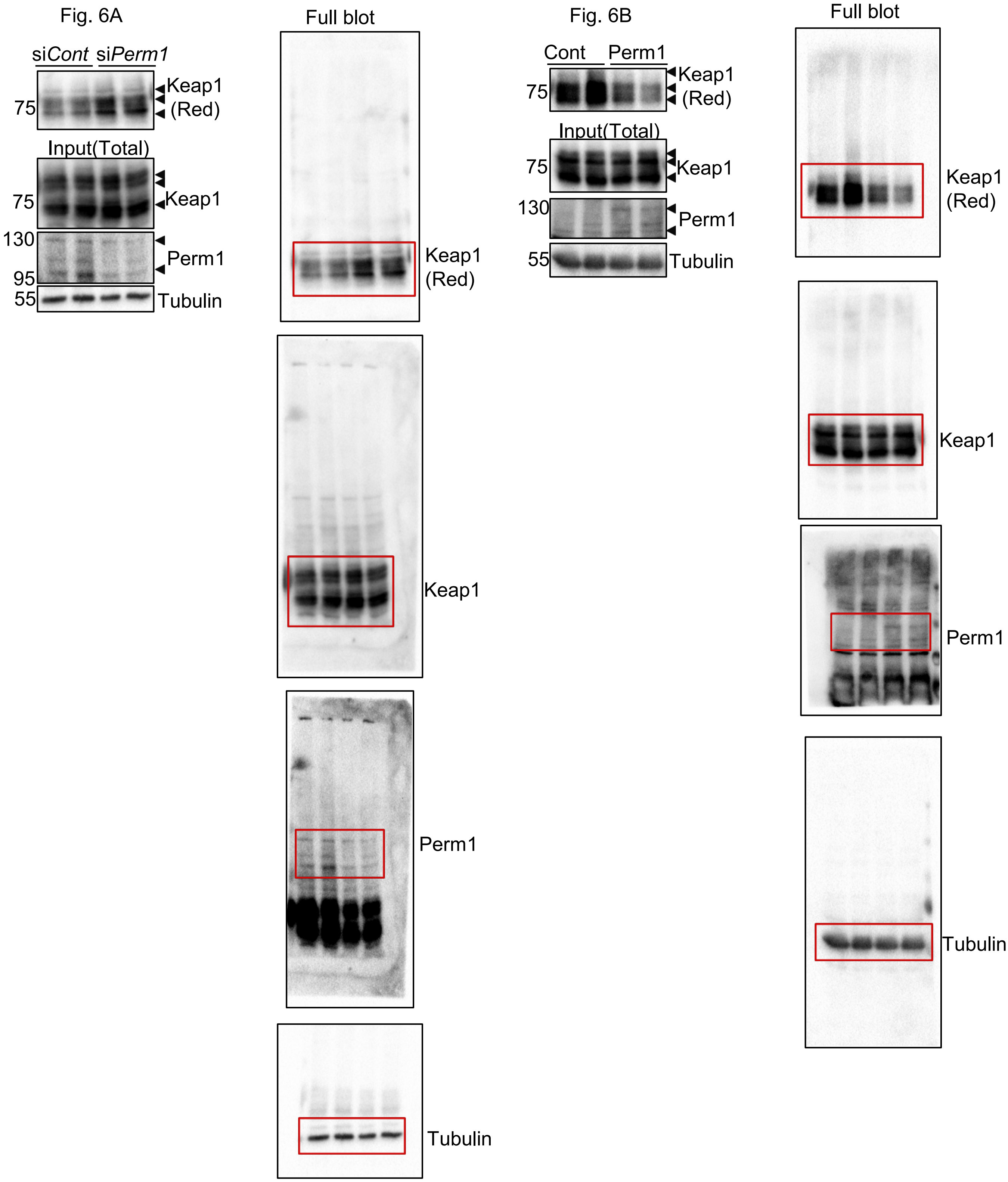

Fig. 6A
Full blot
siCont siPerm1
Keap1
(Red)
75
Input(Total)
75
Keap1
130
Perm1
95
55
Tubulin
Keap1
(Red)
Keap1
Perm1
Tubulin
Fig. 6B
Full blot
Cont Perm1
Keap1
(Red)
75
Input(Total)
75
Keap1
130
Perm1
55
Tubulin
Keap1
(Red)
Keap1
Perm1
Tubulin

Fig. 6C

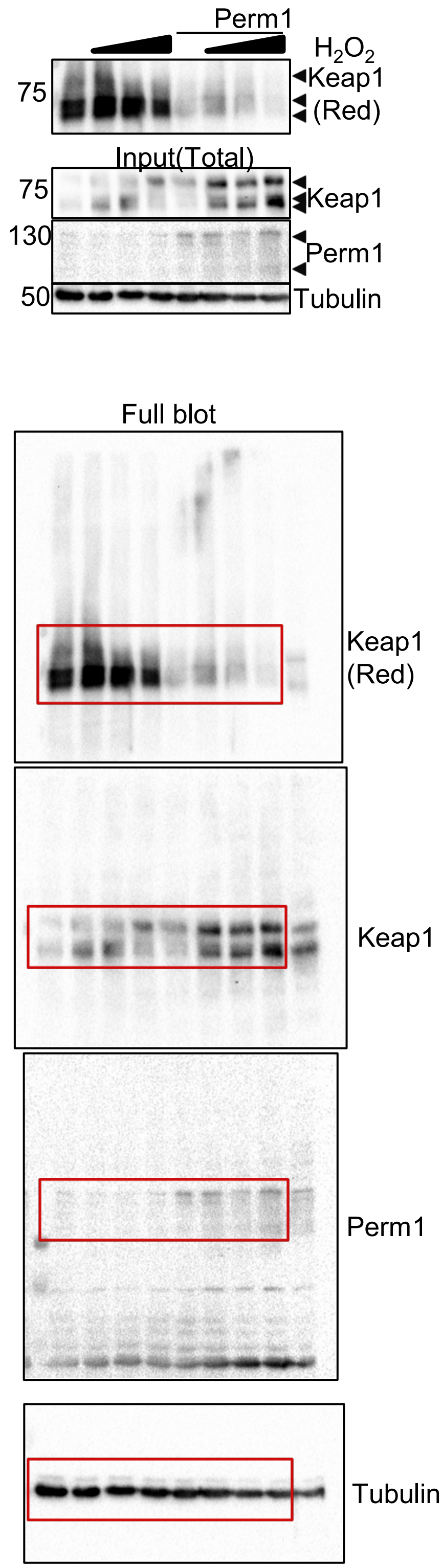

Perm1
H2O2
75
Keap1
(Red)
Input(Total)
75
Keap1
130
Perm1
50
Tubulin
Full blot
Keap1
(Red)
Keap1
Perm1
Tubulin

Fig. 6D

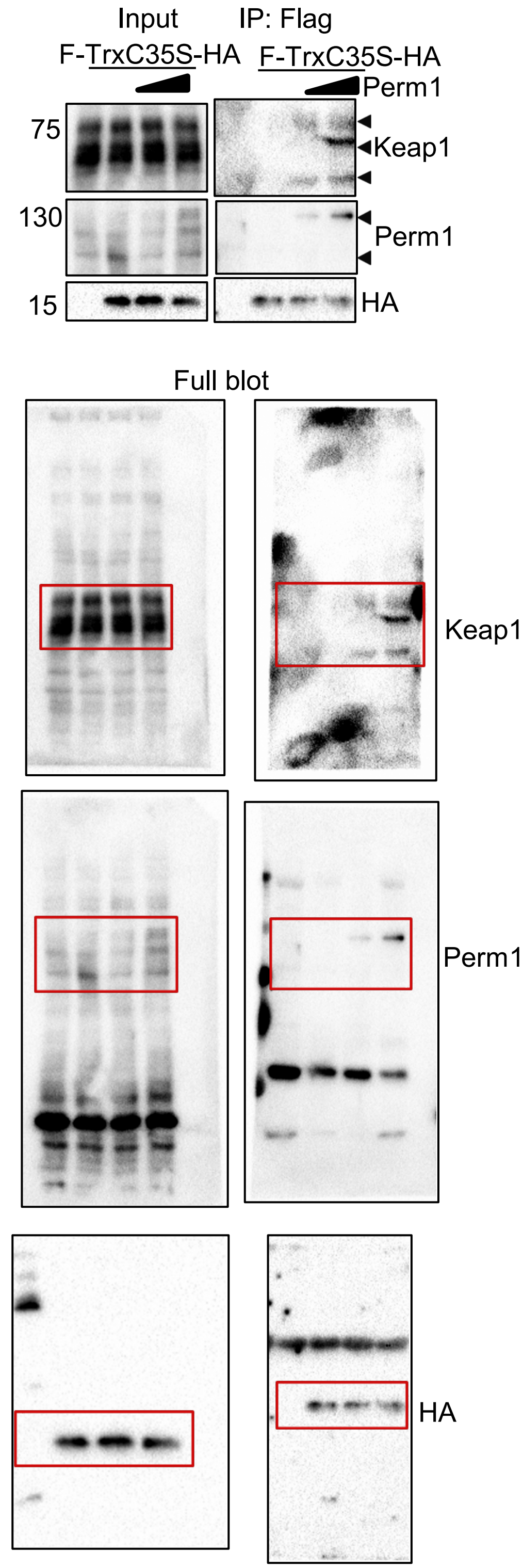

Input
IP: Flag
F-TrxC35S-HA
F-TrxC35S-HA
Perm1
75
Keap1
130
Perm1
15
HA
Full blot
Keap1
Perm1
HA

Fig. 6E

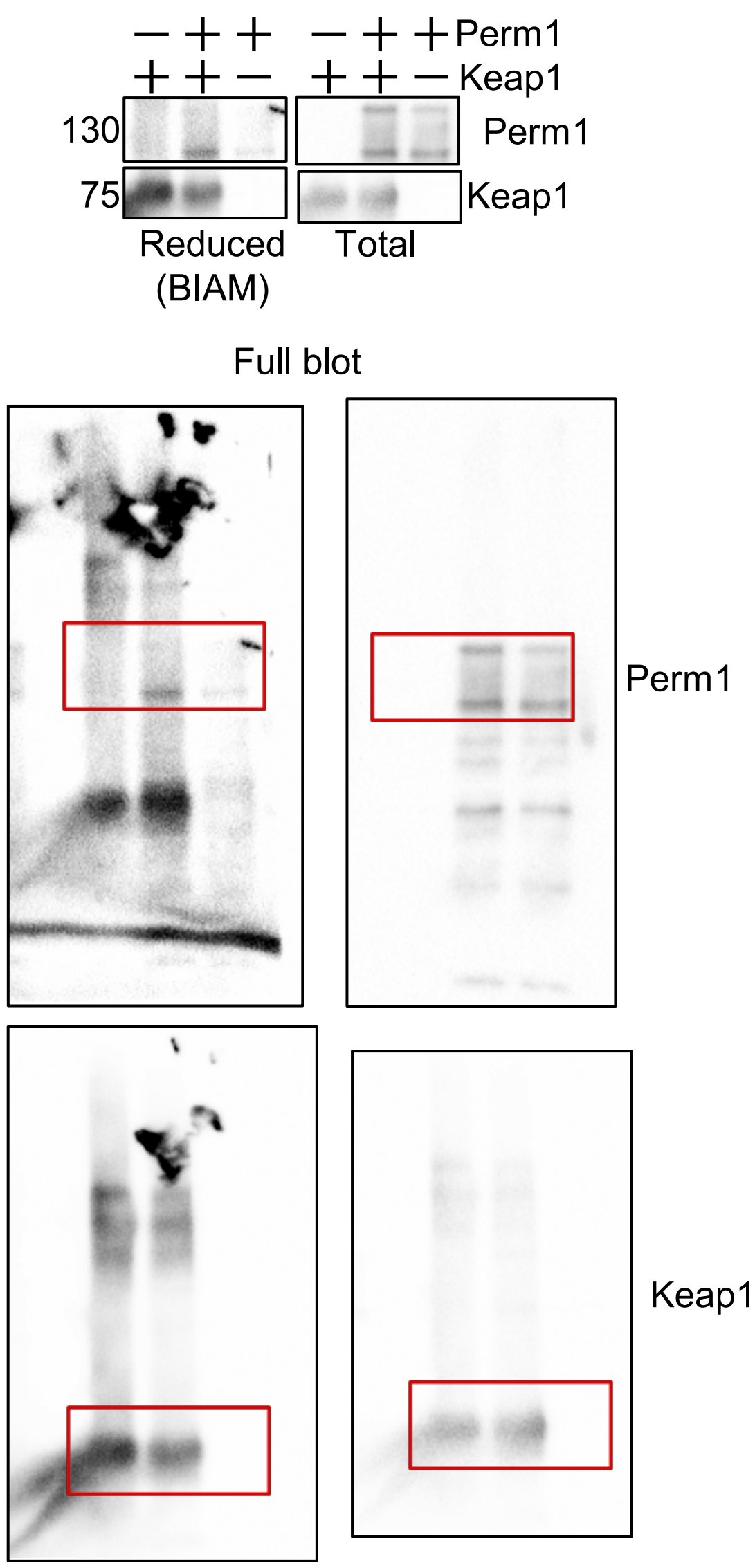
− + + − + + Perm1
+ + − + + − Keap1
130
Perm1
75
Keap1
Reduced
(BIAM)
Total
Full blot
Perm1
Keap1

Fig. 6F

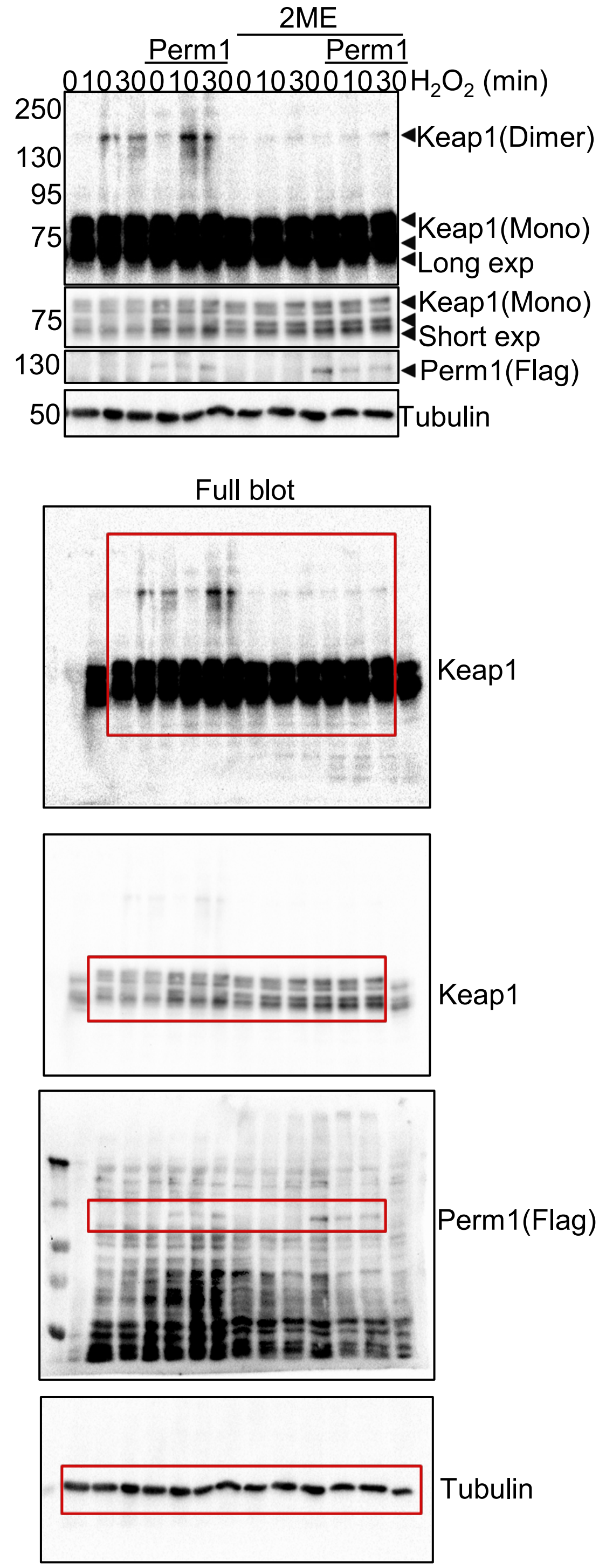
2ME
Perm1
Perm1
0 10 30 0 10 30 0 10 30 0 10 30 H_2O_2 (min)
250
130
95
75
Keap1(Dimer)
Keap1(Mono)
Long exp
75
Keap1(Mono)
Short exp
130
Perm1(Flag)
50
Tubulin
Full blot
Keap1
Keap1
Perm1(Flag)
Tubulin

Fig. 6G

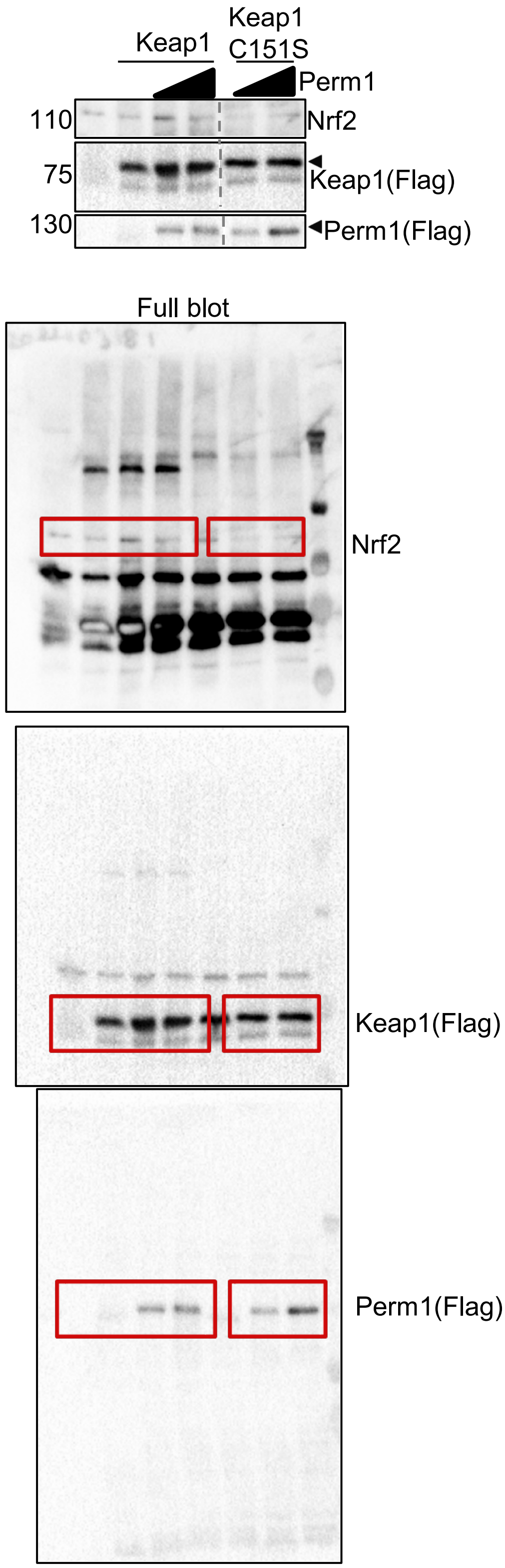

Keap1
Keap1 C151S
Perm1
110
Nrf2
75
Keap1(Flag)
130
Perm1(Flag)
Full blot
Nrf2
Keap1(Flag)
Perm1(Flag)

Fig. 7A

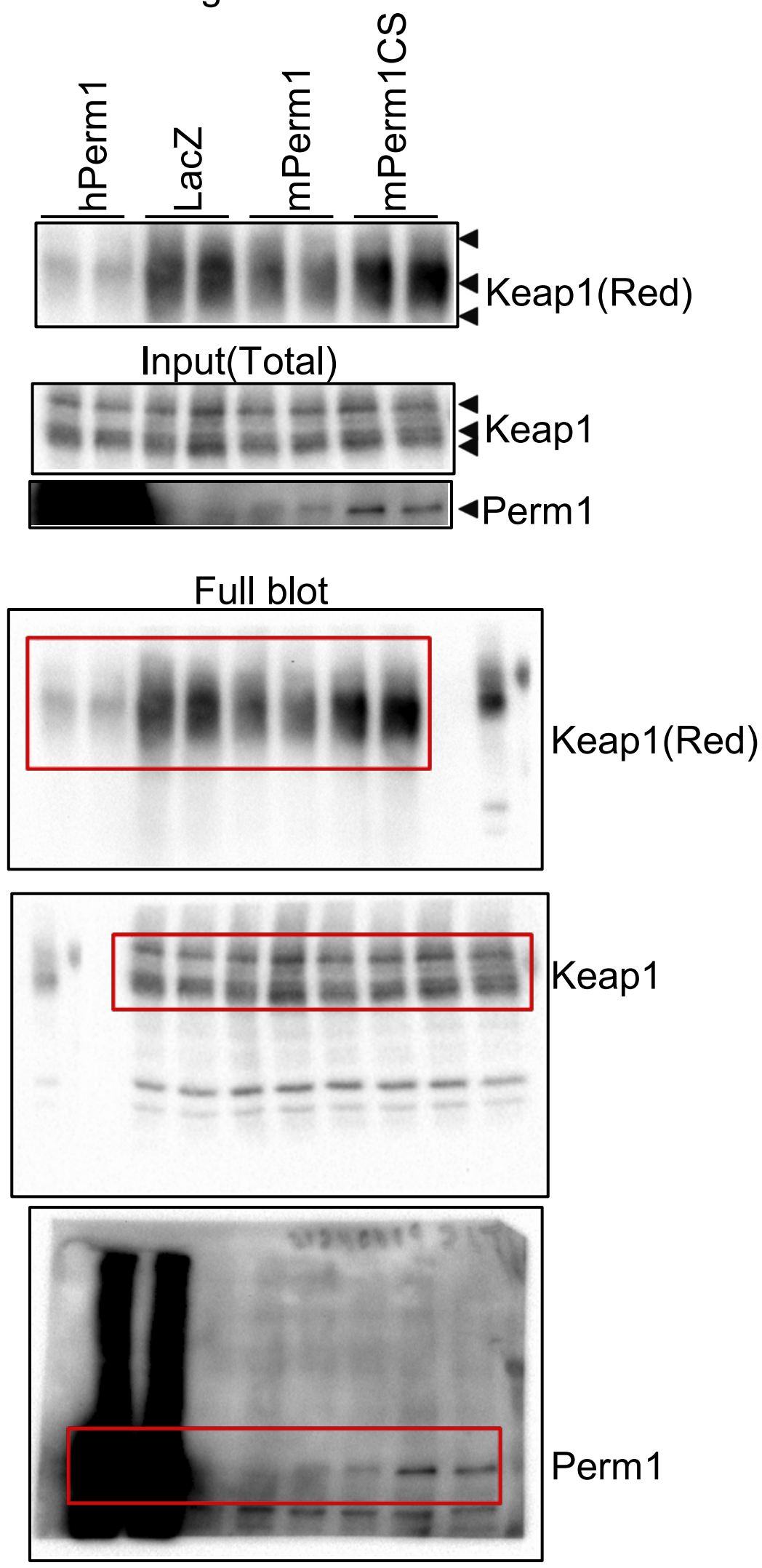

hPerm1
LacZ
mPerm1
mPerm1CS
Keap1(Red)
Input(Total)
Keap1
Perm1
Full blot
Keap1(Red)
Keap1
Perm1

Fig. 7B

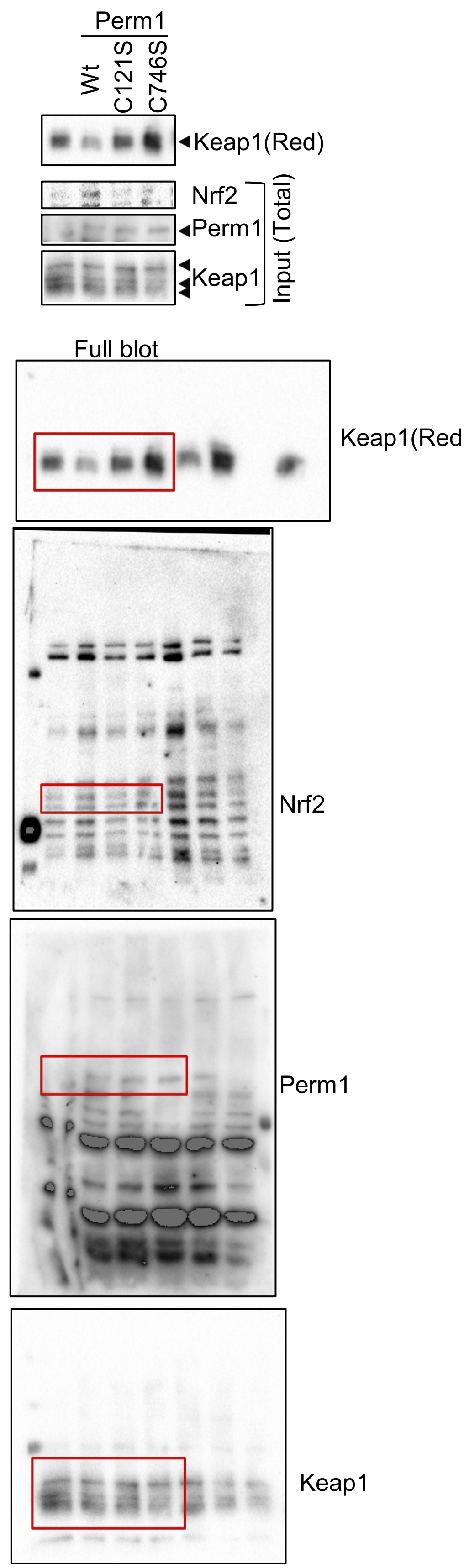

Perm1
Wt
C121S
C746S
Keap1(Red)
Nrf2
Perm1
Keap1
Input (Total)
Full blot
Keap1(Red)
Nrf2
Perm1
Keap1

Fig. S1A

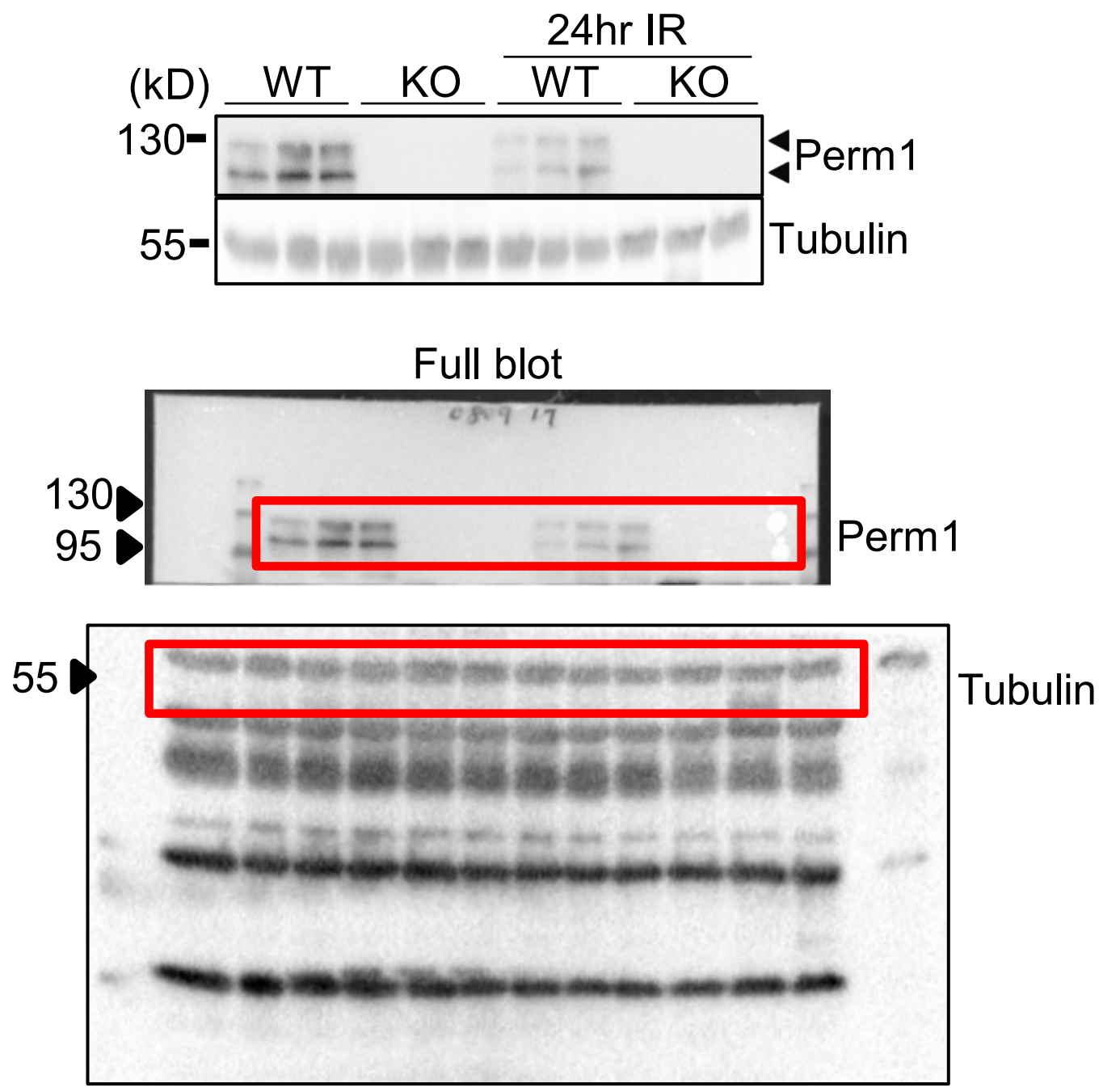

Fig. S2A

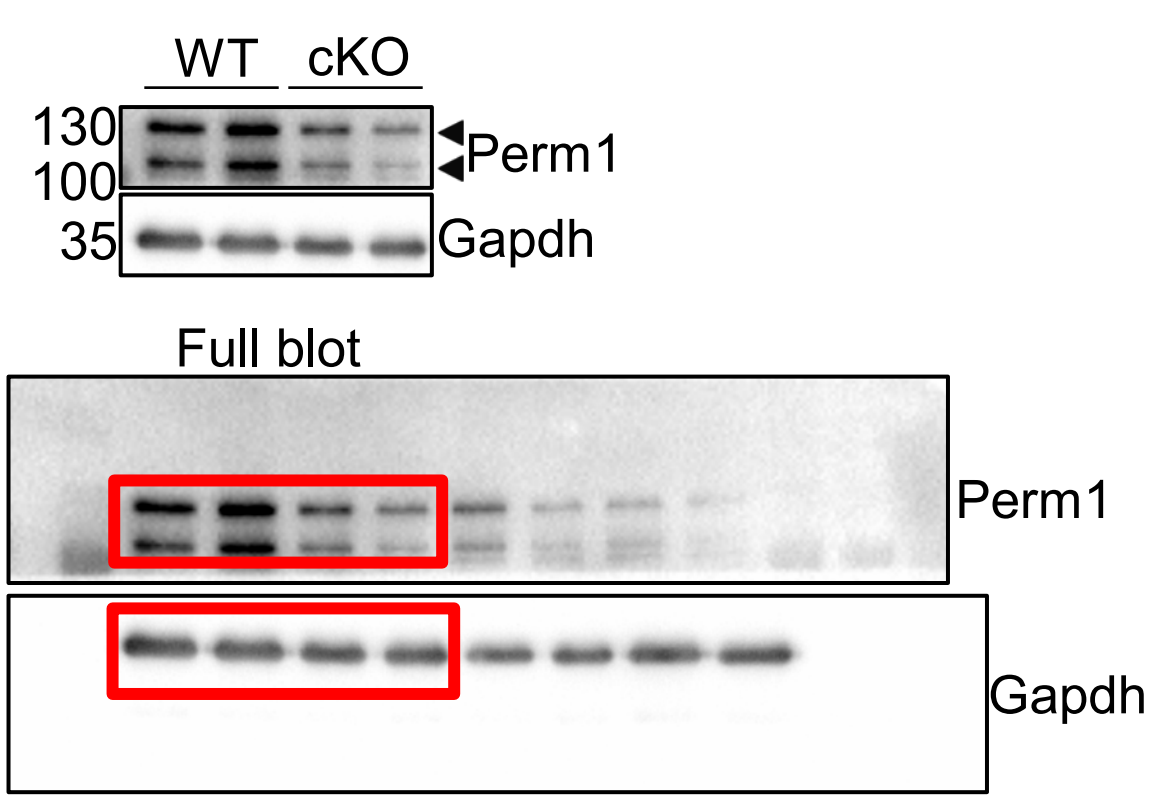

Fig S4A

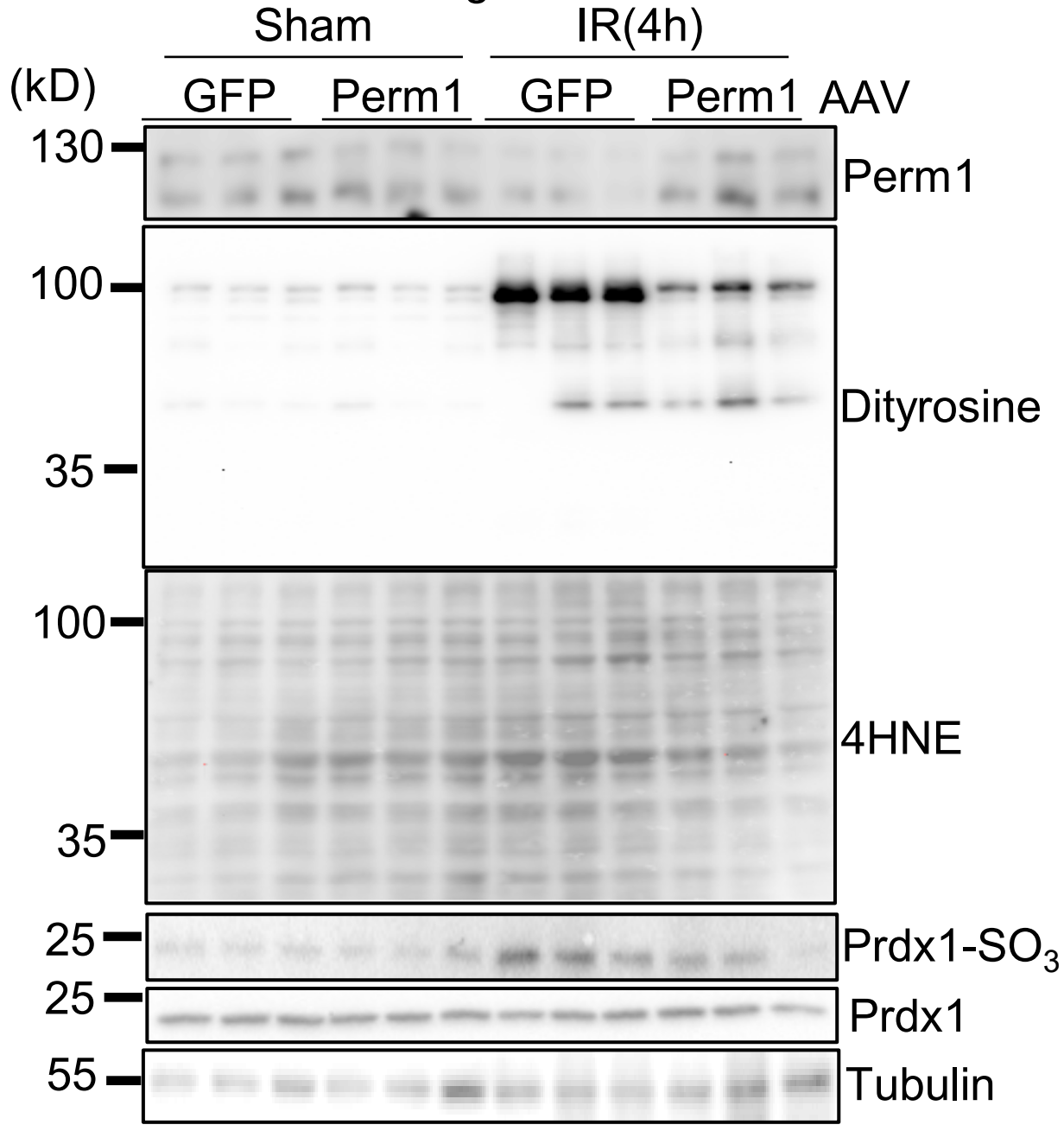

Full blot

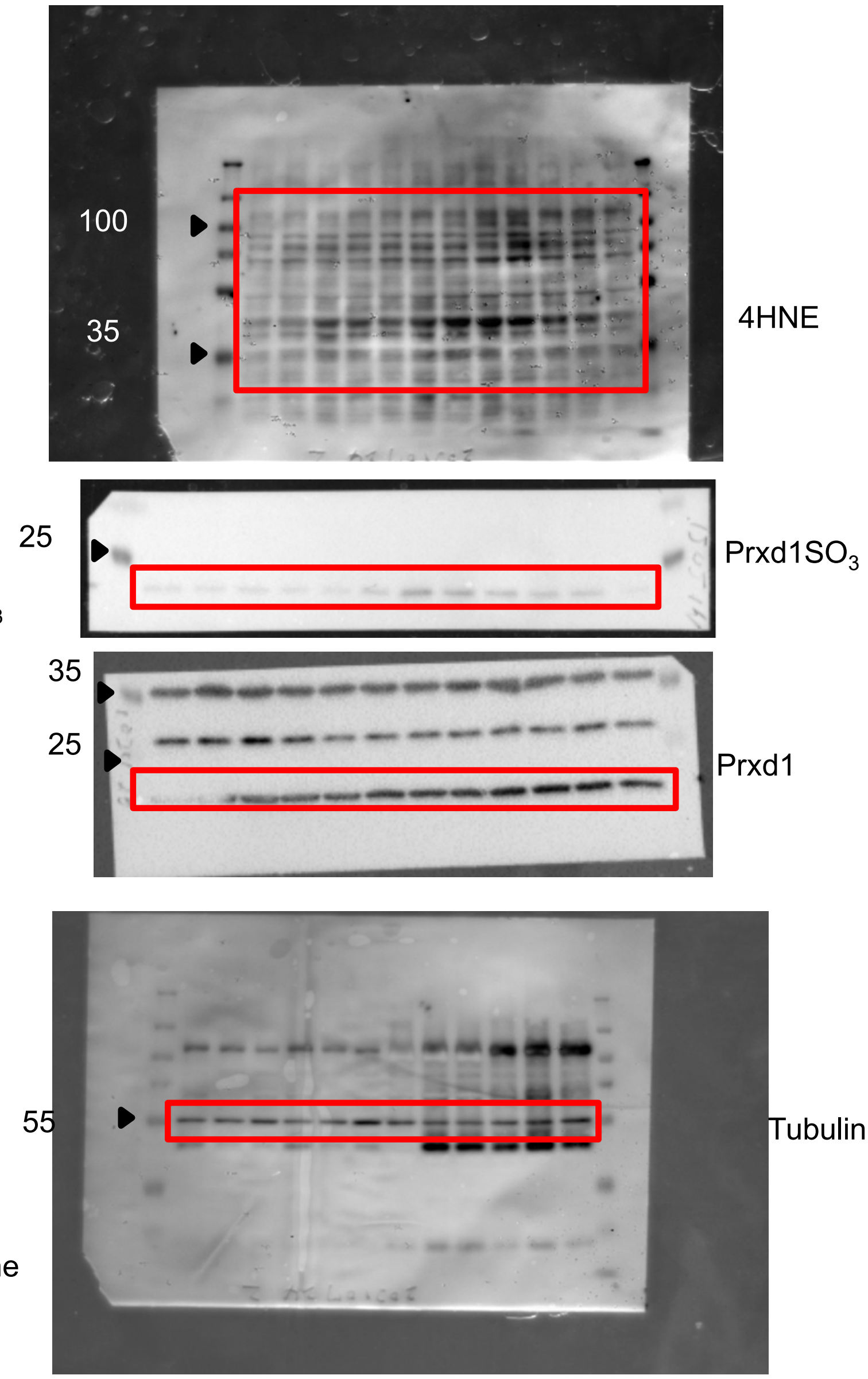

Full blot

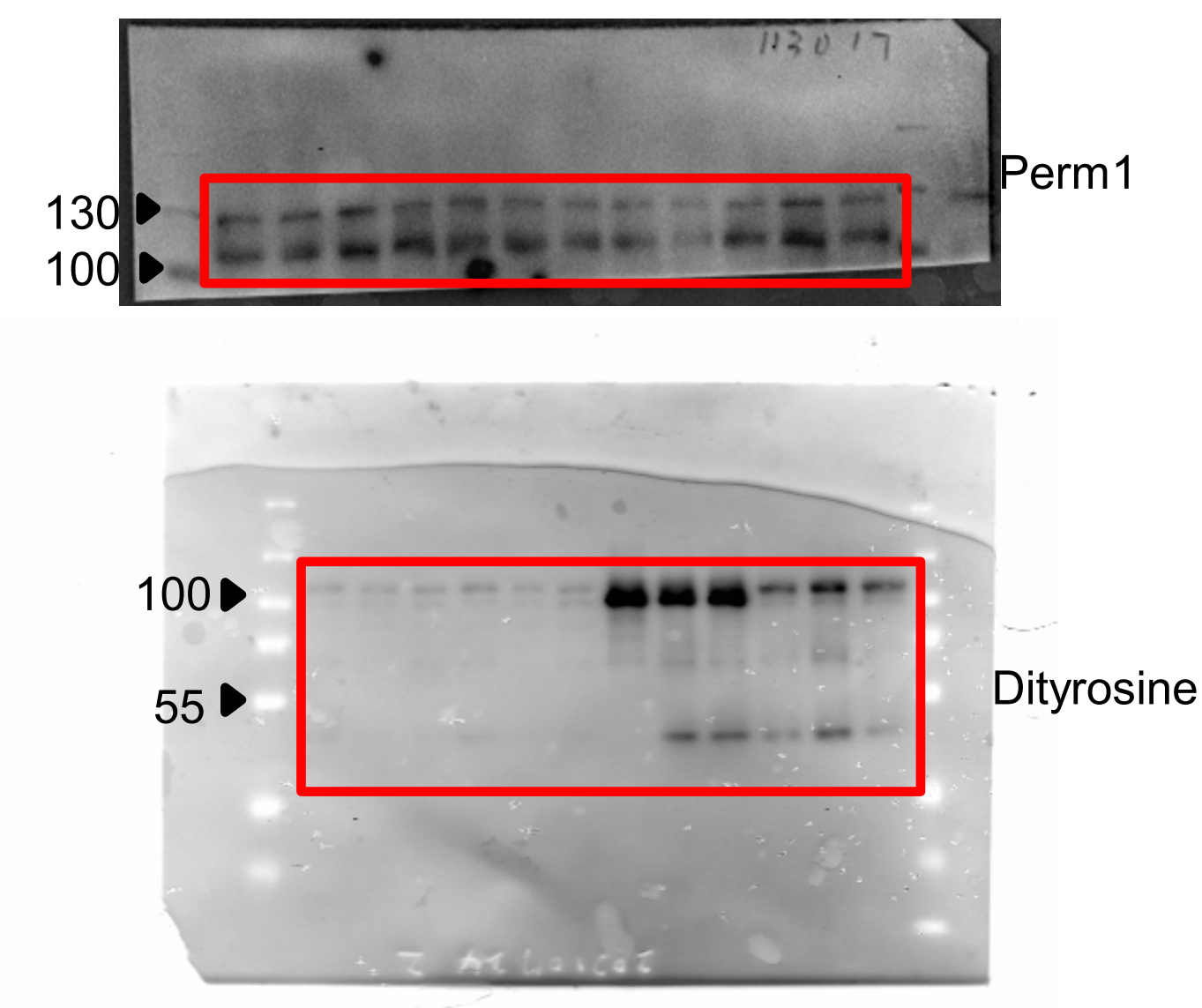

Fig S4B

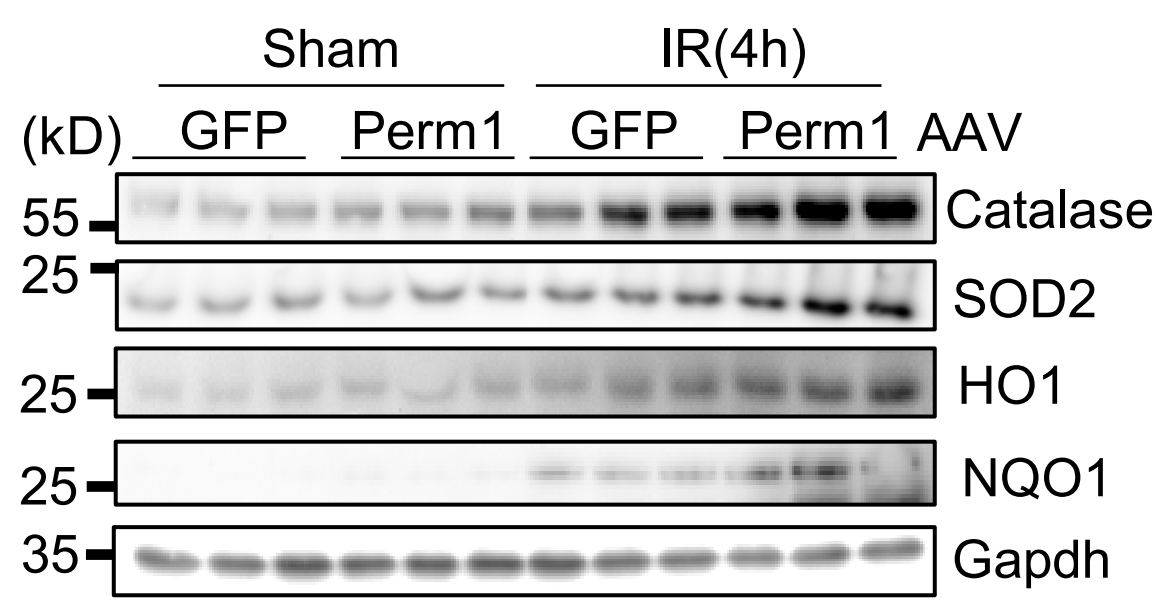

Full blot

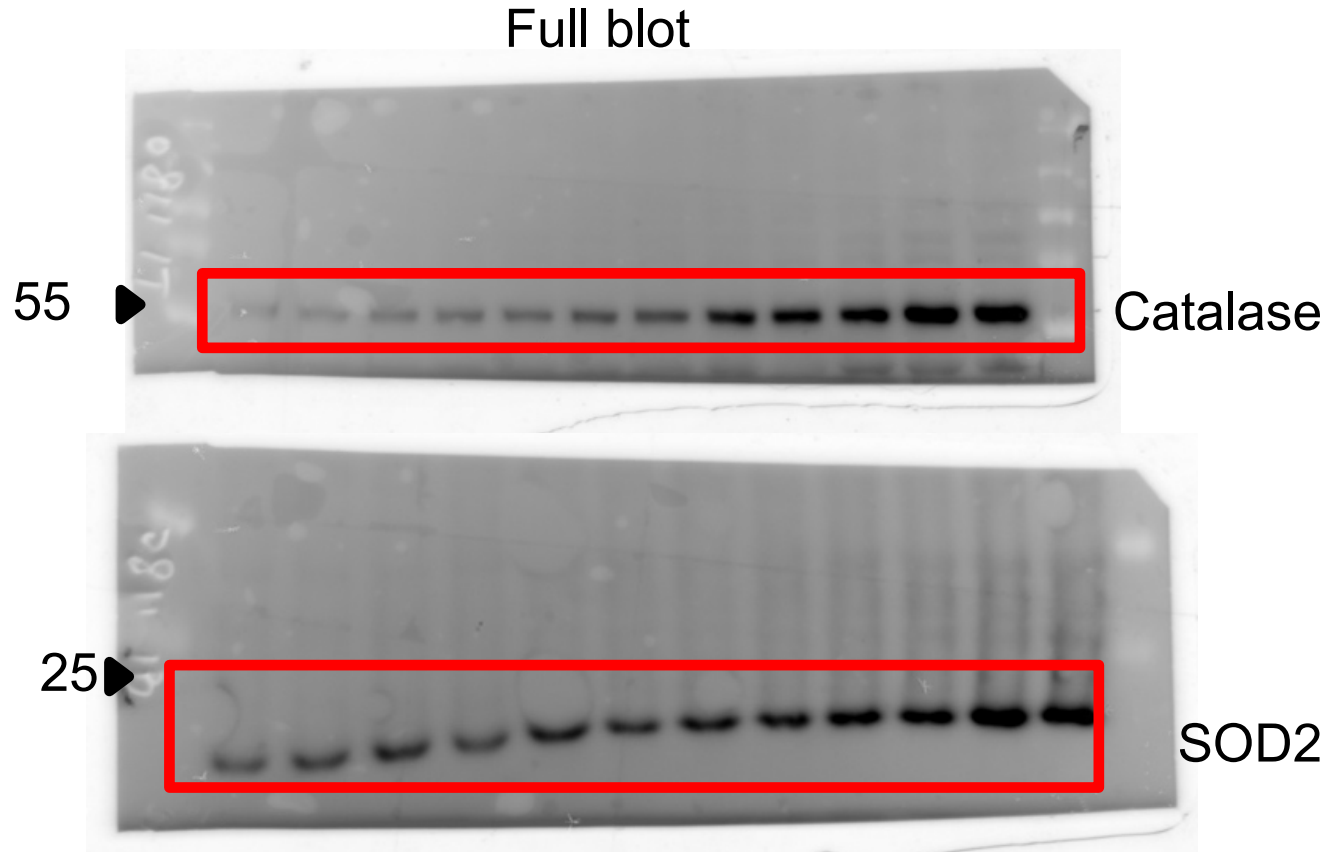

Full blot

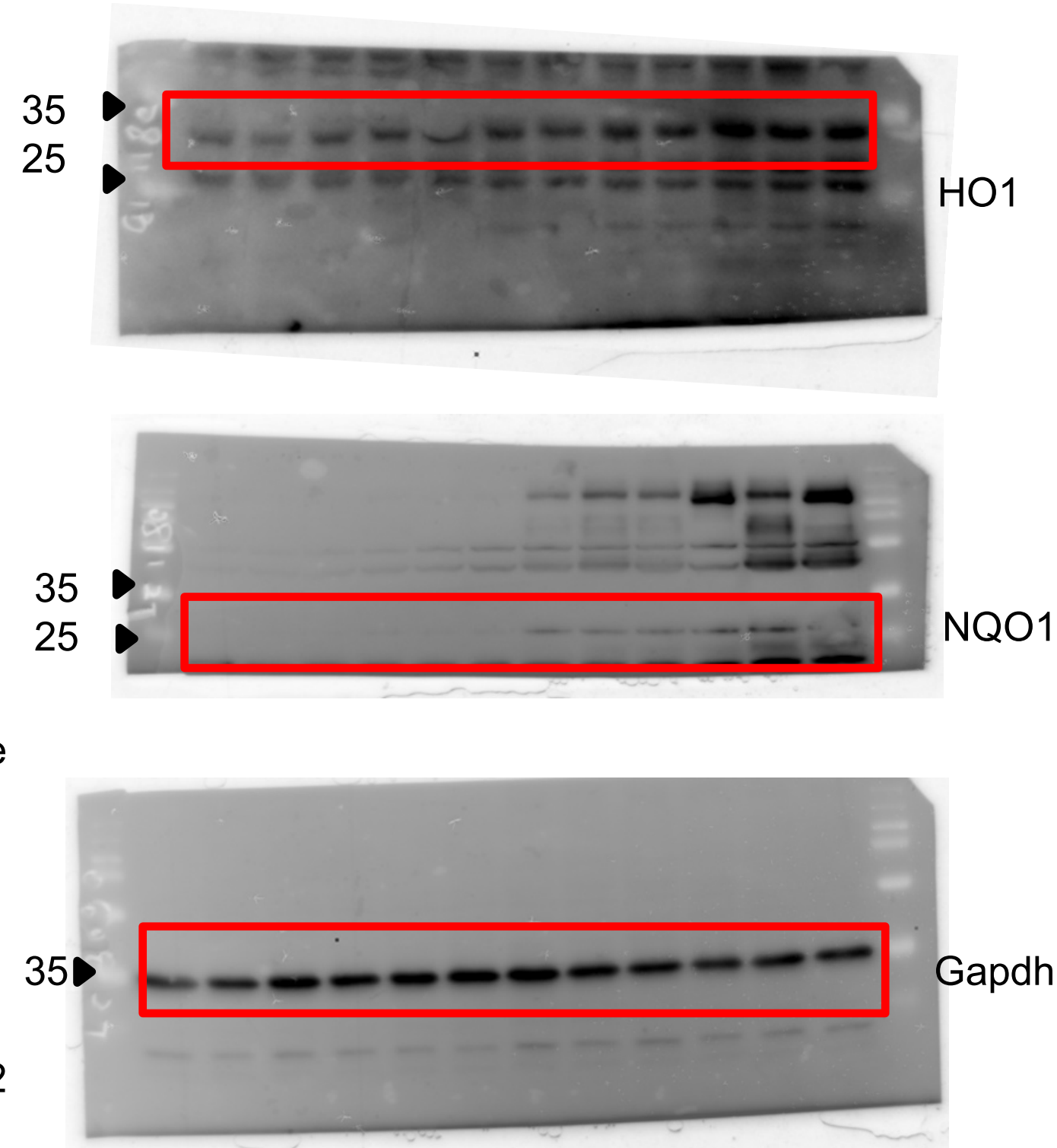

Fig. S5C

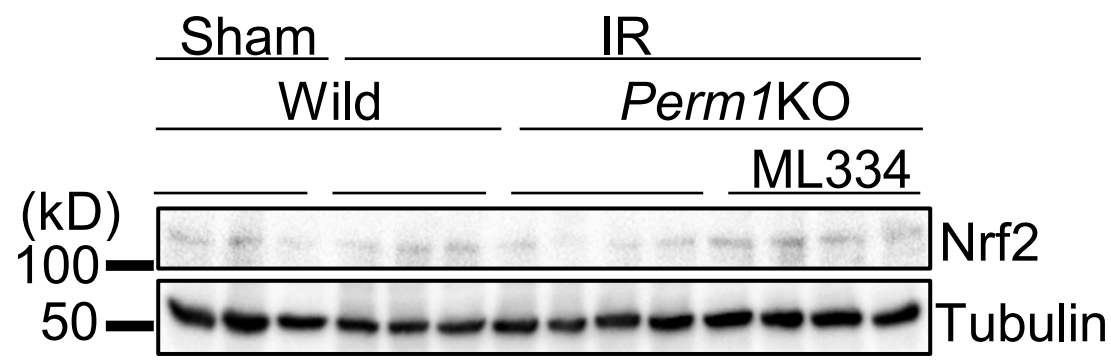

Full blot

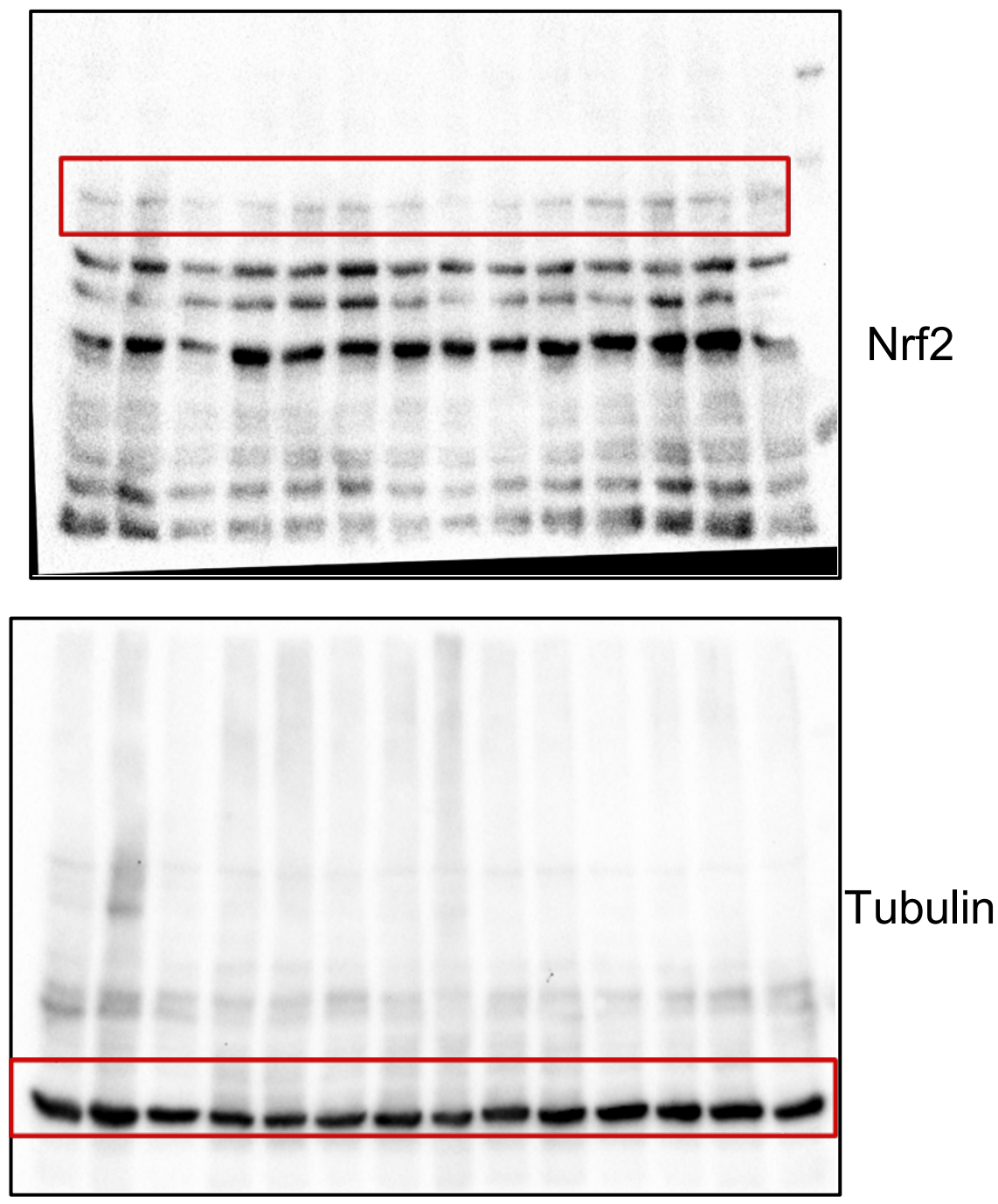

Fig. S5E

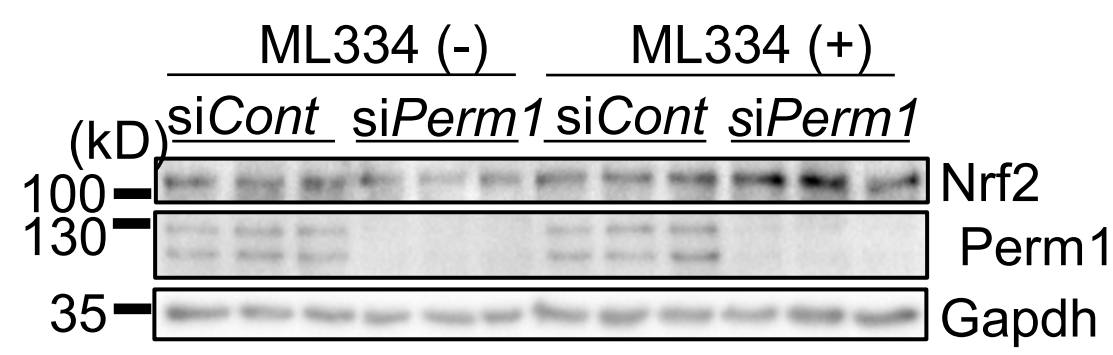

Full blot

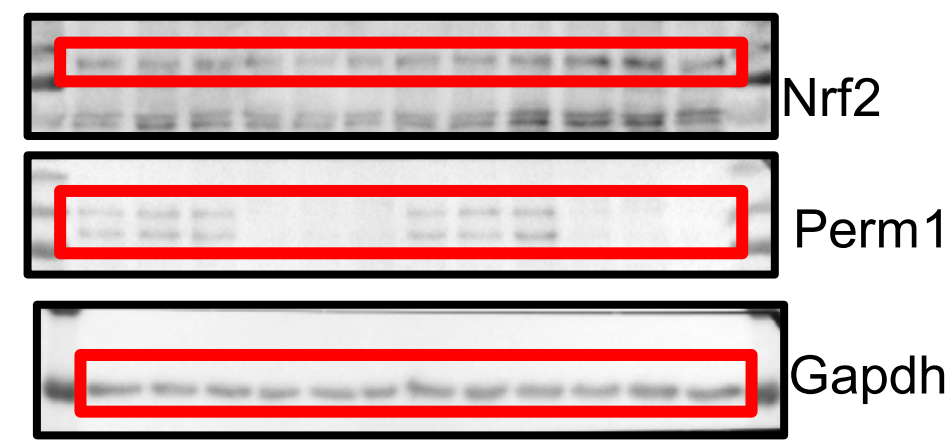

Fig. S6

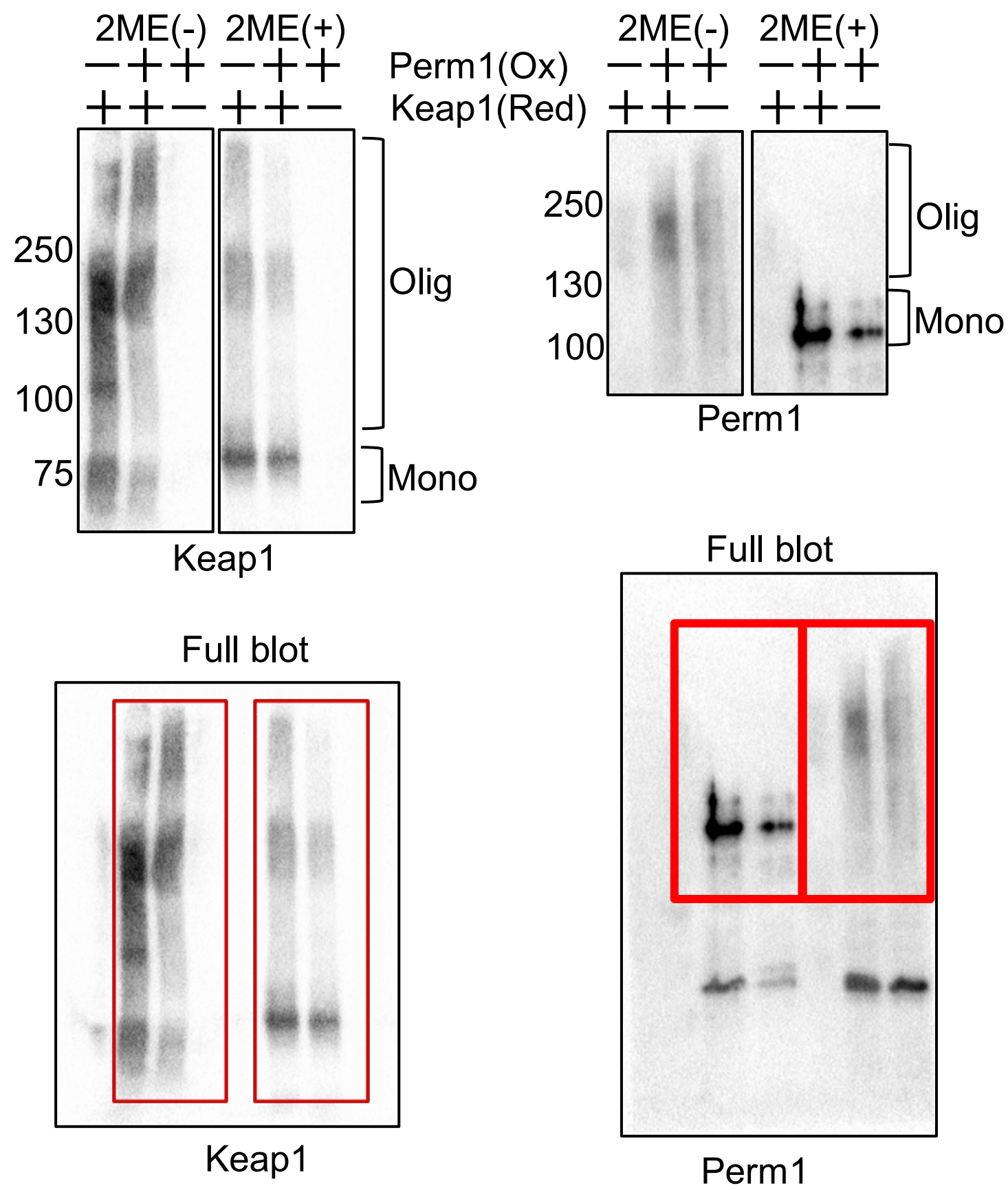

2ME(-) 2ME(+)
Perm1(Ox)
Keap1(Red)
250
130
100
75
Olig
Mono
Keap1
2ME(-) 2ME(+)
250
130
100
Olig
Mono
Perm1
Full blot
Keap1
Full blot
Perm1
